# Clinical Relevance of Targeted Therapy and Immune-Checkpoint Inhibition in Lung Cancer

**DOI:** 10.3390/pharmaceutics15041252

**Published:** 2023-04-16

**Authors:** Gian Marco Leone, Saverio Candido, Alessandro Lavoro, Silvia Vivarelli, Giuseppe Gattuso, Daniela Calina, Massimo Libra, Luca Falzone

**Affiliations:** 1 Department of Biomedical and Biotechnological Sciences, University of Catania, 95123 Catania, Italy; 2 Research Center for Prevention, Diagnosis and Treatment of Cancer, University of Catania, 95123 Catania, Italy; 3 Department of Biomedical and Dental Sciences, Morphological and Functional Imaging, Section of Occupational Medicine, University of Messina, 98125 Messina, Italy; 4 Department of Clinical Pharmacy, University of Medicine and Pharmacy of Craiova, 200349 Craiova, Romania; 5 Epidemiology and Biostatistics Unit, Istituto Nazionale Tumori IRCCS “Fondazione G. Pascale”, 80131 Naples, Italy; l.falzone@istitutotumori.na.it

**Keywords:** lung cancer, targeted therapy, tyrosine kinase inhibitors, immune-checkpoint inhibitors, microbiome, dysbiosis

## Abstract

Lung cancer (LC) represents the second most diagnosed tumor and the malignancy with the highest mortality rate. In recent years, tremendous progress has been made in the treatment of this tumor thanks to the discovery, testing, and clinical approval of novel therapeutic approaches. Firstly, targeted therapies aimed at inhibiting specific mutated tyrosine kinases or downstream factors were approved in clinical practice. Secondly, immunotherapy inducing the reactivation of the immune system to efficiently eliminate LC cells has been approved. This review describes in depth both current and ongoing clinical studies, which allowed the approval of targeted therapies and immune-checkpoint inhibitors as standard of care for LC. Moreover, the present advantages and pitfalls of new therapeutic approaches will be discussed. Finally, the acquired importance of human microbiota as a novel source of LC biomarkers, as well as therapeutic targets to improve the efficacy of available therapies, was analyzed. Therapy against LC is increasingly becoming holistic, taking into consideration not only the genetic landscape of the tumor, but also the immune background and other individual variables, such as patient-specific gut microbial composition. On these bases, in the future, the research milestones reached will allow clinicians to treat LC patients with tailored approaches.

## 1. Introduction

Lung cancer (LC) represents the second most frequently diagnosed cancer worldwide for both sexes. Despite the acquisition of new diagnostic, prognostic, and therapeutic approaches, LC is still responsible for 127,070 deaths every year in the United States, representing the leading cause of cancer-related death [1].

LC is classified into different subtypes based on its morphological and histological heterogeneity [2]. Currently, LC can be classified as small cell lung carcinoma (SCLC), representing 10–15% of LC cases, and non-small cell lung carcinoma (NSCLC), which is the most represented form (80–85% of LC diagnoses). NSCLC can be further grouped into three main histological subtypes: adenocarcinoma (ADC), squamous carcinoma (SqCC), and large cell carcinoma (LCC) [3,4,5]. Additionally, less common types of NSCLC exist, including adenosquamous carcinoma (ADSC), sarcomatous carcinoma (SCC), salivary gland carcinoma, carcinoid tumor, and other unclassified rare forms [2,6,7].

ADC accounts for about 60% of NSCLCs and 40% of all LC. It originates from the malignant transformation of the airway mucus-secreting cells. ADC usually develops in the peripheral portion of the lung with frequent central fibrosis and pleural involvement. In most cases, this tumor can be accompanied by chronic infections or by the formation of extended lung necrotic areas [8]. In 2011, a new classification of ADC was proposed by the main international scientific societies (including the International Association for the Study of Lung Cancer, IASLC; the American Thoracic Society, ATS; and the European Respiratory Society, ERS) and this was accepted by the WHO in 2015 [5,6]. In addition to invasive ADC, the updated classification considers pre-invasive lesions (such as atypical adenomatous hyperplasia, mucinous and non-mucinous ADC in situ) and minimally invasive ADC (with a diameter of less than 3 cm, lepidic growth and infiltration with concurrent formation of lesions of less than 5 mm) [9].

Invasive ADC includes various patterns of development belonging to three diverse prognostic groups: the lepidic pattern (Grade 1), the acinar and papillary pattern (Grade 2), and the micropapillary and solid pattern (Grade 3). Given the mixed histology presented by ADC, the pathological diagnosis is formulated by reporting the percentages of each singular histological component [8]. 

SqCC usually affects the central region of lungs, developing inside the bronchial tree, occluding it, and further extending to the proximal bronchi and outwards in the nearby lung tissue. The clinical assessment of SqCC is mainly performed using a bronchoscopy biopsy with cytology and spontaneous pre- and post-bronchial exfoliation [10]. Based on the presence or the absence of keratinization, intercellular bridges, and hyperchromatic nuclei, SqCC can be classified into keratinizing, non-keratinizing, and basaloid subtypes. The basaloid subtype is associated with a poorer prognosis due to the development of intrinsic chemotherapy refractory recurrences [8]. 

LCC represents 3% of LC, and it develops within the peripheral area of the lung with a subpleural involvement. It differs from the other LC types with the peculiar presence of polygonal cells with large nuclei surrounded by small cytoplasm. LCC diagnosis is usually made by exclusion, being poorly differentiated. In recent years, there has been a steady increase in ADC frequency in western countries, whereas SqCC and SCLC frequency has decreased [11].

Differently from NSCLC, SCLC shows neuroendocrine as well as epithelial characteristics. It usually develops in the central thoracic position with the typical involvement of the bronchi. SCLC shows a rapid growth and an early metastatic dissemination. At the time of diagnosis, about 60–70% of patients already show extra-thoracic spread [12]. From a histological point of view, SCLC is characterized by small cells with scant cytoplasm, finely granular chromatin, low nucleoli, and a high mitotic rate [13]. Histological examination is essential to discriminate and confirm the diagnosis of LC, and it is performed from biopsies through the use of bronchoscopy, mediastinoscopy, or thoracoscopy, depending on the location of the primary tumor [14].

Generally, the histological diagnosis of LC is based on the nuclear characteristics, including the presence of finely dispersed chromatin, the absence of nucleoli, the high mitotic rate, and the presence of apoptotic debris within the transformed tissue. Currently, the diagnosis of LC is performed by careful morphological and molecular evaluations through immunohistochemistry (IHC), fluorescence in situ hybridization (FISH), polymerase chain reaction (PCR), and, more recently, pyrosequencing and next-generation sequencing (NGS) [15]. Cytological samples are characterized by the presence or absence of specific markers, including TTF-1, CD56, p40, p63, Ki67, INSM1, napsin A, mucin, chromogranin, synaptophysin, and low molecular weight cytokeratin [5,13,16,17]. 

The histological assessment of LC allows clinicians to predict the prognosis of patients and the best therapeutic option available. The five-year survival rate ranges from 5% to 17% depending on the stage of tumor, the age, the sex of the patient, and the geographic variation [18,19,20]. Although research has made important advances in diagnosis, prognosis, and therapy, LC remains a neoplasm with poor prognosis, typically due to a late diagnosis coupled with the lack of effective diagnostic/prognostic biomarkers [21]. 

Several risk factors have been associated with an increased risk of LC. Of these, tobacco smoking represents the most important risk factor in LC, and it is associated with 80–90% of all LC cases [22]. Other risk factors are represented by occupational and environmental factors including radon, nickel, uranium, ionizing radiation, pesticide, and asbestiform fibers with a dose-dependent association [23,24,25]. Consequently, smoking cessation seems to be associated with a reduced risk of LC in people exposed to occupational/environmental additional factors [23]. 

In the past, LC was mainly treated with platinum-based chemotherapy [26]. Recently, new agents contributed to a significant increase in the 5-year survival rate of patients. Specifically, targeted therapy is based on the administration of selective inhibitors and biological molecules able to contrast LC cells growth by interfering with specifically targeted receptors or other downstream proteins. Currently, several genetic alterations occurring in LC have been identified, including EGFR, ALK, ROS1, KRAS, BRAF, and HER2 in ADC, as well as PI3K, PTEN, MET, and DDR2 in other subtypes of LC [27].

Additionally, targeted immunotherapy has been developed ensuring the activation of the immune system against LC cells. In particular, immune checkpoint inhibitors (ICIs) are humanized monoclonal antibodies (mAbs) designed to bind and block the so-called immune receptors, which are expressed by both LC cells and immune cells. These include the cytotoxic T-lymphocyte antigen-4 (CTLA-4), programmed death-1 (PD-1), and its ligands programmed death ligand 1 and 2 (PD-L1 and PD-L2) [28,29,30].

Due to the large number of drugs currently available, this review discusses in depth the mechanism of action as well as the rationale behind the use of targeted therapies and ICIs for the treatment of LC. Additionally, among the novel sources of predictive biomarkers, the role of lung and gut microbiota in LC is also analyzed. The association of the modulation of intestinal microbiota with such novel therapies is currently a pursued strategy in clinics to decrease the toxicity as well as to ameliorate the therapeutic outcome of LC patients. 

## 2. Molecular Landscape of Lung Cancer and Targeted Therapy

Previously, cytotoxic chemotherapy was the only treatment option available for advanced LC patients, either inoperable or presenting recurrence. At the beginning of 2000, the identification of specific genetic alterations improved the management of LC patients, leading to a personalized anticancer approach [31]. These targeted therapies soon became the standard of care, leading to better clinical outcomes in specific subsets of LC patients. Indeed, targeted therapies increased both overall survival and quality of life of LC patients, showing higher efficacy and low toxicity compared to standard chemotherapy [31]. 

**Figure 1:**
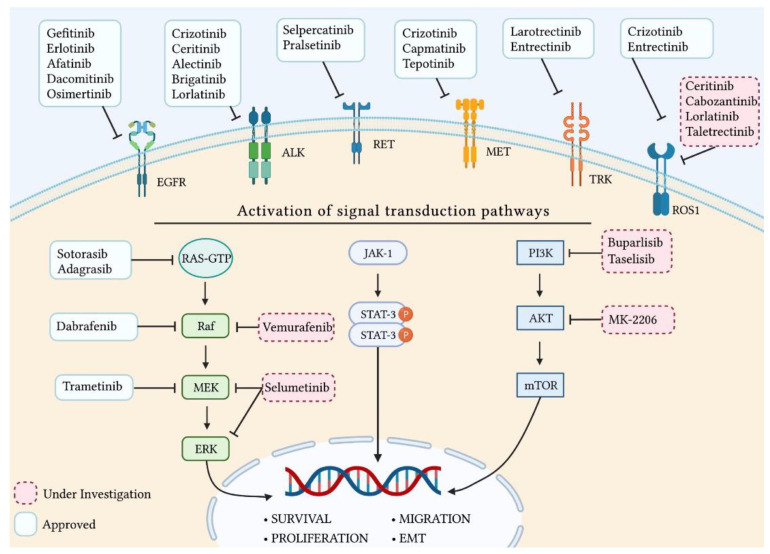
Targeted therapies: molecular targets and inhibited pathways. Protein kinase inhibitor targets are (1) membrane serine/threonine protein kinase: EGFR inhibitors, ALK inhibitors, RET inhibitors, MET inhibitors, NTRK inhibitors, and ROS1 inhibitors (from left to right); and (2) intracellular downstream proteins of the signal transduction pathways: KRAS inhibitors, RAF inhibitors, MEK-ERK inhibitors, PI3K inhibitor, and AKT inhibitor. The inhibition of both membrane and intracellular targets leads to the inhibition of the following pathways: (1) MAPKs (left column: RAS-GTP, Raf, MEK, ERK), (2) JAK/STAT (middle column: JAK-1, STAT3), and (3) PI3K/AKT (right column: PI3K, AKT, mTOR). Blue boxes indicate FDA-approved drugs, red dotted boxes indicate drugs under investigation (not yet approved for clinical usage).

Currently, several molecular alterations have been identified in LC, some of which represent a therapeutic target to targeted treatments [32]. At present, only testing for EGFR mutations and ALK or ROS1 rearrangements are routinely performed in clinics while other emerging targets are still under investigation [27,33,34]. Figure 1 summarizes all the targeted therapies (available and under investigation) and their main molecular targets within the LC cell.

One of the major advantages of targeted therapies is that they are based on the administration of small molecules with an oral and well-tolerated administration. Additionally, given the specificity of targeting, the toxicity is reduced compared to standard chemotherapy and radiotherapy, positively influencing the quality of life of the patient. In the following paragraphs, the key clinical trials for the characterization and approval of several targeted therapies are discussed. Supplementary Table S1 displays the currently ongoing trials aiming to assess the safety and efficacy of targeted therapies directed towards pivotal mutated TK oncogenes in LC, alone and in combination. 

### 2.1. Epidermal Growth Factor Receptor

The epidermal growth factor receptor (EGFR) gene is located on chromosome 7. It consists of 28 exons and it encodes for a transmembrane glycoprotein with a cytoplasmic tyrosine kinase domain, belonging to the family of ErbB receptors [35]. The EGFR binds a 53 amino acid-long protein ligand known as epidermal growth factor (EGF). EGF binding to EGFR determines the EGFR activation upon homodimerization or heterodimerization with other receptors belonging to the same family [36]. EGFR dimerization causes the autophosphorylation of the tyrosine kinase domains, which through downstream signaling effector proteins activates many signaling pathways, including the MAPK, JAK/STAT, PI3K/Akt/mTOR, and PLC-γ1 pathways (Figure 1) [37]. 

Importantly, several EGFR gene mutations lead to its overexpression in LC. Specifically, EGFR mutations have been found in 40–60% of all diagnosed NSCLC. These mutations usually occur in Southeast Asian women who have never smoked [37]. The most commonly observed mutations occur in exons 18–21, which encode for the kinase domain of the receptor [38,39]. A total of 90% of the EGFR mutations in NSCLC are either deletions in the exon 19 (Ex19del) or a specific point mutation located within the exon 21 (L858R) [40]. The remaining 10% of the observed EGFR mutations are a heterogeneous group of molecular alterations affecting exon 18 (e.g., G719X) and exon 20 (e.g., T790M) [41,42]. All the listed EGFR gene mutations lead to an increased kinase activity and, consequently, to the excessive activation of the EGFR-associated pathways.

Currently, the FDA-approved EGFR tyrosine kinase inhibitors (TKIs) include the first-generation gefitinib (Iressa) and erlotinib (Tarceva), the second-generation afatinib (Giotrif) and dacomitinib (Vizimpro), and the third-generation osimertinib (Tagrisso). Overall, these molecules represent novel treatments for patients with advanced NSCLC with the most common EGFR mutations [43,44]. The pivotal published clinical studies are described below.

Erlotinib is a competitive EGFR-TKI that binds the ATP-binding site of EGFR mutated kinase domain, inhibiting the downstream signaling pathways [45]. EURTAC was a multicenter phase III study, whose results were published in 2012, which aimed to assess both the safety and efficacy of erlotinib versus chemotherapy in patients with EGFR-mutated advanced NSCLC [46]. The 86 patients treated with Erlotinib showed a median PFS of 9.7 months compared with 5.2 months for the standard chemotherapy-treated group. Regarding toxicity, only 6% of erlotinib-treated patients developed severe treatment-related adverse events (TRAEs) compared with 20% of chemotherapy-treated patients. Overall, the study supported the use of erlotinib for the treatment of EGFR-mutated NSCLC patients also in neoadjuvant regimens [46,47]. Specifically, in 2019, a single-arm phase II study was opened to assess the clinical benefits of erlotinib versus standard platinum-based chemotherapy as a neoadjuvant treatment. The results revealed that the twelve patients treated with erlotinib before tumor resection showed a median PFS of 12.1 months compared with 11.0 months observed for the eight chemotherapy-treated patients. Additionally, the patients treated with erlotinib showed an objective response rate (ORR) of 67%, a pathological response rate (PRR) of 67%, and an overall survival (OS) of 51.0 months compared with 19%, 38%, and 20.9 months observed in the chemotherapy-treated group, respectively [48].

Gefitinib is another EGFR-TKI that binds to the ATP binding site of the EGFR kinase domain, thus inhibiting the downstream pathways. In 2018, the safety and efficacy of gefitinib as an adjuvant treatment was evaluated in a single-center retrospective study compared to standard two-drug adjuvant chemotherapy in patients with stage II-IIIA NSCLC with occurring activating mutations in the TK domain of the EGFR gene [49]. The 52 patients treated with gefitinib showed a disease-free survival (DFS) of 34.9 months compared with 19.3 months observed in the adjuvant chemotherapy-treated group. Regarding toxicity, 15.4% of patients treated with gefitinib developed severe TRAEs compared with 38.5% of patients in the adjuvant chemotherapy group [49].

NEJ009 was a phase II study published in 2020 and aimed to assess both the safety and efficacy of administering gefitinib alone or in combination with chemotherapy (carboplatin plus pemetrexed) to 345 EGFR mutation-positive NSCLC patients [50]. 

The 172 patients treated with gefitinib plus chemotherapy showed a median PFS of 20.9 months, a median OS of 50.9 months, and an ORR of 84% compared with 11.9 months, 38.8 months, and 67% obtained in patients treated with gefitinib as a monotherapy. The rate of high-grade TRAEs was 31.0% in the gefitinib group and 65.3% in the combination group. These results demonstrated the higher response rate observed in patients treated with combination therapy compared to patients treated with gefitinib alone; however, higher toxicity was observed [50].

Afatinib and dacomitinib are two selective and irreversible inhibitors of the kinase domain of different ErbB family receptors, including EGFR. By binding to their target, they are able to block cell proliferation and induce cell death [51,52]. LUX-Lung 5 was a multicenter phase III study completed in 2014 which aimed to assess the efficacy of afatinib plus paclitaxel versus single-agent chemotherapy in patients with relapsed/refractory NSCLC with mutations occurring in the EGFR gene after erlotinib/gefitinib therapy [53]. 

The 134 patients treated with the combination therapy showed a median PFS of 5.6 months compared with 2.8 months for the 68 patients treated with chemotherapy alone. The ORRs were 32.1% and 13.2% for the combination-treated and chemotherapy-treated groups, respectively [53]. The median OS was similar in each group. Regarding the toxicity, 11.4% of the combination-treated group developed severe TRAEs compared with 3.3% of the single chemotherapy-treated group [53].

LUX-Lung 8 was a multicenter phase III study concluded in 2014 which aimed to assess the efficacy of afatinib versus erlotinib as a second-line treatment in lung SqCC. The study enrolled a total of 795 patients with Stage IIIB or IV lung SqCC who manifested a tumor progression after at least four cycles of platinum-based chemotherapy [54]. The 398 patients treated with afatinib showed a median PFS of 2.6 months compared with 1.9 months for the erlotinib-treated group. The OSs at the median follow-up of 18.4 months were 7.9 and 6.8 months in the afatinib- and erlotinib-treated groups, respectively. The observed DCR was 51% versus 40% in the afatinib- and erlotinib-treated groups, respectively, whereas no significant difference in ORR and manifested TRAEs was observed between the two groups [54]. Furthermore, a QoL questionnaire administered to the SqCC patients showed that 36% of the afatinib-treated subjects declared an improvement in their global health status and QoL compared with only the 28% of the erlotinib-treated subjects [55]. 

A retrospective study published in 2019 aimed to assess both the efficacy and safety of first-line afatinib versus gefitinib or erlotinib for patients with recurrent or metastatic NSCLC with occurring EGFR mutations. The 165 patients treated with afatinib showed a median PFS of 19.1 months compared with 13.7 and 14.0 months observed for the gefitinib- and erlotinib-treated groups, respectively. Regarding the toxicity, grade 3 or 4 TRAEs (i.e., diarrhea, paronychia, skin rush) were observed in 7.3% of the afatinib-treated patients (compared with 2.6% and 1.8% detected in the gefitinib- and erlotinib-treated patients, respectively). Overall, the study evidenced the better clinical efficacy of afatinib, with manageable toxicity, as a first-line treatment in NSCLC patients bearing mutations in the EGFR gene [56].

ARCHER 1050 is an ongoing randomized phase III trial, whose first results were published in 2017. The aim of the study is to assess both the efficacy and safety of administering dacomitinib versus gefitinib in previously untreated patients with advanced EGFR-mutated NSCLC [57]. The 227 patients treated with dacomitinib showed a median PFS of 14.7 months and an ORR of 76% with a median DOR of 15.9 months. On the other hand, the 225 patients treated with gefitinib showed a median PFS of 9.2 months and an ORR of 70% with a median DOR of 9.2 months. Regarding the toxicity, only 9% of the dacomitinib-treated patients developed serious TRAEs compared with 4% of the gefitinib-treated group. Given the promising results, dacomitinib should be considered as a first-line treatment for advanced NSCLC [57].

Additionally, during a median follow-up of 31.3 months, 103 (45.4%) and 117 (52.0%) deaths occurred in the dacomitinib and gefitinib groups, respectively. The dacomitinib-treated arm showed a median OS of 34.1 months compared with the 26.8 months observed for the gefitinib-treated arm [58]. A third report about ARCHER 1050 further confirmed that the OS benefit from first-line treatment with dacomitinib versus gefitinib is maintained after extended follow-up (47.9 months) in patients with advanced NSCLC with EGFR-activating mutations [59].

Upon 12 months of treatment with TKIs as first-line drugs, most NSCLC patients developed drug resistance associated with the appearance of novel EGFR-activating mutations, including the T790M point mutation. This additional EGFR gene mutation occurs in 60% of patients with NSCLC recurrence, and it is due to the conformational change which hinders the drug-binding pocket [60,61]. In 2018, osimertinib (Tagrisso, AZD9291) received FDA approval, becoming an additional first-line treatment option in advanced LC patients bearing the T790M EGFR-activating mutation [62]. The findings derived from the FLAURA study suggested that therapy with osimertinib has improved efficacy compared with standard EGFR-TKIs [62]. Notably, FLAURA is a multicenter phase III study which aimed to assess both the safety and efficacy of osimertinib in previously untreated advanced NSCLC patients with activating mutations of the EGFR gene [62]. The 279 patients treated with osimertinib showed a median PFS of 18.9 months, an ORR of 80%, and a DCR of 97% compared with EGFR-TKIs group that showed a median PFS of 10.2 months, an ORR of 76%, and a DCR of 92%. Regarding the toxicity, 22% of the patients treated with osimertinib developed serious TRAEs compared with 25% of patients with standard EGFR-TKIs [62]. The results concerning the long-term OS confirmed that the median OS was significantly higher for the osimertinib arm (38.6 months) than the EGFR-TKIs arm (31.8 months), further suggesting osimertinib as a novel standard of care for NSCLC patients with EGFR-activating mutations [63].

The discovery of activating mutations of the EGFR gene in NSCLC and the subsequent development of the TKIs belonging to the first (gefitinib, erlotinib), the second (afatinib, dacomitinib), and the third generation (osimertinib) has initiated the era of personalized medicine for the management of NSCLC. Tumor genotyping is now an essential step in identifying EGFR and other driver gene mutations/alterations and, hence, in tailoring the therapeutic choice around the patient. TKIs are currently employed as the standard of care for NSCLC harboring EGFR-activating mutations. Presently, osimertinib is preferred, given the higher survival benefits and the manageable toxicity. Additional ongoing trials may reveal the benefits of also using EGFR-TKIs as adjuvants and post-operative adjuvants (Supplementary Table S1).

### 2.2. Anaplastic Lymphoma Kinase

Anaplastic lymphoma kinase (ALK) is a transmembrane tyrosine kinase receptor. It is encoded by the human ALK gene, which is located in chromosome 2. The derived ALK protein product plays a key role in the development of the nervous system. ALK expression is higher during the embryonic stage, whereas it decreases in adults [64]. 

Rearrangements, point mutations, and amplifications of the ALK gene are often associated with the onset of a variety of cancers, including neuroblastoma, anaplastic large cell lymphoma, and NSCLC [65,66,67]. ALK gene rearrangements have been found in 3–7% of all diagnosed LC [68]. The most frequent ALK alteration is the ALK gene inversion, which generates the echinoderm microtubule-associated protein-like 4 (EML4)-ALK fusion gene [69]. EML4-ALK protein can activate several downstream pathways involved in cell survival and proliferation, including the JAK-STAT, the MAPK-ERK, and the PI3K-AKT pathways (Figure 1) [67,70]. 

Different variants of the EML4-ALK fusion gene have been observed in LC. These variants result from small inversions between ALK and different breakpoints on EML4 [71]. Regarding EML4-ALK fusion variants, new therapeutic options have been developed. Many studies have shown that different ALK inhibitors, including crizotinib (Xalkori), ceritinib (Zykadia), brigatinib (Alunbrig), and alectinib (Alecensa), possess higher therapeutic efficacy than platinum-based chemotherapy against metastatic NSCLC positive to EML4-ALK fusions [72,73,74,75]. 

Crizotinib acts as an ATP competitor on different protein targets, including ALK, ROS1, and MET [76]. PROFILE 1001 was a multicenter phase II study whose first results were published in 2010, which investigated both the safety and efficacy of crizotinib administered orally and daily in 28-day cycles to patients affected by NSCLC with occurring ALK rearrangements. The evaluated ORR was 57%, with a DCR at 8 weeks of 87% and an estimated 6-month PFS of 72%. Regarding the toxicity, the most common TRAEs observed were mild and included nausea and diarrhea. Only 5–6% of patients developed grade 3 or 4 ALT/AST increments [77]. Overall, these results allowed the approval of crizotinib for the treatment of patients with advanced ALK-positive NSCLC by the FDA in 2011 [78]. 

Additionally, PROFILE 1029 was a multicenter phase III study, whose results were published in 2018. The study assessed both the safety and efficacy of crizotinib in comparison with platinum-based chemotherapy (pemetrexed/cisplatin or pemetrexed/carboplatin) in East Asian patients with locally advanced, recurrent, or metastatic non-squamous NSCLC with occurring translocation or inversion involving ALK [79]. The patients treated with crizotinib showed a median PFS of 11.1 months compared with 6.8 months for patients treated with standard chemotherapy. Furthermore, the patients treated with crizotinib showed an ORR of 87.5% versus 45.6% in the chemotherapy-treated arms. The DCR at 12 weeks was 82.7% for the critozinib arm compared with 73.8% for the chemotherapy-treated group. The median OS was 28.5 months for the crizotinib-treated group versus 27.7 months for the chemotherapy-treated group [79]. Overall, the study strengthened the rationale of daily administration of ALK inhibitors, in particular crizotinib [79].

Despite the described benefits, a number of LC patients treated with crizotinib developed resistance or relapsed within the following 12–24 months [80]. The resistance to crizotinib is mediated by the onset of secondary mutations which may occur within the TK domain of the ALK gene [80,81]. Several ALK point mutations have been identified and include L1152R, C1156Y, I1171T, L1196M, G1202R, S1206Y, V1180L, and G1269A [82,83]. All these mutations reduce crizotinib affinity for the ALK fusion protein through different mechanisms (such as mutations in the ATP binding site or in the solvent-exposed region) [84]. 

Alectinib, ceritinib, and brigatinib are second-generation ALK selective inhibitors, often used after crizotinib in case of resistance or relapse [85].

The efficacy of alectinib versus crizotinib was evaluated in the J-ALEX study, a randomized phase III study whose first results were published in 2017 [86]. At the first data cutoff, 24 patients in the alectinib group discontinued the treatment compared with 61 in the crizotinib group, either due to lack of efficacy or presence of TRAEs. Grade 3 or 4 adverse events occurred at a greater frequency with crizotinib (52%) than with alectinib (26%). No TRAEs with a fatal outcome occurred [86]. 

The median follow-up after 42 months demonstrated that alectinib induces better results than crizotinib in ALK-inhibitor-naïve ALK-positive NSCLC patients, with a favorable safety profile (with incidences of TRAEs of 13.6% and 25.0%, respectively). The median PFS was 34.1 months in the alectinib arm compared with 10.2 months of the crizotinib group [87].

A matching-adjusted indirect comparison (MAIC) was conducted in order to evaluate the relative efficacy of brigatinib, ceritinib, and alectinib in five clinical trials administering one of these second-generation ALK inhibitors. The data analyses were conducted at patient-level by analyzing the ALTA trial for brigatinib; ASCEND-1 and ASCEND-2 for ceritinib; and NP28761 and NP28673 for alectinib. ORR, PFS, and OS were compared [88]. Overall, the analysis suggested that brigatinib may have prolonged PFS and OS versus ceritinib and prolonged PFS versus alectinib, whereas no statistically significant differences have been observed for the ORR [88]. In addition, brigatinib also showed a higher intracranial objective response rate than crizotinib in naïve NSCLC patients with any brain metastases at baseline (79% versus 23%, respectively) [89].

Notably, some ALK mutations, e.g., the ALK G1202R mutation, induce resistance to first- and second-generation inhibitors. Therefore, lorlatinib ALK inhibitors were recently developed to treat ALK G1202R-mutated patients [90,91]. 

In 2018, a clinical study revealed an ORR of 90.0% and an intracranial ORR of 66.7% in naïve NSCLC patients treated with lorlatinib, whereas 198 patients previously treated with ALK inhibitors showed an ORR of 47.0% and an intracranial ORR of 63.0%. Overall, the study demonstrated the anticancer activity of lorlatinib in all the types of ALK-positive NSCLC patients, both naïve and progressed upon TKI therapy [92].

New results from the same study, published in 2021, explored exposure–response relationships in all enrolled patients (328 in total). The obtained results helped to further refine the dose to be administered in order to have the maximal efficacy and the minimal TRAE appearance [93].

The first results from the CROWN phase III study were recently published. The study tested both the efficacy and safety of lorlatinib compared to crizotinib administered to advanced ALK-positive NSCLC patients who were treatment-naïve [94]. The patients treated with lorlatinib showed a PFS at 12 months of 78% compared to the 39% of crizotinib-treated patients [94]. The lorlatinib-treated arm showed an ORR of 76% compared with the 58% registered in the crizotinib-treated arm. Pivotally, 71% of the patients with measurable brain metastases who received lorlatinib had an intracranial complete response. Regarding the toxicity, 34% of lorlatinib-treated patients developed severe TRAEs compared with 27% of the crizotinib-treated patients. The higher TRAEs might be related to the frequent occurrence of altered lipid levels in lorlatinib-treated subjects [94]. Overall, lorlatinib showed a good efficacy. However, the limiting factors to lorlatinib administration are related to several adverse effects such as diarrhea, hyperlipidemia, edema, peripheral neuropathy, and central nervous system disorders including cognitive disorders, amnesia, disorientation, and delirium [94].

The findings hereby reported concerning the clinical use of ALK-TKI as standard practice to tackle NSCLC evidenced some major points. First-, second-, and, especially, third-generation molecules were shown to be effective in increasing the survival rate of advanced NSCLC patients with ALK gene alterations. In particular, lorlatinib has been proven effective in reducing brain metastases. The remaining issue is due to the unsolved problem of new ALK mutations arising upon treatment, which may lead to tumor recurrence and therapy resistance. New ALK inhibitors are currently under clinical evaluation (Supplementary Table S1). In the future, the introduction of gene-sequencing methodologies might help to select the best patient-tailored therapy [95].

### 2.3. B-Raf Proto-Oncogene

The human BRAF gene is located in chromosome 7. It encodes a protein serine/threonine kinase belonging to the RAF family involved in the Ras/PI3K/Akt/mTOR and the RAS-RAF-MEK-ERK signal translation cascade, better known as the Mitogen Activated Protein Kinase (MAPK) pathway (Figure 1). When induced under physiological conditions, this pathway promotes a cascade of phosphorylation of different downstream kinases which are involved in the regulation of cell proliferation, differentiation, and survival [96,97]. 

Oncogenic BRAF mutations were found in different types of human cancers, including melanoma, colorectal cancer, papillary thyroid carcinomas, ovarian cancer, and NSCLC [98,99,100,101,102,103]. The most commonly identified mutation is the BRAF V600E. This amino acid substitution eliminates a key protein–protein interaction which physiologically occurs between the activation segment and the P cycle of glycine [104]. Additional recurrent point mutations found in NSCLC patients include the activating substitutions G469X, K601E, and L597X and the inactivating substitutions D594X, T599I, and G466X [96,105]. NSCLC patients carrying the V600E mutation have a worse PFS and OS, as well as a lower response to platinum-based chemotherapy compared with patients with wild-type BRAF. Instead, the predictive value of BRAF mutations regarding targeted agents is still under study [106]. 

Recent findings evidenced that lung ADC patients carrying the V600E BRAF mutation had a better prognosis compared with patients carrying different BRAF mutations [107]. Contrariwise, other studies found the opposite [108]. Based on the efficacy of BRAF inhibitors in melanoma patients, the same drugs approved for BRAF-mutated melanoma treatment were administered to BRAF-mutated NSCLC patients. Currently, dabrafenib (Tafinlar, FDA approved) and vemurafenib (Zelboraf, under clinical investigation with regard to LC) are the two drugs administered for the treatment of BRAF V600E-mutated NSCLC patients with metastatic disease [109]. These drugs are reversible ATP competitors of the BRAF kinase domain, thus able to reduce the downstream MAPK activation [109].

In particular, vemurafenib activity was investigated in a multicenter phase II study, whose first results were published in 2015. The trial explored both the safety and efficacy of vemurafenib administered to 122 non-melanoma patients, including 22 NSCLC patients, with the V600 BRAF mutation [110]. Overall, the patients showed an ORR of 42% with a median PFS of 7.3 months. Regarding the toxicity, 16 NSCLC patients (corresponding to 80%) developed grade 3–4 TRAEs. Overall, the findings demonstrated that vemurafenib might be effective in non-melanoma cancers carrying the BRAF V600E mutation, including NSCLC [110]. 

AcSé is an ongoing phase II study, whose first results on NSCLC patients with mutated BRAF were recently published in 2020 [111]. The study assessed both the safety and efficacy of vemurafenib monotherapy in cancer patients carrying various BRAF mutations [111]. 

The BRAF V600-positive group showed an ORR of 44.8% with a median DOR of 6.4 months. The median PFS and the median OS were 5.2 and 10 months, respectively. On the contrary, no benefit was reported for patients presenting non-V600 BRAF mutations. With regard to toxicity, severe TRAEs were developed by 36% of the BRAF V600-positive patients compared with 27% of the BRAF non-V600 group [111].

In a phase II, multicenter, non-randomized, open-label study (BRF113928), a total of 84 previously treated (N. 78) and untreated (N. 6) patients with stage IV metastatic BRAF V600E-positive NSCLC were enrolled to test the efficacy of dabrafenib [106]. The patients showed an ORR of 33% with a DCR of 58% and a median DOR of 9.6 months. Measured median PFS and median OS were 5.5 and 12.7 months, respectively. Regarding toxicity, 35 patients out of 84 developed severe TRAEs related to the treatment, including the development of cutaneous squamous cell carcinoma, asthenia, and basal-cell carcinoma. Overall, the study suggested that dabrafenib might represent a valid therapeutic option for BRAF V600E-positive NSCLC patients [106]. 

Several clinical trials demonstrated that the combination of dabrafenib with the MEK inhibitor trametinib (Mekinist) had a higher ORR, more durable responses, and improved tolerability and toxicity profiles in comparison with chemotherapy in both first- and second-line treatments of NSCLC [112]. 

The results of the BRF113928 trial demonstrated that the combination therapy had a significant antitumor activity and a better safety profile [113]. Later observations further confirmed that a longer TTP duration following dabrafenib monotherapy or combination therapy was associated with a significantly longer PPS duration in patients with BRAF V600E-mutant NSCLC [114].

However, despite the good outcomes, the response to these BRAF inhibitors may not be prolonged due to the development of drug resistance in about 70% of the patients carrying the BRAF V600E mutation [106,115]. The combination of dabrafenib with trametinib (anti-MEK inhibitor) did not prevent the development of BRAF-inhibitor resistance. To overcome such resistance, a third generation of BRAF inhibitors is currently being investigated under preclinical study. These agents, better known as pan-RAF, demonstrated the capability to induce the cleavage of the enzyme poly (ADP-ribose) polymerase and to inhibit both BRAF monomers and dimers with subsequent anticancer effects. The efficacy of these molecules has been proven preclinically in both melanoma and NSCLC models with marked anti-proliferative responses [116,117]. Additionally, a number of clinical trials assessing the efficacy of combining BRAF inhibitors with other TKIs are currently ongoing (Supplementary Table S1).

### 2.4. Rearrangement during Transfection

The human Rearrangement during Transfection (RET) proto-oncogene is located in chromosome 10, and it encodes for a protein receptor tyrosine kinase. This receptor is involved in the physiological development of the nervous system, as well as in guiding the shaping of the organs originating from the neural crest, especially the kidneys [118]. The activation of RET led to the downstream activation of different signaling pathways, including the RAF/MEK/ERK, PI3K/AKT/mTOR, JAK/STAT, and c-Jun N-terminal kinase (JNK) (Figure 1) [118].

The most common RET gene alterations are inversions generating different fusion genes. These fusion genes may be associated with the onset of several cancers, including LC. RET gene fusion has been identified in 1–2% of young (<60 years) non-smoker lung ADC patients, and these rearrangements are also correlated with the development of brain metastases [119,120]. The first oncogenic RET alteration discovered in LC is the RET-KIF5B gene fusion. Currently, several RET fusion partners have been identified, including CCDC6, CLIP1, ELE1, ERC1, EPHA5, FRMD4A, KIAA1217, MYO5C, NCOA4, PICALM, RUFY2, TRIM24, and TRIM33 [121]. The chimeric proteins result in a ligand-independent activation of the RET tyrosine kinase domain [122]. 

Different multitarget kinase inhibitors (MKIs, including alectinib, cabozantinib, lenvatinib, ponatinib, regorafenib, sorafenib, sunitinib, and vandetanib) have been evaluated in LC patients with RET rearrangement. However, these drugs displayed high toxicity and limited therapeutic benefits [123,124,125,126]. In 2020, the FDA approved two selective RET inhibitors (selpercatinib and pralsetinib) for the treatment of advanced LC patients with RET rearrangements [127,128]. The X-ray crystal structures of pralsetinib and selpercatinib showed that these compounds are very similar. In particular, they showed the occurrence of a hydrogen bond between the amino group of the compound and the carbonyl group of the residue A807 of RET protein [129].

The LIBRETTO-001 phase I/II study currently aims to assess the clinical safety and efficacy of selpercatinib in patients with advanced RET fusion-positive solid tumors, including NSCLC [130]. The arm composed of previously treated patients showed an ORR of 70% with a median DOR of 20.3 months and a median PFS of 18.4 months. In contrast, the naïve group showed an ORR of 90%, whereas the DOR and PFS cutoff had not been reached. Importantly, among patients with measurable brain metastases, 91% showed an objective intracranial response [130]. Regarding the toxicity, only 6% of the selpercatinib-treated patients developed grade 5 TRAEs, whereas 14% developed grade 3 or 4 TRAEs. Overall, the study proved that selpercatinib is effective in patients with RET fusion-positive NSCLC, with mainly low-grade TRAEs [130].

In 2021, the first results from the ARROW study were published [131]. ARROW is an ongoing multicenter phase I/II trial which aims to assess both the safety and efficacy of pralsetinib, a potent and oral selective RET inhibitor, in patients with RET fusion-positive solid tumors, including NSCLC [131]. 

The 87 pretreated patients showed an ORR of 61% compared with an ORR of 70% in the naïve patients. The measured median PFSs were 17.1 and 9.1 months in the pretreated and naïve groups, respectively. Regarding the toxicity, 93% of the entire cohort developed TRAEs, but serious TRAEs were only registered for 24% of the cohort, including pneumonia, pneumonitis, anemia, and neutropenia. Importantly, a significant tumor shrinkage was observed in 95% of the pretreated patients and in 100% of the naïve patients having a measurable disease. Furthermore, in all patients with measurable intracranial metastases, a significant reduction of the tumor mass was registered [131].

Hypertension, increased AST and ALT levels, hyponatremia, and neutropenia were the most common TRAEs observed. Only a reduced number of NSCLC patients discontinued the treatment due to toxicity [132]. These data suggest that both of the RET inhibitors displayed high efficacy and tolerable toxicity and may be used for the treatment of naïve and recurrent RET-positive NSCLC.

### 2.5. Hepatocyte Growth Factor Receptor

The human hepatocyte growth factor receptor (HGFR) gene, also known as c-Met or MET, is located in chromosome 7. It consists of 21 exons encoding for a heterodimer tyrosine kinase receptor [133,134]. The binding between HGFR and its ligand induces the activation of several downstream signaling pathways, including the MAPK, JAK/STAT, WNT/β-catenin, and PI3K/AKT pathways (Figure 1) [135]. Normal MET gene expression levels play an important role during embryogenesis and, in adults, during tissue damage responses [136]. 

The most common alterations of the MET gene include amplifications, point mutations, and rearrangement, all leading to HGFR receptor overexpression and consequent cellular promotion of epithelial-to-mesenchymal transition (EMT), invasion, and metastatization [137]. In particular, MET exon 14 skipping mutations are present in 2–4% of patients with NSCLC [138,139]. 

MET mutations lead to the loss of a region of juxta-membrane domain, the resulting protein cannot be recognized by ubiquitins, thus escaping proteasomal degradation [139]. Generally, MET gene overexpression is normally associated with a poor LC prognosis [140,141]. 

Several compounds targeting the mutated HGFR protein or its ligand HGF have been developed and tested. Such molecules can be classified into non-selective multitarget TKIs, selective TKIs, and mAbs [142]. TKIs can be divided into three main types. Type I TKIs bind to the ATP binding site in its active form, and may be subclassified into two different subgroups. Type Ia inhibitors are less specific for the HGFR protein, probably because they interact more with the G1163 residue, which has an analog in both ALK and ROS1 proteins, whereas type Ib inhibitors interact more specifically with the Y1230 residue but not with the G1163 residue. Type II inhibitors bind to the ATP pocket in its inactive state without any interaction with the G1163 residue, but instead with the ATP hydrophobic back site. Type III inhibitors bind to the allosteric sites or the receptor with no interaction with the ATP binding site [142].

MET exon 14 skipping mutations are very sensitive to different MET-TKIs. In particular, crizotinib is a type Ia inhibitor, which was approved by the FDA for the treatment of ALK and ROS rearrangements in metastatic NSCLC. Furthermore, this drug demonstrated antitumor activity in NSCLC patients with MET rearrangements [143,144]. For this reason, crizotinib was the first drug approved by the FDA in 2018 for the treatment of advanced NSCLC in MET exon 14 skipping alterations-positive NSCLC patients or with tumor recurrence after first-line platinum-based chemotherapy [145]. 

PROFILE-1001 is a phase I study designed to assess both the safety and efficacy of crizotinib in NSCLC patients with MET exon 14 alterations or ALK or ROS1 alterations. Regarding the MET-mutated cohort, the first results were published in 2020 [146]. Upon continuous administration of critozinib with escalating dosage, the patients showed a median OS of 20.5 months, a DOR of 9.1 months, and a median PFS of 7.3 months. The ORR was 32% (in 21 patients out of 65). In total, 5% of the patients showed a complete response, whereas 27% showed only a partial response. The response duration was ≥6 months. Regarding toxicity, the most common TRAEs observed were grade 1 or 2 and included edema, vision disorders, nausea, and diarrhea. Overall, the study emphasized the efficacy of administering crizotinib to NSCLC patients with occurring MET exon 14 alterations [146].

More recently, the testing of novel selective MET TKIs generated promising results. MET TKIs include type Ib capmatinib (Tabrecta) and tepotinib (Tepmetko). Both drugs have been approved by the FDA for the treatment of treatment naïve and previously treated metastatic MET exon 14 skipping-altered NSCLC: capmatinib in 2020 and tepotinib in 2021 [147,148].

GEOMETRY MONO-1 is a phase II study, whose first outcomes were published in 2020. The patients enrolled were EGFR wild-type NSCLC patients. In particular, the safety, efficacy, and pharmacokinetics of tapmatinib were evaluated in patients with advanced NSCLC with MET amplification or MET exon 14 skipping mutations [149]. 

The 69 patients who had previously received one or two lines of therapy showed an ORR of 41% with a DOR of 9.7 months and a median PFS of 5.4 months, whereas the 28 treatment-naïve patients showed an ORR of 68% with a response duration of 12.4 months and a median PFS of 9.69 months. In patients with MET amplification, the efficacy of the drug was higher with high copy number amplification compared to low copy number [149]. Regarding the toxicity, the most reported TRAEs were grade 1 or 2 events (such as edema, nausea, vomiting, and high creatinine level). Overall, the results support the efficacy of Capmatinib in NSCLC patients with MET exon 14 skipping alterations, especially in treatment-naïve patients [149].

Tepotinib safety and efficacy were explored in the VISION clinical trial, whose first results were published in 2020 [150]. The patients enrolled presented advanced NSCLC with MET exon 14 skipping alterations or MET amplification. The 99 eligible patients (43 treatment-naïve and 56 previously treated with other therapies, including immunotherapy) showed an ORR of 46%, and all of them showed a partial response [150]. The median DOR, median PFS, and median OS registered were 11.1, 8.5, and 17.1 months, respectively. Regarding the toxicity, 89% of the patients reported mild TRAEs. In total, 15% of patients developed severe TRAEs, such as peripheral edema. Overall, the study strengthened the rationale of routinely using tepotinib in patients with NSCLC with occurring MET exon 14 skipping mutations [150]. 

The MET exon 14 skipping mutation characterizes about 2–4% of NSCLC cases, and it represents an important therapeutic target. The currently approved selective drugs showed significant efficacy and manageable toxicity, and future studies will assess the improved efficacy in combination with other TKIs (Supplementary Table S1).

### 2.6. Neurotrophic Tropomyosin Receptor Tyrosine Kinases

The neurotrophic tropomyosin receptor kinase (NTRK) gene family includes NTRK1, NTRK2, and NTRK3 genes. They encode three transmembrane tyrosine kinases TRKA, TRKB, and TRKC, which play a key role in neuronal development and cell differentiation [151]. Each receptor binds to a specific ligand; in particular, TRKA, TRKB, and TRKC show the highest affinities for brain-derived growth factor (BDGF), neurotrophin-4, and neurotrophin-3, respectively [152]. 

The binding between a TRK receptor and its ligand activate downstream pathways, such as MAPK, PI3K, and phosphoinositide phospholipase Cɣ (PLCɣ) [151]. The chromosomal rearrangements of the NTRK genes with several fusion partners, including CD74, ETV6, LMNA, MPRIP, SQSTM1, and TRIM24, have been reported in different cancers, including large-cell neuroendocrine carcinoma and NSCLC, and result in the constitutive activation of the kinase domain [153,154].

The oncogenic activity of the TRK chimeric proteins is efficiently blocked by different TRK inhibitors, including larotrectinib and entrectinib, which have shown remarkable efficacy in the treatment of advanced solid tumors [155]. In particular, larotrectinib is a selective TRKA, TRKB, and TRKC inhibitor that showed clinical benefits in several TRK rearrangement-positive cancers. Alternatively, entrectinib is a multi-kinase inhibitor, previously approved by the FDA for the treatment of patients with ROS1 and ALK rearrangements [155].

Larotrectinib’s anticancer activity was studied in three different clinical trials, and their outcomes were published in 2018 [156]. The studies aimed to uncover both the safety and efficacy of larotrectinib when administered to adults, adolescents, and children with solid tumors harboring NTRK gene fusions. All 55 patients enrolled showed an ORR of 75% and a DCR of 80%, whereas the median DOR and PFS had not been reached. Regarding toxicity, reported TRAEs were rare and the most common were grade 1 or 2 [156]. 

More recent results from the contributing trials were published in 2020 [157]. A cohort of 159 patients (12 with NSCLC) showed an ORR of 79% (16% with a complete response and 63% with a partial response). In addition, the study showed more benefits in terms of DOR, median OS, and median PFS, which were 35.2, 44.4, and 28.3 months, respectively. Regarding the toxicity, no new TRAEs were reported compared to the previous report [157,158]. 

The datasets from three different phase I/II clinical studies, ALKA-372-001, STARTRK-1, and STARTRK-2, were analyzed in 2020. The studies evaluated both the safety and efficacy of orally administering entrectinib to adults with locally advanced metastatic cancer with NTRK1, NTRK2, NTRK3, ROS1, or ALK rearrangements [159]. The 55 patients enrolled (10 with NSCLC) showed an ORR of 57.4% (70% in patients with NSCLC), a median OS of 20.9 months, and a median PFS of 11.2 months (14.9 months in patients with NSCLC). With regard to toxicity, the most frequent TRAEs developed were mild and manageable grade 1 or 2 events. Overall, these data demonstrated the low toxicity and the high efficacy of entrectinib for the treatment of NTRK fusion-positive patients [159]. As a consequence, entrectinib was approved by the FDA for the treatment of patients with metastatic or advanced solid tumors bearing the NTRK gene fusion [155]. 

Currently, several NTRK point mutations conferring resistance to larotrectinib or entractinib have been identified. These mutations mainly occur within the catalytic region of the NTRK kinase domain, thus impairing the effective binding of the TKIs. The most common NTRK mutations include G595R, F589L and G667S in NTRK1, G623E and G623R in NRTK2, and F617L and G696A in NTRK3 [160]. To counteract such resistance, several second-generation NRTK inhibitors have been developed and tested. Selirectinib, repotrectinib, and taletrectinib are among the investigational drugs currently under clinical evaluation as effective TRK inhibitors to counter resistance to first-generation NTRK inhibitors in cancer (Supplementary Table S1) [161].

### 2.7. Kirsten Rat Sarcoma Viral Oncogene Homolog

The human Kirsten rat sarcoma viral oncogene homolog (KRAS) is an oncogene located in chromosome 12. The gene encodes for a GTPase membrane-bound protein, which is involved in the activation of different signaling pathways. These pathways, which include the RAF/MEK/ERK, PI3K/AKT/mTOR, and RALGDS/RAL/FLIP, regulate several cellular processes, including proliferation, survival, differentiation, and cytoskeletal reorganization (Figure 1) [162,163,164]. 

Oncogenic KRAS mutations have been found in a variety of cancers, including LC, colon cancer, and pancreatic cancer [165,166,167]. In particular, KRAS mutations are found in 20–40% of lung ADC patients affecting codons 12, 13, and 61 of the KRAS gene [168]. Such mutations determine the impairment of the GTP hydrolysis, leading to the overactivation of the downstream signaling pathways, and hence, to uncontrolled cellular proliferation [169]. The most frequent KRAS mutations are G12D, G12C, and G12V. Cancer cells with G12C or G12V KRAS show high levels of kinase activity and low levels of phosphorylated receptors compared with wild-type cell lines. The specific PI3K-AKT pathway activation is mainly associated with the KRAS G12D subtype and other co-mutations affecting TP53, STK11, KEAP1, and CDKN2A/B [170,171,172]. 

Several small molecules were evaluated, including farnesyltransferase inhibitors (tipifarnib, lonafarnib, and salirasib) and other downstream inhibitors (sorafenib, selumetinib, and abemaciclib); however, conflicting results were obtained [173].

Adagrasib (MRTX849) and sotorasib (AMG 510) are small molecules able to bind the KRAS G12C-mutated protein. The first preclinical studies demonstrated their consistent safety profile and their moderate antitumor activity in different advanced KRAS G12C-mutant solid tumor models, including LC [174]. Furthermore, clinical evaluation supported their approval (May–June 2021) by the FDA for the treatment of NSCLC cancer patients with the KRAS G12C mutation [175,176].

The KRYSTAL-1 multi-cohort phase I/II study assesses the safety, pharmacokinetics, and efficacy of adagrasib administered to patients with KRAS G12C-mutated advanced tumors [177]. Of the 79 patients, only 51 were evaluable for clinical activity. They showed an ORR and a DCR of 45% and 96%, respectively. Regarding toxicity, about 30% of the cohort developed mild TRAEs including nausea, diarrhea, vomiting, fatigue, and increased ALT. Hyponatremia was reported as the only severe TRAE (in 3% of the patients). Overall, the study had successfully proven the tolerability and efficacy of adagrasib in pretreated NSCLC patients harboring such mutation [177].

Similarly, CodeBreaK 100 is a phase I/II study to evaluate the tolerability and efficacy of sotorasib in patients with advanced solid tumors harboring the KRAS G12C mutation. The first results were published in 2020 and analyzed the outcomes for a total of 129 patients (59 with NSCLC) [178]. The second analysis, published in 2021, reported the results specifically for 126 enrolled patients (from 2019 to 2020) with NSCLC, mainly previously treated either with platinum-based therapy or with anti-PD1/anti-PD-L1 antibodies [176]. Among the 124 patients evaluated for response, 34% had a partial response, whereas 3% had a complete response. The DC occurred in 82.3% of patients and the ORR in 37.1%. In the patients who showed a significant ORR, the time of response was 1.4 months. TRAEs were observed in 99.2% of the patients; 42% reported grade 3 events and 3% reported grade 4 events. Overall, sotorasib showed clinical efficacy with reversible toxic effects, further supporting its clinical use for patients with KRAS G12C-mutated NSCLC [176].

Ongoing studies are exploring novel molecules to overcome the issues of drug resistance and recurrence in KRAS-positive NSCLC (Supplementary Table S1).

### 2.8. Proto-Oncogene Tyrosine-Protein Kinase ROS

The human c-Ros proto-oncogene 1 (also known as ROS1) is located in chromosome 6, and it is a member of the tyrosine kinase insulin receptor gene family. ROS1 encodes for a type I integral membrane protein with tyrosine kinase activity. ROS1 receptor activation allows the downstream triggering of several intracellular signaling pathways, including PI3K/AKT/mTOR, MAPK, and JAK/STAT [179]. The activation of such pathways positively modulates cell growth and differentiation (Figure 1). ROS1 gene rearrangements lead to the overactivation of the downstream pathways, with important pro-tumoral effects. In particular, ROS1 rearrangements were identified in 1–2% of patients with NSCLC, as well as in different types of cancer [180,181]. 

Different ROS1 rearrangements have been identified, which determine the production of chimeric proteins with different fusion partners. The most common gene partners include CD74, EZR, SLC34A2, TPM3, and SDC4 [182]. ROS1-mutated NSCLC tends to be very aggressive and to quickly metastasize, but at the same time exhibits an unprecedented response to ALK inhibitors [179]. Indeed, ROS1 tyrosine kinase domain has a 49% homology with the ALK tyrosine kinase domain. Overall, ROS1 has 77% of its amino acids in common with the ALK protein. For this reason, some ALK inhibitors are also effective in patients with ROS1 rearrangement [183]. 

The abovementioned PROFILE-1001 phase I study also enrolled a cohort of NSCLC patients with ROS1 gene rearrangements [184]. The study evaluated the efficacy and safety of crizotinib. The 50 patients enrolled showed an ORR of 72% with a median DOR of 17.6 months. The median PFS was 19.2 months without any recorded difference in response to crizotinib between patients with different types of ROS1 rearrangement. Regarding the toxicity, 10% of the patients with ROS1 rearrangements developed TRAEs similar to those recorded in the group of NSCLC patients with ALK rearrangement. Overall, the study demonstrated the anticancer activity as well as the manageable toxicity of crizotinib administered to NSCLC patients with occurring ROS1 rearrangements [184].

The AcSé study aimed to evaluate the effects of crizotinib in BRAF-, ROS1-, and MET-positive NSCLC [185]. The 25 patients with MET amplification showed an ORR of 16% with a DCR of 52%, a median PFS of 3.2 months, and a median OS of 7.7 months. The 28 patients with MET mutation showed an ORR of 10.7% with a DCR of 39%, a median PFS of 2.4 months, and a median OS of 8.1 months. The 37 patients with an ROS1 translocation showed an ORR of 47.2% with a DCR of 69.4%, a median PFS of 5.5 months, and a median OS of 17.2 months. With regard to toxicity, the patients developed mostly low-grade TRAEs. The findings proved both the efficacy and tolerability of crizotinib in MET- and ROS1-positive patients [185].

Two additional TKIs are currently used in ROS1-positive NSCLC: entrectinib (Rozlytrek) and ceritinib (Zykadia). However, ceritinib is not currently approved by the FDA for patients with ROS1-positive NSCLC, but it is under investigation. In fact, a small open-label phase II study aimed to assess the efficacy and associated toxicity of administering ceritinib in 32 patients with advanced NSCLC harboring the ROS1 rearrangement, 28 of which were evaluable for response [186]. The results, published in 2017, showed an ORR of 62% with a DCR of 81%. The median PFS and OS were 9.3 and 24 months, respectively. A decrease in tumor burden from the baseline was observed in 75% of the patients. The two patients with brain metastases showed a significant shrinkage of the lesions after four weeks on ceritinib. Regarding toxicity, 16 patients (50%) developed serious TRAEs, but only 22% of such events were considered related to treatment, and only one patient discontinued the treatment as a result of the TRAEs [186].

As reported above, the ALKA-372-001, STARTRK-1, and STARTRK-2 studies evaluated both the safety and efficacy of orally administering entrectinib to adult patients with locally advanced metastatic cancer with NTRK1, NTRK2, NTRK3, ROS1, or ALK rearrangements. A total of 41 out of 53 ROS1-positive NSCLC patients were efficacy-evaluable (23 with baseline brain disease and 30 without baseline brain disease) [187]. 

The 30 patients without baseline brain metastases showed an ORR of 80% with a median DOR of 24.6 months. The median PFS was 26.3 months. The group of 23 patients with brain metastases showed an ORR of 74% with a median DOR of 12.6 months. For this last group, the median PFS was 13.6 months, and the intracranial response was 55%. Regarding toxicity, grade 3 TRAEs occurred in 31% of the patients, and grade 4 TRAEs occurred in 4% of them. Overall, the study highlighted the good systemic and intracranial activity of entrectinib in the studied cohort of patients. Additionally, the safety profile was favorable. Thus, entrectinib can be suggested for use in NSCLC patients with ROS1 rearrangements at an advanced stage. Finally, routine testing for the presence of ROS1 gene fusions in NSCLC patients might be suggested as a clinical routine [187]. 

Upon treatment with crizotinib, CD74-ROS1 fusion protein-positive patients may be resistant. The resistance might be due to the acquisition of the G2032R point mutation, located in the ROS1 kinase domain, and overall hindering of the binding of crizotinib. Furthermore, the mutated residue causes steric clashes with the piperidine ring of crizotinib, consequently causing the failure of the treatment [188,189]. 

Lorlatinib (Lorbrena) and cabozantinib (Cometriq) are the inhibitors currently being studied to overcome crizotinib resistance [190,191]. Lorlatinib revealed a strong antitumor activity also in the presence of other ROS1 point mutations including L2026M, D2033N, and S1986, known to induce the development of crizotinib-resistance [192].

In recent years, a selective ROS1 inhibitor, taletrectinib (DS-6051b/AB-106), was tested to overcome crizotinib resistance, showing survival benefits in clinics. The efficacy of taletrectinib was evaluated in two clinical studies, whose results were published recently in 2021 [193]. A total of 61 ROS1-positive advanced NSCLC patients were enrolled in two studies: the Japanese J102 and the American U101. The patients were divided into two groups (treatment-naïve and previously treated with crizotinib). Treatment-naïve patients showed an ORR of 66.7% with a DCR of 100% and a median PFS of 29.1 months. Patients that were refractory to crizotinib showed an ORR of 33.3% with a DCR of 88.3% and a median PFS of 14.2 months. The most common TRAEs were ALT increase (18%), AST increase (9%), and diarrhea (4.5%). The study has proven the high and durable response of taletrectinib in both groups, with manageable TRAEs [193]. 

Novel molecules to overcome crizotinib resistance are currently under clinical assessment (Supplementary Table S1). Overall, given the percentage of ROS1-positive NSCLC patients, and supported by the positive clinical results obtained for several ROS1 inhibitors, ROS1 testing should be routinely used during the anamnestic process of NSCLC, both naïve and treatment-resistant.

### 2.9. Phosphoinositide 3-Kinase

The human Phosphoinositide 3-kinase (PIK3CA) gene is located in chromosome 3, and it encodes for the so-called p100-alpha protein, which is a catalytic subunit of phosphoinositide 3-kinase (PI3K) [194]. PI3K is a protein complex formed by a heterodimeric group of lipid kinases and consisting of a number of catalytic and regulatory subunits. PI3K regulates AKT gene expression, allowing the activation of the PI3K/AKT/mTOR pathway involved in several cellular activities, including cell growth, differentiation, and survival (Figure 1) [195]. 

PIK3CA mutations have been identified in several human tumors, including LC. PIK3CA mutations occur in 2–7% of NSCLC patients, in particular in SqCC [196]. The most commonly detected PIK3CA point mutations are located in the exon 9 (i.e., E545K or E542K) or in the exon 20 (i.e., H1047R or H1047L), which encode for the helical and the kinase domains, respectively. These mutations determine the overactivation of PI3K [196,197]. 

PIK3CA mutations may coexist with other driver mutations in LC, including those involving KRAS and EGFR genes [198]. Recently, several PI3K/AKT/mTOR pathway inhibitors have been developed towards PI3K, AKT, and mTOR. Such drugs are currently tested alone or in combination with chemotherapy.

Buparlisib (BKM120) is a potent, orally bioavailable, pan-class I PI3K inhibitor. The molecule has been tested as a single-agent in a small phase I study, BASALT-1, in 63 NSCLC patients (30 SqCC and 33 non- SqCC) [199]. The results, published in 2015, showed 12-week PFS rates of 23.3% and 20% for the SqCC and non- SqCC cohorts, respectively. Regarding the toxicity, the SqCC patients developed hyperglycemia (23.3%) and fatigue (6.7% each), whereas the non-squamous cancer subjects developed increased ALT (15.2%), hyperglycemia (12.1%), and rash (6.1%). Given the fact that the primary objective was not reached (PFS less than 50%), the phase II trial was not started, and the study was terminated. This finding might suggest that PI3K may not be the main oncogenic driver in NSCLC [199].

The CUSTOM phase II study assessed both the safety and the efficacy of several targeted therapies in thoracic malignancies. The multi-arm study enrolled 647 NSCLC, SCLC, and thymic cancer patients [200]. 

The 16 patients with EGFR mutations, treated with erlotinib monotherapy, showed an ORR of 60%, a median PFS of 11.3 months, and a median OS of 25.7 months. The 11 patients with BRAF, KRAS, and HRAS abnormalities were treated with selumetinib monotherapy and showed an ORR of 11%, a median PFS of 2.3 months, and a median OS of 6.5 months. The 8 patients with ERBB2 mutations or amplifications were treated with lapatinib and showed no significant response. Finally, the 7 patients harboring PIK3CA/AKT/PTEN mutations or amplifications, treated with MK2206, which is a pan-AKT inhibitor, showed no significant response. Overall, the study strengthened the rationale of routinely using a personalized targeted therapy based on the specific mutational profile. However, the design was not feasible for many of the arms with less commonly occurring mutations, including PIK3CA [200].

Taselisib (GDC-0032) is a small molecule which selectively targets the p110α subunit of PI3KA. The phase II SWOG S1400B study aimed to assess both the safety and efficacy of taselisib in stage IV lung SqCC, refractory upon previous treatments and screened positive for PI3K mutations [201]. The patients showed a PFS of 2.9 months and an OS of 5.9 months. Although taselisib demonstrated low toxicity, the study was terminated for futility at interim. Instead, the study was useful for the classification of novel mutations found in PIK3A in lung SqCC [201].

Given the unsuccessful results obtained from these published studies, only one ongoing clinical trial is currently seeking to evaluate the efficacy of a novel PI3K inhibitor (pictilisib) in patients with LC and with occurring alterations in the PIK3CA gene (Supplementary Table S1).

## 3. Immune Checkpoint Inhibitors

Over the last decades, the advent of anticancer immunotherapy has improved both the QoL and the life expectancy of patients, including patients with advanced LC. Modern immunotherapy acts by facilitating the interaction between cancer cells and the immune system by activating specific components of the immune system (e.g., T-lymphocytes, NK cells, macrophages, and checkpoint receptors) [202].

One mechanism of action exploited by immunotherapy is the activation or re-activation of cytotoxic T lymphocytes against cancer cells through the administration of tumor vaccines, cytokines such as interleukin-2, or through the adoptive transfer of tumor-infiltrating T cells (TILs) [203,204]. In the last decade, several strategies have been developed to improve the presentation of specific tumor antigens to antigen presenting cells (APC), such as dendritic cells (DCs) [205]. Another approach consists of triggering the innate immunity and inflammation within the tumor microenvironment with the administration of pro-inflammatory agents, such as interferons (IFNs) and Toll-like receptors (TLRs) [206]. More recently, the better understanding of the immune-suppressive mechanisms of the T cell receptor led to the development of agents, i.e., ICIs, capable of modulating the so-called “immune-checkpoints” and to repristinate the immune system in its fight against the cancer cells [207].

The primary role of the immune checkpoint signaling is to protect the tissues from damage when the immune system responds to pathogens and, more generally, to maintain the tolerance to self-antigens [29]. An increasing amount of evidence shows that a primary mechanism by which tumors escape the immune system is the activation of such checkpoints [29]. 

**Figure 2:**
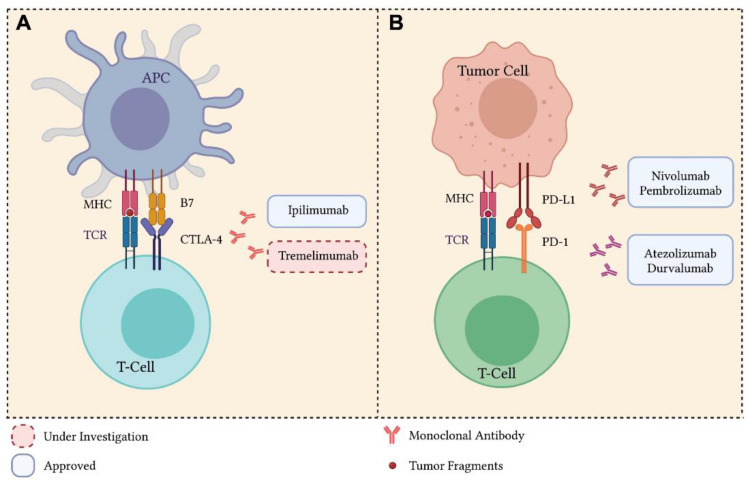
Immune checkpoint inhibitors for the treatment of lung cancer. (**A**) Molecular targets of anti-CTLA-4 mAbs ipilimumab and tremelimumab. (**B**) Molecular targets of anti-PD-1 and anti-PD-L1 mAbs nivolumab and pembrolizumab (anti-PD-1), atezolizumab, and durvalumab (anti-PD-L1). Blue boxes indicate FDA-approved drugs, and red dotted boxes indicate drugs under investigation.

The last ten years have witnessed the development of ICIs as antibodies capable of blocking such checkpoints. As described in the Introduction section, this blockage, which can happen either in the earlier stage of antigen presentation (CTLA4-B7) or later at the tumor site (PD-1/PL-L1), efficiently reactivates the T cells to recognize and eliminate cancer cells (Figure 2) [208]. Whereas CTLA-4 mainly regulates the activation of T cells in the lymphatic tissues, the main role of PD-1 is to limit the activity of T cells in the peripheral tissues [209]. The PD-L1 ligand is commonly upregulated on several human solid tumors, including melanoma, ovarian tumors, and NSCLC [210,211]. Currently, several anti-CTLA4, anti-PD-1, and anti-PD-L1 antibodies (including nivolumab, pembrolizumab, atezolizumab, and durvalumab) have been approved for the treatment of solid and hematological tumors [212]. One issue with the administration of ICIs is the development of the so-called immune-related adverse events (irAEs), which can vary from mild to severe [213]. This can significantly affect the therapeutic efficacy of ICIs, as well as the overall compliance of cancer patients.

In 2015, the anti-PD-1 mAb nivolumab was approved by the FDA as a second-line treatment for patients with advanced NSCLC. Subsequently, several ICIs were subjected to clinical studies and finally obtained approval as second- and first-line LC treatments [214,215]. Currently, ICI-based therapy is a standard of care for patients affected by locally advanced and metastatic NSCLC without EGFR/ALK alterations, as well as advanced SCLC. ICIs are administered either as monotherapy or as combined therapy (with other ICIs, targeted therapies, or chemotherapy). Additionally, novel clinical trials are currently ongoing to assess the efficacy of ICIs as adjuvant or neo-adjuvant therapy in early-stage LC [212].

Despite the cutting-edge advancement achieved, ICI efficacy faces several challenges, including LC intrinsic heterogeneity, the genetic mutational landscape, and the individual immune system reactivity [216]. In particular, the clinical benefits of ICIs are available only for a limited cohort of cancer patients. Additionally, as for the targeted therapy, several ICI-treated patients might develop resistance and relapse [217]. As mentioned, responder LC patients can develop irAEs, from mild to severe, including thyroiditis, hepatitis, dermatologic manifestations, colitis, intestinal mucositis, diarrhea, pneumonitis, etc., [218].

The following paragraphs describe the clinical achievements leading to the approval of ICIs in LC alone or in combination with chemotherapy, radiation, other ICIs, or targeted therapies. The combination strategies might represent a robust way of overcoming the issues of resistance and relapse in order to achieve a longer free interval. Given the great clinical results currently achieved and illustrated below, many clinical trials are currently ongoing to evaluate both the safety and efficacy of ICIs alone or in combination with other therapies, including ICIs (Supplementary Table S2) and targeted therapies (Supplementary Table S3). 

### 3.1. Cytotoxic T Lymphocyte Antigen-4 Inhibitors

Cytotoxic T Lymphocyte Antigen-4 (CTLA-4) plays a critical role in maintaining the activation of T cells (Figure 2). Ipilimumab, which blocks the binding of CTLA-4 with its co-receptor B7 expressed by DCs, is the first mAb to receive FDA approval for the treatment of advanced melanoma, with an improved efficacy compared to the glycoprotein 100 peptide vaccine [219]. Tremelimumab, another anti-CTLA-4 mAb, is currently under clinical testing for the treatment of a variety of solid cancers, including LC [220]. As a result of the mediated CTLA4/B7 checkpoint blockage, activated T cells, including those activated by tumor antigens, can continue to proliferate, produce cytokines, and hence, perform their cytotoxic effects within the tumor microenvironment [221].

Based on the positive outcomes observed in patients with melanoma, ipilimumab has been tested in LC. A phase II randomized and double-blind study was designed to assess both the safety and efficacy of ipilimumab in combination with chemotherapy as a first-line treatment in LC patients, both NSCLC and SCLC [222].

The patients treated with ipilimumab showed a median immune-related progression-free survival (irPFS) of 5.7 months compared with 5.5 and 4.6 months for concurrent ipilimumab and placebo groups, respectively. The median OSs were 12.2, 9.7, and 8.3 months for the ipilimumab, concurrent ipilimumab, and placebo groups, respectively. Patients who received the placebo showed an immune-related best overall response rate (irBORR) and a best ORR (BORR) of 18% and 14%, respectively. The irBORR and BORR were both 32% for patients treated with ipilimumab and 21% for the concurrent ipilimumab group. With regard to toxicity, grade 3 or 4 irAEs were developed by 15%, 20%, and 6% of patients enrolled in the ipilimumab, concurrent ipilimumab, and placebo arms, respectively [222].

On the other hand, patients with extensive-disease SCLC (ED-SCLC) treated with ipilimumab showed a median irPFS of 6.4 months compared with 5.7 and 5.3 months for the concurrent ipilimumab and control groups, respectively. The median OSs were 12.9, 9.1, and 9.9 months for the ipilimumab, concurrent ipilimumab, and control groups, respectively. The irBORRs were 71%, 49%, and 53% for the phased ipilimumab, concurrent ipilimumab and control groups, respectively, whereas the BORRs were 57%, 33%, and 49%, respectively. Regarding the toxicity, grade 3 or 4 irAEs were developed by 17%, 21%, and 9% of patients in the ipilimumab, concurrent ipilimumab, and control groups, respectively [223]. Overall, the data obtained in both cohorts strengthened the rationale of routinely using ipilimumab as a first-line treatment in combination with two-drug chemotherapy in LC.

The ICE study aimed to assess the safety and tolerability of administering ipilimumab intravenously in association with carboplatin and etoposide as a first-line therapy in ES-SCLC [224]. The 38 patients treated with ipilimumab showed a median PFS of 6.9 months. irPFS and median OS were 7.3 and 17.0 months, respectively. Regarding the toxicity, 69.2% of the patients developed grade 3 or 4 irAEs. Overall, the study demonstrated the efficacy of ipilimumab plus carboplatin and etoposide as a first-line treatment in ES-SCLC patients [224].

The CA184-156 phase III study demonstrated poor results in terms of efficacy [225]. The 476 patients treated with ipilimumab in combination with chemotherapy showed a median OS and median PFS of 11.0 and 4.6 months, respectively. The 478 patients treated with a placebo plus chemotherapy showed a median OS and median PFS of 10.9 and 4.4 months, respectively [225]. Regarding the toxicity, 27% of patients treated with ipilimumab plus chemotherapy developed any grade irAEs. Overall, the results suggested no therapeutic advantages following ICI treatment [225].

Completed as well as undergoing clinical studies are meant to assess the safety and efficacy of ipilimumab and other anti-CTLA4 antibodies as first-line treatments in advanced SCLC and NSCLC in combination with chemotherapy or other ICIs (Supplementary Table S2).

### 3.2. Programmed Death 1 and Programmed Death Ligand 1 Inhibitors

ICIs against the Programmed Death 1 (PD-1)/Programmed Death Ligand 1 (PD-L1) checkpoint are currently approved therapies for advanced LC (Figure 2). Between 2015 and 2017, the FDA approved nivolumab and pembrolizumab as anti PD-1 mAbs for the treatment of metastatic NSCLC. Additionally, between 2016 and 2019, atezolizumab and durvalumab were approved as anti PD-L1 mAbs for NSCLC [226,227,228]. Later, in 2020, durvalumab was also approved as a first-line treatment for ES-SCLC in combination with chemotherapy [229].

Nivolumab is a well-tolerated humanized anti-PD-1 mAb. Three phase III clinical trials, i.e., CheckMate 017, CheckMate 057, and CheckMate 078, demonstrated that nivolumab had increased efficacy compared to docetaxel in LC.

CheckMate 017 aimed to assess both the safety and efficacy of nivolumab compared to docetaxel in NSCLC patients after the failure of prior platinum-based chemotherapy. The first results demonstrated the improved efficacy of nivolumab [230]. The 131 patients treated with nivolumab showed a median OS of 9.2 months compared with 6.0 months in the docetaxel-treated group. The recorded ORR was 20% for the nivolumab-treated patients compared with 9% for the docetaxel arm. The observed median PFSs were 3.5 and 2.8 months for the nivolumab- and docetaxel-treated groups, respectively [230]. With regard to toxicity, TRAEs of grades 3 and 4 were reported in 7% of the nivolumab group and 55% of the docetaxel group [230].

CheckMate 057, a randomized, open-label, international, phase III clinical trial, was conducted to assess the safety and efficacy of nivolumab compared with docetaxel in patients with metastatic non-SqCC NSCLC [227]. The 287 patients treated with nivolumab showed a median OS of 12.2 months compared with 9.4 months found in the docetaxel-treated group. The confirmed ORRs were 19% and 12% in the nivolumab- and docetaxel-treated groups, respectively. The median PFSs were 2.3 and 4.2 months, respectively. Regarding the toxicity, only 7% of patients treated with nivolumab developed severe TRAEs compared with 20% in the docetaxel-treated group [227].

Long-term data analysis of advanced NSCLC from the two studies CheckMate 017 and CheckMate 057 upon a follow-up of three or more years was published in 2018. Overall, the patients were randomized (1:1) to receive either nivolumab or docetaxel, until progression or discontinuation. After 40.3 months of minimum follow-up, the 3-year OS rates were 17% versus 8% for the pooled population treated with nivolumab and docetaxel, respectively, with a slightly higher hepatotoxicity recorded in the nivolumab group (10% versus 6%) [231].

Similarly, the phase III open-label, randomized study CheckMate 078 evaluated the safety and efficacy of nivolumab compared with docetaxel in patients with advanced and metastatic NSCLC who progressed during or after platinum-based doublet chemotherapy. The results published in 2019 evidenced the superiority of nivolumab [232]. The 338 patients treated with nivolumab showed a median OS of 12.0 months compared with 9.6 months found for the docetaxel-treated group. In particular, the subgroup of patients with a PD-L1 expression of 1% or higher, treated with nivolumab, showed a median OS of 12.3 months, whereas the subgroup of patients with a PD-L1 expression lower than 1% showed a median OS of 11.4 months [232].

Furthermore, patients treated with nivolumab showed an ORR of 16.6% compared with 4.2% in the docetaxel-treated group. The median PFS was identical in both groups, but the estimated PFS rates at 6 months were 29% and 23% in nivolumab- and docetaxel-treated groups, respectively. Regarding the toxicity, 9% of the patients treated with nivolumab developed severe TRAEs of any grade compared with 16% in the docetaxel-treated subjects. Overall, the results were consistent with the ones from the above-reported CheckMate 078 study [232].

Pembrolizumab was the first anti-PD-1 mAb approved by the FDA in 2014 for advanced melanoma patients. In 2017, its use was approved for any unresectable or metastatic solid tumor, including NSCLC [233].

Regarding LC, several studies have been conducted. KEYNOTE-042 is a randomized, open-label, phase III trial, evaluating the safety and efficacy of pembrolizumab versus chemotherapy for previously untreated, PD-L1-expressing, locally advanced or metastatic NSCLC. The patients with a tumor proportion score (TPS) of 50% or higher treated with pembrolizumab showed a median survival duration of 20.0 months compared with 12.2 months for the chemotherapy treated group. The patients with a TPS of 20% or higher treated with pembrolizumab showed a median survival of 17.7 months compared with 13.0 months for the chemotherapy-treated group. Patients with a TPS of 1% or higher treated with pembrolizumab showed a median survival of 16.7 months compared with 12.1 months for the chemotherapy-treated group [234].

The median PFSs were 7.1 and 6.4 months in patients with a TPS of 50% or higher treated with pembrolizumab or chemotherapy, respectively. The median PFSs were 6.2 and 6.6 months in patients with a TPS of 20% or higher and 5.4 and 6.5 months in patients with a TPS of 1% or higher for pembrolizumab-treated and chemotherapy-treated patients, respectively. Regarding the toxicity, 63% of the patients treated with pembrolizumab developed TRAEs of any grade compared with 90% observed in the chemotherapy-treated group. Overall, the findings demonstrated a survival benefit and manageable safety profile for pembrolizumab, including in patients with a TPS as low as 1% [234].

Three phase III randomized studies, KEYNOTE-021, KEYNOTE-189, and KEYNOTE-407, aimed to assess the efficacy of pembrolizumab in combination with chemotherapy versus chemotherapy alone. The results, published in 2020, analyzed the outcomes of the three pooled studies specifically for PD-L1-negative patients with advanced NSCLC [235]. The 256 patients treated with pembrolizumab in combination with chemotherapy showed a median OS and PFS of 19.0 and 6.9 months, respectively, compared with 11.4 and 5.8 months for the chemotherapy group. The ORR was 50.0% for the pembrolizumab plus chemotherapy group compared with 29.8% for the chemotherapy-treated group, and the assessed DOR was 8.5 and 6.9 months in the two groups, respectively [235]. Regarding the toxicity, 29.0% of patients treated with pembrolizumab in combination with chemotherapy developed TRAEs of any grade compared with 12.4% for the chemotherapy-treated group. Overall, the results demonstrated that pembrolizumab plus chemotherapy showed a greater efficacy compared to chemotherapy alone in PD-L1-negative NSCLC [235].

The two trials PEMBRO-RT (phase II) and MDACC (phase I/II) evaluated the efficacy of pembrolizumab with or without radiotherapy in metastatic NSCLC patients. When the trials were analyzed separately, benefits were recorded in the combination arm of both studies. However, the studies were reduced in sample size, and this did not allow them to reach a statistical significance [236,237].

By considering both studies, the results obtained from 148 patients were analyzed. Overall, patients who received pembrolizumab with radiotherapy had a median OS and PFS of 19.2 and 9.0 months, respectively, whereas the pembrolizumab-alone group showed a median OS and PFS 8.7 and 4.4 months, respectively. Additionally, the pembrolizumab plus radiotherapy-treated group showed the best mass reduction and control rate (41.7% and 65.3%, respectively, compared to 19.7% and 43.4%). Hence, combining radiotherapy with venous pembrolizumab administration increased outcomes in patients with metastatic NSCLC without worsening the toxicity [238].

In 2016, atezolizumab was approved by the FDA for locally advanced or metastatic urothelial carcinoma. Later, in 2020, it was approved for locally advanced or metastatic NSCLC (first- and second-line regimens) [239]. Two clinical trials, the phase II POPLAR and the phase III OAK, assessed both the safety and efficacy of atezolizumab (Tecentriq) in comparison with docetaxel in patients with locally advanced or metastatic NSCLC who progressed after platinum chemotherapy [226,240].

The results from the POPLAR study were published in 2017 [226]. The 144 patients treated with atezolizumab showed an OS of 12.6 months, whereas the docetaxel-treated patients showed an OS of 9.7 months. In addition, patients with higher PD-L1 expression had an increase in OS, ORR, and PFS. The PFSs were 7.8 months and 3.9 months and the ORRs were 38% and 13% for the for the atezolizumab- and docetaxel-treated arms, respectively. With regard to toxicity, 8% of the patients treated with atezolizumab developed grade 3 or 4 TRAEs compared with 22% of the patients treated with docetaxel. Overall, atezolizumab was well tolerated, with a safety profile different from chemotherapy [226].

The OAK trial results were published in 2017 [240]. The 425 patients treated with atezolizumab showed an OS of 15.7 months compared with 10.3 months observed in the docetaxel-treated group. In addition, an increase of median OS in patients with higher PD-L1 expression treated with atezolizumab was observed (20.5 months). The median PFSs were 2.8 and 4.0 months for the atezolizumab- and docetaxel-treated groups, respectively. Regarding the toxicity, 15% of the patients treated with atezolizumab developed grade 3 or 4 TRAEs compared with the 43% found in the docetaxel arm. Overall, this study was the first one to demonstrate a significant improvement of OS in atezolizumab-treated patients, regardless of PD-L1 expression levels, with a favorable safety profile [240].

The IMpower133 randomized phase III study evaluated the safety and effectiveness of administering atezolizumab combined with two-drug chemotherapy (carboplatin plus etoposide) to treatment-naïve ES-SCLC [241]. The 201 patients treated with atezolizumab plus chemotherapy showed a median OS and a median PFS of 12.3 and 5.2 months, respectively, whereas patients treated with placebo plus chemotherapy showed values of 10.3 and 4.3 months for median OS and median PFS, respectively. Regarding the toxicity, the safety profile of the combination was comparable with the one obtained with the individual agents. Therefore, the addition of atezolizumab to chemotherapy in the first-line treatment of ES-SCLC showed significantly longer OS and PFS, with manageable safety issues [241].

Finally, in 2018, durvalumab (anti PD-L1) was approved after concurrent chemotherapy and radiation for unresectable stage III NSCLC patients. The approval was based on the results obtained from the PACIFIC randomized double-blind study conducted on 713 patients [242]. Overall, durvalumab resulted in a significantly longer OS than placebo, while the TRAEs were comparable [242]. A follow-up analysis after four years confirmed that durvalumab after chemoradiotherapy had a durable PFS and sustained OS benefits with 49.6% of patients treated with durvalumab remaining alive after 4 years (versus 36.3% in the placebo group) and 35.3% remaining alive and free from progression (versus 19.5% in the placebo group) [243].

Recently, in 2020, durvalumab was approved in combination with etoposide and either carboplatin or cisplatin as a first-line treatment of patients with ES-SCLC. The efficacy and safety were proven by the results obtained by the CASPIAN randomized, open-label, phase III, multicenter trial [244]. The combination with durvalumab significantly improved the OS in patients with ES-SCLC compared with the clinically relevant control group, with manageable safety issues [244]. Several ongoing trials are currently evaluating the therapeutic efficacy of several anti-PD-L1 antibodies, alone or in combination with anti-CTLA4 or anti-PD1, for the treatment of NSCLC and SCLC, and they are reported in Supplementary Table S2 [245,246].

## 4. Combination Therapies Using Immune Checkpoint Inhibitors plus Chemo- and/or Targeted Therapies

As widely described in the previous paragraphs, treatments with chemotherapy, targeted therapies, or immunotherapy are often accompanied by drug resistance mechanisms which limit therapeutic efficacy [247]. In order to overcome drug resistance, combination treatments have been proposed, especially using ICIs. At present, ICIs are tested in combination with another ICI with a different target. Additionally, as reported in Supplementary Table S2, ICIs are also tested in combination with standard chemotherapy and radiotherapy, but also targeted therapies against the Vascular Endothelial Growth Factor (VEGF) (e.g., Lucentis/ranibizumab and Avastin/bevacizumab) [248].

Two clinical trials evaluated the combination of nivolumab with ipilimumab: CheckMate 032 and CheckMate 9LA. CheckMate 032 is a randomized, open label study to establish both the safety and efficacy of nivolumab monotherapy versus nivolumab in combination with ipilimumab in patients with advanced or metastatic solid tumors, including SCLC. The 147 patients treated with nivolumab alone showed an ORR of 11.6%; the 96 patients treated with the combination therapy showed an ORR of 21.9%; the median DORs were 15.8 and 10.0 months in the two groups, respectively, whereas the median OSs were 5.7 months and 4.7 months, respectively; finally, the median PFSs were 1.4% and 1.5% respectively. Regarding the toxicity, grade 3–4 TRAEs occurred in 12.9% of patients treated with nivolumab and 37.5% of those treated with nivolumab plus ipilimumab. Overall, the study showed that the OS and PFS were similar in both groups and TRAEs were more common in the combination therapy [249].

CheckMate 9LA is an international, randomized, open-label, phase III trial to assess both the safety and efficacy of administering two cycles of chemotherapy with or without nivolumab with ipilimumab in patients with NSCLC [250]. The 361 patients treated with the two ICIs plus chemotherapy combination therapy showed a median OS of 14.1 months compared with 10.7 months for the other group. Regarding the toxicity, 30% of the patients treated with the combination therapy developed severe TRAEs compared with the 18% of the chemotherapy group. Overall, nivolumab plus ipilimumab combined with chemotherapy provided a significantly improved OS compared to chemotherapy alone. Moreover, this ICI combination showed a favorable risk–benefit profile. Hence, this regimen might be offered as a first-line treatment option for patients with advanced NSCLC [250].

In 2017, the KEYNOTE-598 phase III randomized, double-blind study aimed to test whether ipilimumab might improve the efficacy of pembrolizumab, which is a standard first-line therapy for metastatic NSCLC patients with PD-L1 TPS > 50% [251]. The study demonstrated no therapeutic advantages compared to standard schedules. Furthermore, grade 3–5 irAEs occurred in 62.4% of pembrolizumab plus ipilimumab recipients versus 50.2% of pembrolizumab plus placebo recipients and led to death in 13.1% versus 7.5% of patients. Therefore, the trial was stopped due to the toxicities and weak results obtained [251].

Additionally, the ARCTIC phase III, randomized, open-label study tested the combination of PD-L1 and CTLA-4 mAbs in LC. In this study, durvalumab was administered with tremelimumab as a third-line treatment compared to durvalumab alone in patients with metastatic NSCLC, without known EGFR- or ALK-activating mutations and/or rearrangements. The study, whose results were published in 2020, was divided into two sub-studies (A and B) [252].

In sub-study A, 126 patients with 25% or higher percentage of PD-L1 expressing tumor cells (TC) were randomly allocated (1:1) to receive either durvalumab or chemotherapy (gemcitabine and vinorelbine or erlotinib), whereas in sub-study B, 469 patients with PD-L1 expressing TC lower than 25% were randomly allocated (3:2:2:1) to receive either durvalumab plus tremelimumab, chemotherapy, durvalumab alone, or tremelimumab alone. Overall, this study demonstrated that durvalumab as a monotherapy had clinically meaningful improvement in OS and PFS versus chemotherapy treatment. Furthermore, the combination therapy (durvalumab plus tremelimumab) had a numerical improvement in OS versus the chemotherapy treatment, although no statistically significant differences were observed [252].

MYSTIC, a phase III study, also aimed to assess the efficacy of combining durvalumab plus tremelimumab in the treatment of advanced NSCLC. The results were encouraging, although the trial did not validate the primary endpoints [253]. Indeed, the 371 patients treated with the combination ICI therapy showed a median OS of 11.9 months compared with 16.3 and 12.9 months for the durvalumab alone and chemotherapy treated groups, respectively. The median PFSs were 3.9 and 5.4 months for patients treated with the combination therapy and for those treated with chemotherapy, respectively. No statistically significant differences in OS and PFS were observed. Regarding the toxicity, 28.3% patients treated with the combination therapy developed TRAEs compared with the 13.6% and 3.4% of patients treated with durvalumab or chemotherapy alone, respectively. Although the primary endpoints have not been achieved, the study established a TMB threshold of ≥20 mutations per MB for optimal OS benefit with durvalumab plus tremelimumab combination therapy [253].

In addition to combinations between ICIs and chemotherapy/radiotherapy/anti-VEGF, several combinations of ICIs with targeted therapies are currently under clinical evaluation, as reported in Supplementary Table S3. The studies are all phase I and II, plus a phase III study, which is evaluating the efficacy and safety profile of a MET inhibitor (sitravatinib) plus nivolumab in metastatic non-squamous LC patients who have previously experienced disease progression or after platinum-based chemotherapy and ICI-based therapy (NCT03906071, Supplementary Table S3).

Notably, the current clinical trials are also proving the efficacy of a novel generation of mAbs, called bispecific antibodies, as they are capable of targeting two molecules simultaneously (e.g., AK104 and KN046 PD-1/CTLA-4 bispecific antibodies; AK112 PD-1/VEGF bispecific antibody; Supplementary Table S2). Finally, the combination of ICIs with other immunotherapies is also presently under clinical assessment (e.g., ICIs in combination with the peptide vaccine IO102; MK-4830, a mAb targeting the myeloid ILT4 receptor; MK-5890, an anti-CD27 agonist; SEA-TGT, an anti-TIGIT antibody; or CAB-AXL-ADC, an AXL-targeted antibody drug conjugate; Supplementary Table S2).

## 5. Microbiota Modulation in Lung Cancer

As reported above, within the last ten years, ICI-based therapy significantly widened the therapeutic options available for advanced or metastatic LC. For this reason, ICIs are considered a breakthrough advancement in the fight against LC. However, a significant fraction of patients does not respond to ICIs or develops severe irAEs which might compromise the compliance to therapy [254]. Consequently, it becomes of pivotal importance to identify the specific subset of LC patients which can truly benefit from immunotherapy [255]. The identification of a range of predictive reliable biomarkers might help to select the class of responders who can be safely treated with ICIs [256]. More than 40 ICI-related biomarkers are currently under investigation, and they can be classified as tumor-related biomarkers and microenvironment-related biomarkers [257].

Among these, only a few are currently validated in phase III trials for LC. One of the most important is the IHC evaluation of PD-L1 protein expression in APCs and LC cells [234,258,259]. However, PD-L1 detection shows several limitations due to the non-homogeneous expression, which depends on the biopsy site or the specific anti-PD-L1 antibody used for the assay [260,261].

A second biomarker used in LC trials to test ICI efficacy is the TMB [262]. A greater TMB indicates more tumor neoantigens potentially presented by APCs and recognized by the immune system, including the T cell response [263]. In this case, a universal scale for TMB assessment, as well as a standardization of the TMB calculation method, are further needed [264,265].

More recently, great interest was devoted to the study of human microbiota composition, which can significantly regulate different cellular and molecular functions, including tumor initiation, immune stimulation, and response to therapies [266]. Regarding LC, both gut microbiota and lung microbiota play an extremely important role in the pathogenesis of the disease. It has been observed that a healthy lung or gut microbiota composition is associated with a healthy individual, whereas dysbiosis, consisting in the disequilibrium of the abundance and prevalence of pro-inflammatory pathogens, might promote an unhealthy status triggering tumorigenesis [267,268].

The host’s immune system is tightly interconnected with the microbiome, especially the gut microbiome, which, in turn, modulates the immune system, helping to develop tolerance against self-antigens [269]. New breakthrough studies recently uncovered the potential of manipulating the gut microbiota composition to improve the efficacy of ICIs in metastatic and advanced melanoma patients [270,271]. Several strategies are tested, including the administration of single probiotics or multi-strain consortia, and fecal microbiota transplantation [272]. The reported findings are very promising and have paved the way towards a growing list of novel studies aiming to assess the efficacy of manipulating the gut microbial composition to improve the efficacy of ICIs also in LC (Supplementary Table S4).

**Figure 3:**
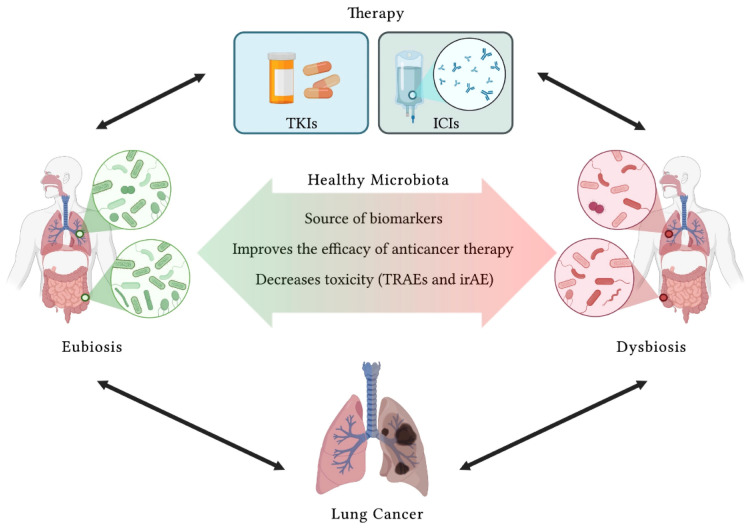
Positive roles of healthy gut and lung microbiota in lung cancer patients treated with tyrosine kinase inhibitors (TKIs) and/or immune-checkpoint inhibitors (ICIs). Lung and gut microbiota influence the response to targeted therapies and ICIs. The analysis of gut and lung microbiota composition could be a reliable source of diagnostic and prognostic biomarkers for LC. Eubiosis (microbial balance) versus dysbiosis (microbial unbalance) significantly influences the efficacy and toxicity of both TKIs and ICIs in lung cancer. The positive modulation of gut and lung microbiota is associated with the improvement of the efficacy of anticancer therapies and a decreased incidence of therapy-related adverse events (TRAEs) and immune-related adverse events (irAEs).

In the near future, we will observe a radical change in the management of cancer patients, including LC patients, with the introduction of a multidisciplinary approach to treatments and the development of tailored therapies [273,274]. Cancer will be managed in a more holistic way, taking into account that the tumor develops within a complexity of interactions happening within the human host, such as with the immune system and the gut/lung microbiota. The specific microbiota composition can be considered as a novel source of LC biomarkers. Additionally, its modulation towards a beneficial/eubiotic population might be used to improve the immune health and, hence, the overall efficacy of anticancer therapies, and in particular ICIs in LC (Figure 3).

## 6. Main Concepts and Future Perspectives

**Figure 4:**
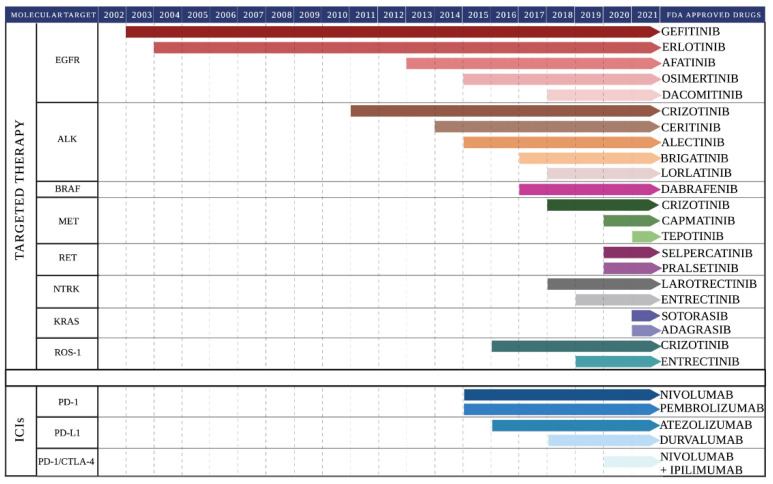
Timeline of the FDA-approved targeted therapies and immune-checkpoint inhibitors (ICIs) for the treatment of lung cancer. In dark blue, top row, the timeline (from 2002 to 2021) is indicated. On the left, the approved drugs are indicated, divided based on their molecular target. On the right each single drug name is reported. In the middle, an arrow is associated to each drug and the year of approval (different colors).

The personalization of anticancer treatments is the main goal of the current research. A number of targeted therapies and ICIs are currently approved for the treatment of LC of different stages and histological types (Figure 4).

Novel technologies have allowed the genome mapping and identification of genetic mutations/rearrangements. The molecular screening for selected oncogenic drivers potentially targetable is a fundamental step of diagnostic and therapeutic processes. The approval of a growing number of small molecules able to target and selectively inhibit altered TKs, hence blocking the growth and metastatization of LC cells, has ameliorated the life expectancy of LC patients.

Targeted therapies changed the way cancer patients are treated. Small molecules target specific alterations within a given receptor; therefore, the toxicity is reduced compared with the systemic toxicity associated with the administration of standard chemotherapy and radiotherapy. Given their specific pharmacokinetics, the vast majority of targeted therapies have a good compliance with an oral route of administration. Despite these great advantages, the use of TKIs is often coupled with the development of resistance; therefore, combination therapies are currently investigated to overcome this issue [275].

As widely described in the previous chapters, targeted therapies have significantly improved the survival of LC patients by reducing the toxic effects related to standard chemotherapy. Although several selective inhibitors are currently approved for the treatment of different molecular forms of LC, treatment-specific adverse events were often observed (Table 1).

Notably, the last ten years have witnessed the development of immunotherapy based on the administration of specific ICI mAbs. ICIs target the immune checkpoint responsible for the suppression of T cell response, thereby reactivating the host’s immune system to recognize and efficiently eliminate LC cells. The ICIs, alone or in combination with other ICIs and standard chemotherapy or radiotherapy, have been proven effective in reducing tumor burden and improving LC patients’ outcomes [276].

Furthermore, novel ongoing studies are currently testing the efficacy and safety of administering ICIs in combination with specific targeted therapies or chemotherapy [277] (Supplementary Table S3). The results of these studies might reveal novel strategies to treat LC, reducing the common side effects of ICIs (Table 2).

In a more holistic view, the specific lung and gut microbial population might be considered a powerful source of biomarkers for the diagnosis and prognosis of LC. Microbial populations are interconnected with the host, especially given their capacity to modulate the immune system. Novel findings are paving the way to actively manipulate the gut microbiome to improve the efficacy of anticancer treatments, and, more specifically, of ICIs [278] (Supplementary Table S4).

Overall, all these combined therapeutic approaches will lead to the development of multi-agent anticancer therapies which will be tailored to a patient’s specific features and molecular background. In fact, each cancer patient is unique in terms of their genetically mutated landscape, immune system behavior, and gut microbial health.

## Figures and Tables

**Table 1:** Currently approved targeted therapies and related side effects.

Molecular Target	Trial Type	Agents	Side Effects	References
EGFR	Phase II/III	erlotinib	Rash, diarrhea, anorexia, fatigue, dyspnea, cough, nausea, vomiting	[46,48,54,56,62,63]
	Phase II/III	gefitinib	Abdominal or stomach pain or tenderness, clay-colored stools, dark urine, diarrhea, severe Fever, headache, nausea and vomiting, weakness, yellow eyes or skin	[49,50,56,57,58,59,62,63]
	Phase III	afatinib	Diarrhea, paronychia, skin rush, stomatitis, pruritus	[53,54,55,56]
	Phase III	dacomitinib	Skin lesions, diarrhea, cough, fever, headache, nasal congestion, sore throat sores, ulcers, or white spots on the lips, tongue, or inside the mouth, weakness	[57,58,59]
	Phase III	osimertinib	Low blood cell counts, pain, diarrhea, tiredness, cough, mouth sores, dry skin, rash	[62,63]
ALK	Phase II/III	crizotinib	Diarrhea, vomiting, nausea, vision disorder, headache, musculoskeletal pain, stomatitis, fatigue	[77,78,86,94]
	Phase I/II/III	alectinib	Tiredness, constipation, muscle pain, anemia, swelling	[86,88]
	Phase II	brigatinib	Asthenia, blurred vision, cough, nausea, diarrhea, hyperglycemia, hypertension, myalgia, fatigue	[88]
	Phase I/II	ceritinib	Abdominal pain, diarrhea, hyperglycemia, increased serum ALT and AST, nausea, vomiting	[88]
	Phase I/II	erlotinib	Anorexia, eye infection, diarrhea, vomiting, skin rash, nausea	[88]
	Phase III	lorlatinib	Diarrhea, neuropathy, obesity, cough, edema, cognitive disorders	[92,93,94]
BRAF	Phase II	vemurafenib	Alopecia, skin photosensitivity, hyperkeratosis, arthralgia, fatigue, rash, diarrhea, headache, skin papilloma	[110,111]
	Phase II	dabrafenib	Fever, hyperglycemia, squamous cell carcinoma	[106,113,114]
MEK	Phase II	trametinib	Rash, swelling (eye, face, tongue), cough, trouble breathing	[113,114]
RET	Phase I/II	selpercatinib	Bleeding, constipation, hair loss, hyperglycemia, difficulty in breathing, pneumonia, increased AST and ALT levels, hyponatremia, neutropenia	[130]
	Phase I/II	pralsetinib	Bone pain, cramping, swelling, stomatitis, diarrhea, weakness	[131]
MET	Phase I	crizotinib	Abdominal pain, diarrhea, hyperglycemia, increased serum ALT and AST, nausea, vomiting	[146]
	Phase II	capmatinib	Edema, nausea, vomiting, high creatinine level, skin rush, stomach pain	[149]
	Phase II	tepotinib	Cough, anxiety, dark urine, sore throat, trouble breathing, fever, muscle and bone pain	[150]
NTRK	Phase I/II	larotrectinib	Diarrhea, nausea, dizziness, vomiting, anemia, increased AST and ALT levels, cough, constipation, fatigue	[156]
	Phase I/II	entrectinib	Vision disorders, diarrhea, nausea, edema, cognitive disorders, vomiting, dizziness, dyspnea, myalgia	[159,187]
KRAS	Phase I/II	adagrasib	Nausea, diarrhea, vomiting, fatigue, increased ALT, hyponatremia	[177]
	Phase I/II	sotorasib	Pneumonia, diarrhea, muscle and bone pain, nausea, fatigue, increased AST and ALT levels, respiratory failure, heart diseases	[176,178]
ROS-1	Phase I/II	crizotinib	Diarrhea, vomiting, nausea, vision disorder, headache, musculoskeletal pain, stomatitis, fatigue	[184,185]
	Phase II	ceritinib	Abdominal pain, diarrhea, hyperglycemia, increased serum ALT and AST, nausea, vomiting	[186]
	Phase I/II	entrectinib	Vision disorders, diarrhea, nausea, edema, cognitive disorders, vomiting, dizziness, dyspnea, myalgia	[159,187]
	Phase I	taletrectinib	Increased AST and ALT levels, nausea, diarrhea	[193]
PI3K	Phase I	buparlisib	Hyperglycemia, fatigue, rash, anxiety, depression, mood disorders	[199]
	Phase II	MK-2206	Hyperglycemia, thrombocytopenia, fatigue, rash, nausea	[200]
	Phase II	taselisib	Diarrhea, hyperglycemia, nausea, fatigue, headache, stomatitis, vomiting, rash	[201]

**Table 2:** Currently approved immune checkpoint inhibitors and related side effects.

Molecular Target	Phase	Agents	Side Effects	References
CTLA-4	Phase II/III	ipilimumab	Diarrhea, rash, pruritus, fatigue, nausea, vomiting, decreased appetite, abdominal pain, and colitis	[222,223,224,225,249,250,251]
	Phase III	tremelimumab	Nausea, fatigue, muscle and bone pain, rash, diarrhea, neutropenia, hyponatremia	[252,253]
PD1	Phase III	nivolumab	Rash, weakness, muscle and bone pain, diarrhea, respiratory tract infection, fever	[227,230,232,249,250]
	Phase II/III	pembrolizumab	Fatigue, muscle and bone pain, rash, diarrhea, fever, pruritus, abdominal pain, nausea	[234,235,236,237,251]
PD-L1	Phase II/III	atezolizumab	Fatigue, nausea, infection, fever, constipation, anemia, pneumonia	[226,240,241]
	Phase II/III	durvalumab	Rash, diarrhea, pruritus, anemia, neutrophilia, fatigue, dyspnea, asthenia, loss of appetite	[243,244]

## Data Availability

The data reported in the manuscript are available from the corresponding author on request. The original contributions presented in the study are publicly available. These data can be found at: www.pubmed.com (accessed on 11 September 2022); www.clinicaltrials.gov (accessed on 20 October 2022).

## Supplementary Materials

The following supporting information can be downloaded at: https://www.mdpi.com/article/10.3390/pharmaceutics15041252/s1, Supplementary Table S1: Ongoing clinical studies on targeted therapies registered at clinicaltrials.gov; Supplementary Table S2: Ongoing clinical studies on immune-checkpoint inhibitors registered at clinicaltrials.gov; Supplementary Table S3: Ongoing clinical studies on targeted therapies and immune-checkpoint inhibitors in combination registered at clinicaltrials.gov; Supplementary Table S4: Ongoing clinical studies on microbiota modulating drugs in combination with LC therapy registered at clinicaltrials.gov.

## References

[1] Siegel R.L., Miller K.D., Wagle N.S., Jemal A. (2023). Cancer statistics, 2023. CA Cancer J. Clin..

[2] Travis W.D., Brambilla E., Nicholson A.G., Yatabe Y., Austin J.H.M., Beasley M.B., Chirieac L.R., Dacic S., Duhig E., Flieder D.B. (2015). The 2015 World Health Organization Classification of Lung Tumors: Impact of Genetic, Clinical and Radiologic Advances Since the 2004 Classification. J. Thorac. Oncol..

[3] Travis W.D., Brambilla E., Riely G.J. (2013). New Pathologic Classification of Lung Cancer: Relevance for Clinical Practice and Clinical Trials. J. Clin. Oncol..

[4] Petersen I. (2011). The morphological and molecular diagnosis of lung cancer. Dtsch. Arztebl. Int..

[5] Travis W.D., Brambilla E., Noguchi M., Nicholson A.G., Geisinger K.R., Yatabe Y., Beer D.G., Powell C.A., Riely G.J., Van Schil P.E. (2011). International association for the study of lung cancer/american thoracic society/european respiratory society international multidisciplinary classification of lung adenocarcinoma. J. Thorac. Oncol..

[6] Travis W.D., Brambilla E., Burke A.P., Marx A., Nicholson A.G. (2015). Introduction to The 2015 World Health Organization Classification of Tumors of the Lung, Pleura, Thymus, and Heart. J. Thorac. Oncol..

[7] Siegel R.L., Miller K.D., Jemal A. (2019). Cancer statistics, 2019. CA Cancer J. Clin..

[8] Zheng M. (2016). Classification and Pathology of Lung Cancer. Surg. Oncol. Clin. N. Am..

[9] Hutchinson B.D., Shroff G.S., Truong M.T., Ko J.P. (2019). Spectrum of Lung Adenocarcinoma. Semin. Ultrasound CT MRI.

[10] Dela Cruz C.S., Tanoue L.T., Matthay R.A. (2011). Lung Cancer: Epidemiology, Etiology, and Prevention. Clin. Chest. Med..

[11] Travis W.D. (2011). Classification of Lung Cancer. Semin. Roentgenol..

[12] Byers L.A., Rudin C.M. (2015). Small cell lung cancer: Where do we go from here?. Cancer.

[13] Travis W.D. (2020). Lung Cancer Pathology. Clin. Chest. Med..

[14] Collins L.G., Haines C., Perkel R., Enck R.E. (2007). Lung cancer: Diagnosis and management. Am. Fam. Physician.

[15] Wadowska K., Bil-Lula I., Trembecki Ł., Śliwińska-Mossoń M. (2020). Genetic Markers in Lung Cancer Diagnosis: A Review. Int. J. Mol. Sci..

[16] Rossi G., Pelosi G., Barbareschi M., Graziano P., Cavazza A., Papotti M. (2013). Subtyping Non–Small Cell Lung Cancer. Int. J. Surg. Pathol..

[17] van Meerbeeck J.P., Fennell D.A., De Ruysscher D.K. (2011). Small-cell lung cancer. Lancet.

[18] Gridelli C., Rossi A., Carbone D.P., Guarize J., Karachaliou N., Mok T., Petrella F., Spaggiari L., Rosell R. (2015). Non-small-cell lung cancer. Nat. Rev. Dis. Prim..

[19] Jemal A., Ma J., Rosenberg P.S., Siegel R., Anderson W.F. (2012). Increasing Lung Cancer Death Rates Among Young Women in Southern and Midwestern States. J. Clin. Oncol..

[20] Haddadin S., Perry M.C. (2011). History of Small-Cell Lung Cancer. Clin. Lung Cancer.

[21] de Sousa V.M.L., Carvalho L. (2018). Heterogeneity in Lung Cancer. Pathobiology.

[22] Alberg A.J., Brock M.V., Samet J.M. (2005). Epidemiology of Lung Cancer: Looking to the Future. J. Clin. Oncol..

[23] Malhotra J., Malvezzi M., Negri E., La Vecchia C., Boffetta P. (2016). Risk factors for lung cancer worldwide. Eur. Respir. J..

[24] Falzone L., Marconi A., Loreto C., Franco S., Spandidos D.A., Libra M. (2016). Occupational exposure to carcinogens: Benzene, pesticides and fibers (Review). Mol. Med. Rep..

[25] Delva F., Andujar P., Lacourt A., Brochard P., Pairon J.C. (2016). Occupational risk factors for lung cancer. Rev. Mal. Respir..

[26] Falzone L., Bordonaro R., Libra M. (2023). SnapShot: Cancer chemotherapy. Cell.

[27] Kalemkerian G.P., Narula N., Kennedy E.B., Biermann W.A., Donington J., Leighl N.B., Lew M., Pantelas J., Ramalingam S.S., Reck M. (2018). Molecular Testing Guideline for the Selection of Patients With Lung Cancer for Treatment With Targeted Tyrosine Kinase Inhibitors: American Society of Clinical Oncology Endorsement of the College of American Pathologists/International Association for the Study of Lung Cancer/Association for Molecular Pathology Clinical Practice Guideline Update. J. Clin. Oncol..

[28] Lim S.W., Ahn M.J. (2019). Current status of immune checkpoint inhibitors in treatment of non-small cell lung cancer. Korean J. Intern. Med..

[29] Chen Q., Wang C., Chen G., Hu Q., Gu Z. (2018). Delivery Strategies for Immune Checkpoint Blockade. Adv. Healthc. Mater..

[30] Seetharamu N., Budman D.R., Sullivan K.M. (2016). Immune checkpoint inhibitors in lung cancer: Past, present and future. Futur. Oncol..

[31] Ruiz-Cordero R., Devine W.P. (2020). Targeted Therapy and Checkpoint Immunotherapy in Lung Cancer. Surg. Pathol. Clin..

[32] Carrot-Zhang J., Yao X., Devarakonda S., Deshpande A., Damrauer J.S., Silva T.C., Wong C.K., Choi H.Y., Felau I., Robertson A.G. (2021). Whole-genome characterization of lung adenocarcinomas lacking the RTK/RAS/RAF pathway. Cell. Rep..

[33] Planchard D., Popat S., Kerr K., Novello S., Smit E.F., Faivre-Finn C., Mok T.S., Reck M., Van Schil P.E., Hellmann M.D. (2018). Metastatic non-small cell lung cancer: ESMO Clinical Practice Guidelines for diagnosis, treatment and follow-up. Ann. Oncol..

[34] Lindeman N.I., Cagle P.T., Aisner D.L., Arcila M.E., Beasley M.B., Bernicker E.H., Colasacco C., Dacic S., Hirsch F.R., Kerr K. (2018). Updated Molecular Testing Guideline for the Selection of Lung Cancer Patients for Treatment With Targeted Tyrosine Kinase Inhibitors: Guideline From the College of American Pathologists, the International Association for the Study of Lung Cancer, and the Association for Molecular Pathology. Arch. Pathol. Lab. Med..

[35] Wee P., Wang Z. (2017). Epidermal Growth Factor Receptor Cell Proliferation Signaling Pathways. Cancers.

[36] Goffin J.R., Zbuk K. (2013). Epidermal growth factor receptor: Pathway, therapies, and pipeline. Clin. Ther..

[37] Wang Z. (2017). ErbB Receptors and Cancer. Methods Mol. Biol..

[38] Soo R.A., Kubo A., Ando M., Kawaguchi T., Ahn M.J., Ou S.I. (2017). Association Between Environmental Tobacco Smoke Exposure and the Occurrence of EGFR Mutations and ALK Rearrangements in Never-smokers With Non–Small-cell Lung Cancer: Analyses From a Prospective Multinational ETS Registry. Clin. Lung Cancer.

[39] Gandhi J., Zhang J., Xie Y., Soh J., Shigematsu H., Zhang W., Yamamoto H., Peyton M., Girard L., Lockwood W.W. (2009). Alterations in genes of the EGFR signaling pathway and their relationship to EGFR tyrosine kinase inhibitor sensitivity in lung cancer cell lines. PLoS ONE.

[40] Cho J., Chen L., Sangji N., Okabe T., Yonesaka K., Francis J.M., Flavin R.J., Johnson W., Kwon J., Yu S. (2013). Cetuximab response of lung cancer-derived EGF receptor mutants is associated with asymmetric dimerization. Cancer Res..

[41] Yang J.C., Sequist L.V., Geater S.L., Tsai C.M., Mok T.S., Schuler M., Yamamoto N., Yu C.J., Ou S.H., Zhou C. (2015). Clinical activity of afatinib in patients with advanced non-small-cell lung cancer harbouring uncommon EGFR mutations: A combined post-hoc analysis of LUX-Lung 2, LUX-Lung 3, and LUX-Lung 6. Lancet Oncol..

[42] Gately K., O′Flaherty J., Cappuzzo F., Pirker R., Kerr K., O′Byrne K. (2012). The role of the molecular footprint of EGFR in tailoring treatment decisions in NSCLC. J. Clin. Pathol..

[43] Lee C.K., Davies L., Wu Y.L., Mitsudomi T., Inoue A., Rosell R., Zhou C., Nakagawa K., Thongprasert S., Fukuoka M. (2017). Gefitinib or Erlotinib vs Chemotherapy for EGFR Mutation-Positive Lung Cancer: Individual Patient Data Meta-Analysis of Overall Survival. J. Natl. Cancer. Inst..

[44] Schuler M., Wu Y.L., Hirsh V., O’Byrne K., Yamamoto N., Mok T., Popat S., Sequist L.V., Massey D., Zazulina V. (2016). First-Line Afatinib versus Chemotherapy in Patients with Non-Small Cell Lung Cancer and Common Epidermal Growth Factor Receptor Gene Mutations and Brain Metastases. J. Thorac. Oncol..

[45] Abdelgalil A.A., Al-Kahtani H.M., Al-Jenoobi F.I. (2020). Erlotinib. Profiles Drug Subst. Excip. Relat. Methodol..

[46] Rosell R., Carcereny E., Gervais R., Vergnenegre A., Massuti B., Felip E., Palmero R., Garcia-Gomez R., Pallares C., Sanchez J.M. (2012). Erlotinib versus standard chemotherapy as first-line treatment for European patients with advanced EGFR mutation-positive non-small-cell lung cancer (EURTAC): A multicentre, open-label, randomised phase 3 trial. Lancet Oncol..

[47] Burdett S. (2014). Preoperative chemotherapy for non-small-cell lung cancer: A systematic review and meta-analysis of individual participant data. Lancet.

[48] Xiong L., Lou Y., Bai H., Li R., Xia J., Fang W., Zhang J., Han-Zhang H., Lizaso A., Li B. (2020). Efficacy of erlotinib as neoadjuvant regimen in EGFR-mutant locally advanced non-small cell lung cancer patients. J. Int. Med. Res..

[49] Xie H., Wang H., Xu L., Li M., Peng Y., Cai X., Feng Z., Ren W., Peng Z. (2018). Gefitinib Versus Adjuvant Chemotherapy in Patients With Stage II-IIIA Non-Small-Cell Lung Cancer Harboring Positive EGFR Mutations: A Single-Center Retrospective Study. Clin. Lung Cancer.

[50] Hosomi Y., Morita S., Sugawara S., Kato T., Fukuhara T., Gemma A., Takahashi K., Fujita Y., Harada T., Minato K. (2020). Gefitinib Alone Versus Gefitinib Plus Chemotherapy for Non-Small-Cell Lung Cancer With Mutated Epidermal Growth Factor Receptor: NEJ009 Study. J. Clin. Oncol..

[51] Yap T.A., Vidal L., Adam J., Stephens P., Spicer J., Shaw H., Ang J., Temple G., Bell S., Shahidi M. (2010). Phase I trial of the irreversible EGFR and HER2 kinase inhibitor BIBW 2992 in patients with advanced solid tumors. J. Clin. Oncol..

[52] Engelman J.A., Zejnullahu K., Gale C.M., Lifshits E., Gonzales A.J., Shimamura T., Zhao F., Vincent P.W., Naumov G.N., Bradner J.E. (2007). PF00299804, an irreversible pan-ERBB inhibitor, is effective in lung cancer models with EGFR and ERBB2 mutations that are resistant to gefitinib. Cancer Res..

[53] Schuler M., Yang J.C., Park K., Kim J.H., Bennouna J., Chen Y.M., Chouaid C., De Marinis F., Feng J.F., Grossi F. (2016). Afatinib beyond progression in patients with non-small-cell lung cancer following chemotherapy, erlotinib/gefitinib and afatinib: Phase III randomized LUX-Lung 5 trial. Ann. Oncol..

[54] Soria J.C., Felip E., Cobo M., Lu S., Syrigos K., Lee K.H., Göker E., Georgoulias V., Li W., Isla D. (2015). Afatinib versus erlotinib as second-line treatment of patients with advanced squamous cell carcinoma of the lung (LUX-Lung 8): An open-label randomised controlled phase 3 trial. Lancet Oncol..

[55] Felip E., Hirsh V., Popat S., Cobo M., Fülöp A., Dayen C., Trigo J.M., Gregg R., Waller C.F., Soria J.C. (2018). Symptom and Quality of Life Improvement in LUX-Lung 8, an Open-Label Phase III Study of Second-Line Afatinib Versus Erlotinib in Patients With Advanced Squamous Cell Carcinoma of the Lung After First-Line Platinum-Based Chemotherapy. Clin. Lung Cancer.

[56] Kim Y., Lee S.H., Ahn J.S., Ahn M.J., Park K., Sun J.M. (2019). Efficacy and Safety of Afatinib for EGFR-mutant Non-small Cell Lung Cancer, Compared with Gefitinib or Erlotinib. Cancer Res. Treat..

[57] Wu Y.L., Cheng Y., Zhou X., Lee K.H., Nakagawa K., Niho S., Tsuji F., Linke R., Rosell R., Corral J. (2017). Dacomitinib versus gefitinib as first-line treatment for patients with EGFR-mutation-positive non-small-cell lung cancer (ARCHER 1050): A randomised, open-label, phase 3 trial. Lancet Oncol..

[58] Mok T.S., Cheng Y., Zhou X., Lee K.H., Nakagawa K., Niho S., Lee M., Linke R., Rosell R., Corral J. (2018). Improvement in Overall Survival in a Randomized Study That Compared Dacomitinib With Gefitinib in Patients with Advanced Non-Small-Cell Lung Cancer and EGFR-Activating Mutations. J. Clin. Oncol..

[59] Mok T.S., Cheng Y., Zhou X., Lee K.H., Nakagawa K., Niho S., Chawla A., Rosell R., Corral J., Migliorino M.R. (2021). Updated Overall Survival in a Randomized Study Comparing Dacomitinib with Gefitinib as First-Line Treatment in Patients with Advanced Non-Small-Cell Lung Cancer and EGFR-Activating Mutations. Drugs.

[60] Minari R., Bordi P., Tiseo M. (2016). Third-generation epidermal growth factor receptor-tyrosine kinase inhibitors in T790M-positive non-small cell lung cancer: Review on emerged mechanisms of resistance. Transl. Lung Cancer Res..

[61] Kuiper J.L., Heideman D.A.M., Thunnissen E., Paul M.A., van Wijk A.W., Postmus P.E., Smit E.F. (2014). Incidence of T790M mutation in (sequential) rebiopsies in EGFR-mutated NSCLC-patients. Lung Cancer.

[62] Soria J.C., Ohe Y., Vansteenkiste J., Reungwetwattana T., Chewaskulyong B., Lee K.H., Dechaphunkul A., Imamura F., Nogami N., Kurata T. (2018). Osimertinib in Untreated EGFR-Mutated Advanced Non-Small-Cell Lung Cancer. N. Engl. J. Med..

[63] Ramalingam S.S., Vansteenkiste J., Planchard D., Cho B.C., Gray J.E., Ohe Y., Zhou C., Reungwetwattana T., Cheng Y., Chewaskulyong B. (2020). Overall Survival with Osimertinib in Untreated, EGFR-Mutated Advanced NSCLC. N. Engl. J. Med..

[64] Chiarle R., Voena C., Ambrogio C., Piva R., Inghirami G. (2008). The anaplastic lymphoma kinase in the pathogenesis of cancer. Nat. Rev. Cancer.

[65] Leventaki V., Bhattacharyya S., Lim M.S. (2020). Pathology and genetics of anaplastic large cell lymphoma. Semin. Diagn. Pathol..

[66] Aygun N. (2018). Biological and Genetic Features of Neuroblastoma and Their Clinical Importance. Curr. Pediatr. Rev..

[67] Golding B., Luu A., Jones R., Viloria-Petit A.M. (2018). The function and therapeutic targeting of anaplastic lymphoma kinase (ALK) in non-small cell lung cancer (NSCLC). Mol. Cancer.

[68] Hofman P. (2017). ALK in Non-Small Cell Lung Cancer (NSCLC) Pathobiology, Epidemiology, Detection from Tumor Tissue and Algorithm Diagnosis in a Daily Practice. Cancers.

[69] Sabir S., Yeoh S., Jackson G., Bayliss R. (2017). EML4-ALK Variants: Biological and Molecular Properties, and the Implications for Patients. Cancers.

[70] Soda M., Choi Y.L., Enomoto M., Takada S., Yamashita Y., Ishikawa S., Fujiwara S., Watanabe H., Kurashina K., Hatanaka H. (2007). Identification of the transforming EML4-ALK fusion gene in non-small-cell lung cancer. Nature.

[71] Sanders H.R., Li H.R., Bruey J.M., Scheerle J.A., Meloni-Ehrig A.M., Kelly J.C., Novick C., Albitar M. (2011). Exon scanning by reverse transcriptase-polymerase chain reaction for detection of known and novel EML4-ALK fusion variants in non-small cell lung cancer. Cancer Genet..

[72] Khan M., Lin J., Liao G., Tian Y., Liang Y., Li R., Liu M., Yuan Y. (2019). ALK Inhibitors in the Treatment of ALK Positive NSCLC. Front. Oncol..

[73] Paik J., Dhillon S. (2018). Alectinib: A Review in Advanced, ALK-Positive NSCLC. Drugs.

[74] Kim D.W., Tiseo M., Ahn M.J., Reckamp K.L., Hansen K.H., Kim S.W., Huber R.M., West H.L., Groen H.J.M., Hochmair M.J. (2017). Brigatinib in Patients With Crizotinib-Refractory Anaplastic Lymphoma Kinase-Positive Non-Small-Cell Lung Cancer: A Randomized, Multicenter Phase II Trial. J. Clin. Oncol..

[75] Soria J.C., Tan D.S.W., Chiari R., Wu Y.L., Paz-Ares L., Wolf J., Geater S.L., Orlov S., Cortinovis D., Yu C.J. (2017). First-line ceritinib versus platinum-based chemotherapy in advanced ALK-rearranged non-small-cell lung cancer (ASCEND-4): A randomised, open-label, phase 3 study. Lancet.

[76] Patcas A., Chis A.F., Militaru C.F., Bordea I.R., Rajnoveanu R., Coza O.F., Hanna R., Tiberiu T., Todea D.A. (2022). An insight into lung cancer: A comprehensive review exploring ALK TKI and mechanisms of resistance. Bosn. J. Basic Med. Sci..

[77] Kwak E.L., Bang Y.J., Camidge D.R., Shaw A.T., Solomon B., Maki R.G., Ou S.H., Dezube B.J., Jänne P.A., Costa D.B. (2010). Anaplastic lymphoma kinase inhibition in non-small-cell lung cancer. N. Engl. J. Med..

[78] Kazandjian D., Blumenthal G.M., Chen H.Y., He K., Patel M., Justice R., Keegan P., Pazdur R. (2014). FDA approval summary: Crizotinib for the treatment of metastatic non-small cell lung cancer with anaplastic lymphoma kinase rearrangements. Oncologist.

[79] Wu Y.L., Lu S., Lu Y., Zhou J., Shi Y.K., Sriuranpong V., Ho J.C.M., Ong C.K., Tsai C.M., Chung C.H. (2018). Results of PROFILE 1029, a Phase III Comparison of First-Line Crizotinib versus Chemotherapy in East Asian Patients with ALK-Positive Advanced Non-Small Cell Lung Cancer. J. Thorac. Oncol..

[80] Dagogo-Jack I., Shaw A.T. (2016). Crizotinib resistance: Implications for therapeutic strategies. Ann. Oncol..

[81] Ling Z., Yunxia L., Shaohong Z., Chen G., Keke N., Youxin J. (2018). Primary resistance to crizotinib treatment in a non-small cell lung cancer patient with an EML4-ALK rearrangement: A case report. Cancer Biol. Med..

[82] Okada K., Araki M., Sakashita T., Ma B., Kanada R., Yanagitani N., Horiike A., Koike S., Oh-Hara T., Watanabe K. (2019). Prediction of ALK mutations mediating ALK-TKIs resistance and drug re-purposing to overcome the resistance. EBioMedicine.

[83] Gainor J.F., Dardaei L., Yoda S., Friboulet L., Leshchiner I., Katayama R., Dagogo-Jack I., Gadgeel S., Schultz K., Singh M. (2016). Molecular Mechanisms of Resistance to First- and Second-Generation ALK Inhibitors in ALK-Rearranged Lung Cancer. Cancer Discov..

[84] Katayama R., Shaw A.T., Khan T.M., Mino-Kenudson M., Solomon B.J., Halmos B., Jessop N.A., Wain J.C., Yeo A.T., Benes C. (2012). Mechanisms of acquired crizotinib resistance in ALK-rearranged lung Cancers. Sci. Transl. Med..

[85] Ando K., Akimoto K., Sato H., Manabe R., Kishino Y., Homma T., Kusumoto S., Yamaoka T., Tanaka A., Ohmori T. (2020). Brigatinib and Alectinib for ALK Rearrangement-Positive Advanced Non-Small Cell Lung Cancer With or Without Central Nervous System Metastasis: A Systematic Review and Network Meta-Analysis. Cancers.

[86] Hida T., Nokihara H., Kondo M., Kim Y.H., Azuma K., Seto T., Takiguchi Y., Nishio M., Yoshioka H., Imamura F. (2017). Alectinib versus crizotinib in patients with ALK-positive non-small-cell lung cancer (J-ALEX): An open-label, randomised phase 3 trial. Lancet.

[87] Nakagawa K., Hida T., Nokihara H., Morise M., Azuma K., Kim Y.H., Seto T., Takiguchi Y., Nishio M., Yoshioka H. (2020). Final progression-free survival results from the J-ALEX study of alectinib versus crizotinib in ALK-positive non-small-cell lung cancer. Lung Cancer.

[88] Reckamp K., Lin H.M., Huang J., Proskorovsky I., Reichmann W., Krotneva S., Kerstein D., Huang H., Lee J. (2019). Comparative efficacy of brigatinib versus ceritinib and alectinib in patients with crizotinib-refractory anaplastic lymphoma kinase-positive non-small cell lung cancer. Curr. Med. Res. Opin..

[89] Camidge D.R., Kim H.R., Ahn M.J., Yang J.C., Han J.Y., Lee J.S., Hochmair M.J., Li J.Y., Chang G.C., Lee K.H. (2018). Brigatinib versus Crizotinib in ALK-Positive Non-Small-Cell Lung Cancer. N. Engl. J. Med..

[90] Lin J.J., Zhu V.W., Yoda S., Yeap B.Y., Schrock A.B., Dagogo-Jack I., Jessop N.A., Jiang G.Y., Le L.P., Gowen K. (2018). Impact of EML4-ALK Variant on Resistance Mechanisms and Clinical Outcomes in ALK-Positive Lung Cancer. J. Clin. Oncol..

[91] Yoda S., Lin J.J., Lawrence M.S., Burke B.J., Friboulet L., Langenbucher A., Dardaei L., Prutisto-Chang K., Dagogo-Jack I., Timofeevski S. (2018). Sequential ALK Inhibitors Can Select for Lorlatinib-Resistant Compound ALK Mutations in ALK-Positive Lung Cancer. Cancer Discov..

[92] Solomon B.J., Besse B., Bauer T.M., Felip E., Soo R.A., Camidge D.R., Chiari R., Bearz A., Lin C.C., Gadgeel S.M. (2018). Lorlatinib in patients with ALK-positive non-small-cell lung cancer: Results from a global phase 2 study. Lancet Oncol..

[93] Chen J., Ruiz-Garcia A., James L.P., Peltz G., Thurm H., Clancy J., Hibma J. (2021). Lorlatinib Exposure-Response Analyses for Safety and Efficacy in a Phase I/II Trial to Support Benefit-Risk Assessment in Non-Small Cell Lung Cancer. Clin. Pharmacol. Ther..

[94] Shaw A.T., Bauer T.M., de Marinis F., Felip E., Goto Y., Liu G., Mazieres J., Kim D.W., Mok T., Polli A. (2020). First-Line Lorlatinib or Crizotinib in Advanced ALK-Positive Lung Cancer. N. Engl. J. Med..

[95] Gristina V., La Mantia M., Iacono F., Galvano A., Russo A., Bazan V. (2020). The Emerging Therapeutic Landscape of ALK Inhibitors in Non-Small Cell Lung Cancer. Pharmaceuticals.

[96] Sheikine Y., Pavlick D., Klempner S.J., Trabucco S.E., Chung J.H., Rosenzweig M., Wang K., Velcheti V., Frampton G.M., Peled N. (2018). BRAF in Lung Cancers: Analysis of Patient Cases Reveals Recurrent BRAF Mutations, Fusions, Kinase Duplications, and Concurrent Alterations. JCO Precis. Oncol..

[97] Dankner M., Rose A.A.N., Rajkumar S., Siegel P.M., Watson I.R. (2018). Classifying BRAF alterations in cancer: New rational therapeutic strategies for actionable mutations. Oncogene.

[98] Guo Y.J., Pan W.W., Liu S.B., Shen Z.F., Xu Y., Hu L.L. (2020). ERK/MAPK signalling pathway and tumorigenesis. Exp. Ther. Med..

[99] Degirmenci U., Wang M., Hu J. (2020). Targeting Aberrant RAS/RAF/MEK/ERK Signaling for Cancer Therapy. Cells.

[100] Candido S., Rapisarda V., Marconi A., Malaponte G., Bevelacqua V., Gangemi P., Scalisi A., McCubrey J.A., Maestro R., Spandidos D.A. (2014). Analysis of the B-RafV600E mutation in cutaneous melanoma patients with occupational sun exposure. Oncol. Rep..

[101] Nam S.K., Yun S., Koh J., Kwak Y., Seo A.N., Park K.U., Kim D.W., Kang S.B., Kim W.H., Lee H.S. (2016). BRAF, PIK3CA, and HER2 Oncogenic Alterations According to KRAS Mutation Status in Advanced Colorectal Cancers with Distant Metastasis. PLoS ONE.

[102] Nagayama Y., Mishima H. (2019). Heterogenous nature of gene expression patterns in BRAF-like papillary thyroid carcinomas with BRAFV600E. Endocrine.

[103] Villaruz L.C., Socinski M.A., Abberbock S., Berry L.D., Johnson B.E., Kwiatkowski D.J., Iafrate A.J., Varella-Garcia M., Franklin W.A., Camidge D.R. (2015). Clinicopathologic features and outcomes of patients with lung adenocarcinomas harboring BRAF mutations in the Lung Cancer Mutation Consortium. Cancer.

[104] Wan P.T., Garnett M.J., Roe S.M., Lee S., Niculescu-Duvaz D., Good V.M., Jones C.M., Marshall C.J., Springer C.J., Barford D. (2004). Mechanism of activation of the RAF-ERK signaling pathway by oncogenic mutations of B-RAF. Cell. Mar..

[105] Lin Q., Zhang H., Ding H., Qian J., Lizaso A., Lin J., Han-Zhang H., Xiang J., Li Y., Zhu H. (2019). The association between BRAF mutation class and clinical features in BRAF-mutant Chinese non-small cell lung cancer patients. J. Transl. Med..

[106] Planchard D., Kim T.M., Mazieres J., Quoix E., Riely G., Barlesi F., Souquet P.J., Smit E.F., Groen H.J., Kelly R.J. (2016). Dabrafenib in patients with BRAF(V600E)-positive advanced non-small-cell lung cancer: A single-arm, multicentre, open-label, phase 2 trial. Lancet Oncol..

[107] Litvak A.M., Paik P.K., Woo K.M., Sima C.S., Hellmann M.D., Arcila M.E., Ladanyi M., Rudin C.M., Kris M.G., Riely G.J. (2014). Clinical characteristics and course of 63 patients with BRAF mutant lung cancers. J. Thorac. Oncol..

[108] Ding X., Zhang Z., Jiang T., Li X., Zhao C., Su B., Zhou C. (2017). Clinicopathologic characteristics and outcomes of Chinese patients with non-small-cell lung cancer and BRAF mutation. Cancer Med..

[109] Weart T.C., Miller K.D., Simone C.B. (2018). Spotlight on dabrafenib/trametinib in the treatment of non-small-cell lung cancer: Place in therapy. Cancer Manag. Res..

[110] Hyman D.M., Puzanov I., Subbiah V., Faris J.E., Chau I., Blay J.Y., Wolf J., Raje N.S., Diamond E.L., Hollebecque A. (2015). Vemurafenib in Multiple Nonmelanoma Cancers with BRAF V600 Mutations. N. Engl. J. Med..

[111] Mazieres J., Cropet C., Montané L., Barlesi F., Souquet P.J., Quantin X., Dubos-Arvis C., Otto J., Favier L., Avrillon V. (2020). Vemurafenib in non-small-cell lung cancer patients with BRAFV600 and BRAFnonV600 mutations. Ann. Oncol..

[112] Khunger A., Khunger M., Velcheti V. (2018). Dabrafenib in combination with trametinib in the treatment of patients with BRAF V600-positive advanced or metastatic non-small cell lung cancer: Clinical evidence and experience. Ther. Adv. Respir. Dis..

[113] Planchard D., Smit E.F., Groen H.J.M., Mazieres J., Besse B., Helland Å., Giannone V., D’Amelio A.M., Zhang P., Mookerjee B. (2017). Dabrafenib plus trametinib in patients with previously untreated BRAFV600E-mutant metastatic non-small-cell lung cancer: An open-label, phase 2 trial. Lancet Oncol..

[114] Li J., Sasane M., Zhang J., Zhao J., Ricculli M.L., Yao Z., Redhu S., Signorovitch J. (2018). Is time to progression associated with post-progression survival in previously treated metastatic non-small cell lung cancer with BRAF V600E mutation? A secondary analysis of phase II clinical trial data. BMJ Open.

[115] Bustamante J.G., Otterson G.A. (2019). Agents to treat BRAF-mutant lung cancer. Drugs Context.

[116] Okimoto R.A., Lin L., Olivas V., Chan E., Markegard E., Rymar A., Neel D., Chen X., Hemmati G., Bollag G. (2016). Preclinical efficacy of a RAF inhibitor that evades paradoxical MAPK pathway activation in protein kinase BRAF-mutant lung cancer. Proc. Natl. Acad. Sci. USA.

[117] Miyauchi S., Shien K., Takeda T., Araki K., Nakata K., Miura A., Takahashi Y., Kurihara E., Ogoshi Y., Namba K. (2020). Antitumor Effects of Pan-RAF Inhibitor LY3009120 Against Lung Cancer Cells Harboring Oncogenic BRAF Mutation. Anticancer Res..

[118] Kohno T., Tabata J., Nakaoku T. (2020). REToma: A cancer subtype with a shared driver oncogene. Carcinogenesis.

[119] Wang R., Hu H., Pan Y., Li Y., Ye T., Li C., Luo X., Wang L., Li H., Zhang Y. (2012). RET fusions define a unique molecular and clinicopathologic subtype of non-small-cell lung cancer. J. Clin. Oncol..

[120] Drilon A., Lin J.J., Filleron T., Ni A., Milia J., Bergagnini I., Hatzoglou V., Velcheti V., Offin M., Li B. (2018). Frequency of Brain Metastases and Multikinase Inhibitor Outcomes in Patients With RET-Rearranged Lung Cancers. J. Thorac. Oncol..

[121] Ferrara R., Auger N., Auclin E., Besse B. (2018). Clinical and Translational Implications of RET Rearrangements in Non–Small Cell Lung Cancer. J. Thorac. Oncol..

[122] Du Z., Lovly C.M. (2018). Mechanisms of receptor tyrosine kinase activation in cancer. Mol. Cancer.

[123] Mendoza L. (2018). Clinical development of RET inhibitors in RET-rearranged non-small cell lung cancer: Update. Oncol. Rev..

[124] Drilon A., Rekhtman N., Arcila M., Wang L., Ni A., Albano M., Van Voorthuysen M., Somwar R., Smith R.S., Montecalvo J. (2016). Cabozantinib in patients with advanced RET-rearranged non-small-cell lung cancer: An open-label, single-centre, phase 2, single-arm trial. Lancet Oncol..

[125] Yoh K., Seto T., Satouchi M., Nishio M., Yamamoto N., Murakami H., Nogami N., Matsumoto S., Kohno T., Tsuta K. (2017). Vandetanib in patients with previously treated RET-rearranged advanced non-small-cell lung cancer (LURET): An open-label, multicentre phase 2 trial. Lancet Respir. Med..

[126] Hida T., Velcheti V., Reckamp K.L., Nokihara H., Sachdev P., Kubota T., Nakada T., Dutcus C.E., Ren M., Tamura T. (2019). A phase 2 study of lenvatinib in patients with RET fusion-positive lung adenocarcinoma. Lung Cancer.

[127] Bradford D., Larkins E., Mushti S.L., Rodriguez L., Skinner A.M., Helms W.S., Price L.S.L., Zirkelbach J.F., Li Y., Liu J. (2021). FDA Approval Summary: Selpercatinib for the Treatment of Lung and Thyroid Cancers with RET Gene Mutations or Fusions. Clin. Cancer Res..

[128] Markham A. (2020). Pralsetinib: First Approval. Drugs.

[129] Roskoski R. (2021). Properties of FDA-approved small molecule protein kinase inhibitors: A 2021 update. Pharmacol. Res..

[130] Drilon A., Oxnard G.R., Tan D.S.W., Loong H.H.F., Johnson M., Gainor J., McCoach C.E., Gautschi O., Besse B., Cho B.C. (2020). Efficacy of Selpercatinib in RET Fusion-Positive Non-Small-Cell Lung Cancer. N. Engl. J. Med..

[131] Gainor J.F., Curigliano G., Kim D.W., Lee D.H., Besse B., Baik C.S., Doebele R.C., Cassier P.A., Lopes G., Tan D.S.W. (2021). Pralsetinib for RET fusion-positive non-small-cell lung cancer (ARROW): A multi-cohort, open-label, phase 1/2 study. Lancet Oncol..

[132] Stinchcombe T.E. (2020). Current management of RET rearranged non-small cell lung cancer. Ther. Adv. Med. Oncol..

[133] Liu Y. (1998). The human hepatocyte growth factor receptor gene: Complete structural organization and promoter characterization. Gene.

[134] Skead G., Govender D. (2015). Gene of the month: MET. J. Clin. Pathol..

[135] Birchmeier C., Birchmeier W., Gherardi E., Vande Woude G.F. (2003). Met, metastasis, motility and more. Nat. Rev. Mol. Cell Biol..

[136] Cui J.J. (2014). Targeting Receptor Tyrosine Kinase MET in Cancer: Small Molecule Inhibitors and Clinical Progress. J. Med. Chem..

[137] Gelsomino F., Facchinetti F., Haspinger E.R., Garassino M.C., Trusolino L., De Braud F., Tiseo M. (2014). Targeting the MET gene for the treatment of non-small-cell lung cancer. Crit. Rev. Oncol. Hematol..

[138] Schrock A.B., Frampton G.M., Suh J., Chalmers Z.R., Rosenzweig M., Erlich R.L., Halmos B., Goldman J., Forde P., Leuenberger K. (2016). Characterization of 298 Patients with Lung Cancer Harboring MET Exon 14 Skipping Alterations. J. Thorac. Oncol..

[139] Frampton G.M., Ali S.M., Rosenzweig M., Chmielecki J., Lu X., Bauer T.M., Akimov M., Bufill J.A., Lee C., Jentz D. (2015). Activation of MET via diverse exon 14 splicing alterations occurs in multiple tumor types and confers clinical sensitivity to MET inhibitors. Cancer Discov..

[140] Guo B., Cen H., Tan X., Liu W., Ke Q. (2014). Prognostic Value of MET Gene Copy Number and Protein Expression in Patients with Surgically Resected Non-Small Cell Lung Cancer: A Meta-Analysis of Published Literatures. PLoS ONE.

[141] Pyo J.S., Kang G., Cho W.J., Choi S.B. (2016). Clinicopathological significance and concordance analysis of c-MET immunohistochemistry in non-small cell lung cancers: A meta-analysis. Pathol. Res. Pract..

[142] Reungwetwattana T., Liang Y., Zhu V., Ou S.H.I. (2017). The race to target MET exon 14 skipping alterations in non-small cell lung cancer: The Why, the How, the Who, the Unknown, and the Inevitable. Lung Cancer.

[143] Paik P.K., Drilon A., Fan P.D., Yu H., Rekhtman N., Ginsberg M.S., Borsu L., Schultz N., Berger M.F., Rudin C.M. (2015). Response to MET inhibitors in patients with stage IV lung adenocarcinomas harboring MET mutations causing exon 14 skipping. Cancer Discov..

[144] Cortot A.B., Kherrouche Z., Descarpentries C., Wislez M., Baldacci S., Furlan A., Tulasne D. (2017). Exon 14 Deleted MET Receptor as a New Biomarker and Target in Cancers. JNCI J. Natl. Cancer Inst..

[145] Salgia R., Sattler M., Scheele J., Stroh C., Felip E. (2020). The promise of selective MET inhibitors in non-small cell lung cancer with MET exon 14 skipping. Cancer Treat. Rev..

[146] Drilon A., Clark J.W., Weiss J., Ou S.I., Camidge D.R., Solomon B.J., Otterson G.A., Villaruz L.C., Riely G.J., Heist R.S. (2020). Antitumor activity of crizotinib in lung cancers harboring a MET exon 14 alteration. Nat. Med..

[147] Dhillon S. (2020). Capmatinib: First Approval. Drugs.

[148] Safi D., Abu Hejleh T., Furqan M. (2021). Narrative review: Mesenchymal-epithelial transition inhibitors-meeting their target. Transl. Lung Cancer Res..

[149] Wolf J., Seto T., Han J.Y., Reguart N., Garon E.B., Groen H.J.M., Tan D.S.W., Hida T., de Jonge M., Orlov S.V. (2020). Capmatinib in MET Exon 14-Mutated or MET-Amplified Non-Small-Cell Lung Cancer. N. Engl. J. Med..

[150] Paik P.K., Felip E., Veillon R., Sakai H., Cortot A.B., Garassino M.C., Mazieres J., Viteri S., Senellart H., Van Meerbeeck J. (2020). Tepotinib in Non-Small-Cell Lung Cancer with MET Exon 14 Skipping Mutations. N. Engl. J. Med..

[151] Cocco E., Scaltriti M., Drilon A. (2018). NTRK fusion-positive cancers and TRK inhibitor therapy. Nat. Rev. Clin. Oncol..

[152] Davies A., Horton A., Burton L., Schmelzer C., Vandlen R., Rosenthal A. (1993). Neurotrophin-4/5 is a mammalian-specific survival factor for distinct populations of sensory neurons. J. Neurosci..

[153] Harada T., Yatabe Y., Takeshita M., Koga T., Yano T., Wang Y., Giaccone G. (2011). Role and Relevance of TrkB Mutations and Expression in Non–Small Cell Lung Cancer. Clin. Cancer Res..

[154] Marchetti A., Felicioni L., Pelosi G., Del Grammastro M., Fumagalli C., Sciarrotta M., Malatesta S., Chella A., Barassi F., Mucilli F. (2008). Frequent mutations in the neurotrophic tyrosine receptor kinase gene family in large cell neuroendocrine carcinoma of the lung. Hum. Mutat..

[155] Dunn D.B. (2020). Larotrectinib and Entrectinib: TRK Inhibitors for the Treatment of Pediatric and Adult Patients With NTRK Gene Fusion. J. Adv. Pract. Oncol..

[156] Drilon A., Laetsch T.W., Kummar S., DuBois S.G., Lassen U.N., Demetri G.D., Nathenson M., Doebele R.C., Farago A.F., Pappo A.S. (2018). Efficacy of Larotrectinib in TRK Fusion-Positive Cancers in Adults and Children. N. Engl. J. Med..

[157] Hong D.S., DuBois S.G., Kummar S., Farago A.F., Albert C.M., Rohrberg K.S., van Tilburg C.M., Nagasubramanian R., Berlin J.D., Federman N. (2020). Larotrectinib in patients with TRK fusion-positive solid tumours: A pooled analysis of three phase 1/2 clinical trials. Lancet Oncol..

[158] Filippi R., Depetris I., Satolli M.A. (2021). Evaluating larotrectinib for the treatment of advanced solid tumors harboring an NTRK gene fusion. Expert Opin. Pharmacother..

[159] Doebele R.C., Drilon A., Paz-Ares L., Siena S., Shaw A.T., Farago A.F., Blakely C.M., Seto T., Cho B.C., Tosi D. (2020). Entrectinib in patients with advanced or metastatic NTRK fusion-positive solid tumours: Integrated analysis of three phase 1-2 trials. Lancet Oncol..

[160] Fuse M.J., Okada K., Oh-hara T., Ogura H., Fujita N., Katayama R. (2017). Mechanisms of Resistance to NTRK Inhibitors and Therapeutic Strategies in NTRK1-Rearranged Cancers. Mol. Cancer Ther..

[161] Somwar R., Hofmann N.E., Smith B., Odintsov I., Vojnic M., Linkov I., Tam A., Khodos I., Mattar M.S., de Stanchina E. (2020). NTRK kinase domain mutations in cancer variably impact sensitivity to type I and type II inhibitors. Commun. Biol..

[162] Guin S., Theodorescu D. (2015). The RAS-RAL axis in cancer: Evidence for mutation-specific selectivity in non-small cell lung cancer. Acta Pharmacol. Sin..

[163] Gupta S., Ramjaun A.R., Haiko P., Wang Y., Warne P.H., Nicke B., Nye E., Stamp G., Alitalo K., Downward J. (2007). Binding of ras to phosphoinositide 3-kinase p110alpha is required for ras-driven tumorigenesis in mice. Cell.

[164] Stokoe D., Macdonald S.G., Cadwallader K., Symons M., Hancock J.F. (1994). Activation of Raf as a result of recruitment to the plasma membrane. Science.

[165] Buscail L., Bournet B., Cordelier P. (2020). Role of oncogenic KRAS in the diagnosis, prognosis and treatment of pancreatic cancer. Nat. Rev. Gastroenterol. Hepatol..

[166] Brandt R., Sell T., Lüthen M., Uhlitz F., Klinger B., Riemer P., Giesecke-Thiel C., Schulze S., El-Shimy I.A., Kunkel D. (2019). Cell type-dependent differential activation of ERK by oncogenic KRAS in colon cancer and intestinal epithelium. Nat. Commun..

[167] Garrido P., Olmedo M.E., Gómez A., Paz Ares L., López-Ríos F., Rosa-Rosa J.M., Palacios J. (2017). Treating KRAS-mutant NSCLC: Latest evidence and clinical consequences. Ther. Adv. Med. Oncol..

[168] Kempf E., Rousseau B., Besse B., Paz-Ares L. (2016). KRAS oncogene in lung cancer: Focus on molecularly driven clinical trials. Eur. Respir. Rev..

[169] Westcott P.M.K., To M.D. (2013). The genetics and biology of KRAS in lung cancer. Chin. J. Cancer.

[170] Feng H., Zhang Y., Bos P.H., Chambers J.M., Dupont M.M., Stockwell B.R. (2019). K-Ras G12D Has a Potential Allosteric Small Molecule Binding Site. Biochemistry.

[171] Muñoz-Maldonado C., Zimmer Y., Medová M. (2019). A Comparative Analysis of Individual RAS Mutations in Cancer Biology. Front. Oncol..

[172] Skoulidis F., Byers L.A., Diao L., Papadimitrakopoulou V.A., Tong P., Izzo J., Behrens C., Kadara H., Parra E.R., Canales J.R. (2015). Co-occurring genomic alterations define major subsets of KRAS-mutant lung adenocarcinoma with distinct biology, immune profiles, and therapeutic vulnerabilities. Cancer Discov..

[173] Ferrer I., Zugazagoitia J., Herbertz S., John W., Paz-Ares L., Schmid-Bindert G. (2018). KRAS-Mutant non-small cell lung cancer: From biology to therapy. Lung Cancer.

[174] Veluswamy R., Mack P.C., Houldsworth J., Elkhouly E., Hirsch F.R. (2021). KRAS G12C–Mutant Non–Small Cell Lung Cancer. J. Mol. Diagn..

[175] Saleh K., Kordahi M., Felefly T., Kourie H.R., Khalife N. (2021). KRAS-targeted therapies in advanced solid cancers: Drug the undruggable?. Pharmacogenomics.

[176] Skoulidis F., Li B.T., Dy G.K., Price T.J., Falchook G.S., Wolf J., Italiano A., Schuler M., Borghaei H., Barlesi F. (2021). Sotorasib for Lung Cancers with KRAS p.G12C Mutation. N. Engl. J. Med..

[177] Jänne P.A., Rybkin I.I., Spira A.I., Riely G.J., Papadopoulos K.P., Sabari J.K., Johnson M.L., Heist R.S., Bazhenova L., Barve M. (2020). KRYSTAL-1: Activity and Safety of Adagrasib (MRTX849) in Advanced/ Metastatic Non–Small-Cell Lung Cancer (NSCLC) Harboring KRAS G12C Mutation. Eur. J. Cancer.

[178] Hong D.S., Fakih M.G., Strickler J.H., Desai J., Durm G.A., Shapiro G.I., Falchook G.S., Price T.J., Sacher A., Denlinger C.S. (2020). KRASG12C Inhibition with Sotorasib in Advanced Solid Tumors. N. Engl. J. Med..

[179] D’Angelo A., Sobhani N., Chapman R., Bagby S., Bortoletti C., Traversini M., Ferrari K., Voltolini L., Darlow J., Roviello G. (2020). Focus on ROS1-Positive Non-Small Cell Lung Cancer (NSCLC): Crizotinib, Resistance Mechanisms and the Newer Generation of Targeted Therapies. Cancers.

[180] Yoshida A., Kohno T., Tsuta K., Wakai S., Arai Y., Shimada Y., Asamura H., Furuta K., Shibata T., Tsuda H. (2013). ROS1-rearranged lung cancer: A clinicopathologic and molecular study of 15 surgical cases. Am. J. Surg. Pathol..

[181] Davies K.D., Doebele R.C. (2013). Molecular Pathways: ROS1 Fusion Proteins in Cancer. Clin. Cancer Res..

[182] Davies K.D., Le A.T., Theodoro M.F., Skokan M.C., Aisner D.L., Berge E.M., Terracciano L.M., Cappuzzo F., Incarbone M., Roncalli M. (2012). Identifying and targeting ROS1 gene fusions in non-small cell lung cancer. Clin. Cancer Res..

[183] Lin J.J., Shaw A.T. (2017). Recent Advances in Targeting ROS1 in Lung Cancer. J. Thorac. Oncol..

[184] Shaw A.T., Ou S.H., Bang Y.J., Camidge D.R., Solomon B.J., Salgia R., Riely G.J., Varella-Garcia M., Shapiro G.I., Costa D.B. (2014). Crizotinib in ROS1-rearranged non-small-cell lung cancer. N. Engl. J. Med..

[185] Moro-Sibilot D., Cozic N., Pérol M., Mazières J., Otto J., Souquet P.J., Bahleda R., Wislez M., Zalcman G., Guibert S.D. (2019). Crizotinib in c-MET- or ROS1-positive NSCLC: Results of the AcSé phase II trial. Ann. Oncol..

[186] Lim S.M., Kim H.R., Lee J.S., Lee K.H., Lee Y.G., Min Y.J., Cho E.K., Lee S.S., Kim B.S., Choi M.Y. (2017). Open-Label, Multicenter, Phase II Study of Ceritinib in Patients With Non-Small-Cell Lung Cancer Harboring ROS1 Rearrangement. J. Clin. Oncol..

[187] Drilon A., Siena S., Dziadziuszko R., Barlesi F., Krebs M.G., Shaw A.T., de Braud F., Rolfo C., Ahn M.J., Wolf J. (2020). Entrectinib in ROS1 fusion-positive non-small-cell lung cancer: Integrated analysis of three phase 1-2 trials. Lancet Oncol..

[188] Davare M.A., Vellore N.A., Wagner J.P., Eide C.A., Goodman J.R., Drilon A., Deininger M.W., O’Hare T., Druker B.J. (2015). Structural insight into selectivity and resistance profiles of ROS1 tyrosine kinase inhibitors. Proc. Natl. Acad. Sci. USA.

[189] Pathak D., Choudhary S., Singh P.K., Singh M., Chadha N., Silakari O. (2021). Pharmacophore-based designing of putative ROS-1 targeting agents for NSCLC. Mol. Divers..

[190] Vanajothi R., Vedagiri H., Al-Ansari M.M., Al-Humaid L.A., Kumpati P. (2020). Pharmacophore based virtual screening, molecular docking and molecular dynamic simulation studies for finding ROS1 kinase inhibitors as potential drug molecules. J. Biomol. Struct. Dyn..

[191] Pathak D., Chadha N., Silakari O. (2016). Identification of non-resistant ROS-1 inhibitors using structure-based pharmacophore analysis. J. Mol. Graph. Model..

[192] Zou H.Y., Li Q., Engstrom L.D., West M., Appleman V., Wong K.A., McTigue M., Deng Y.L., Liu W., Brooun A. (2015). PF-06463922 is a potent and selective next-generation ROS1/ALK inhibitor capable of blocking crizotinib-resistant ROS1 mutations. Proc. Natl. Acad. Sci. USA.

[193] Ou S.I., Fujiwara Y., Shaw A.T., Yamamoto N., Nakagawa K., Fan F., Hao Y., Gao Y., Jänne P.A., Seto T. (2020). Efficacy of Taletrectinib (AB-106/DS-6051b) in ROS1+ NSCLC: An Updated Pooled Analysis of U.S. and Japan Phase 1 Studies. JTO Clin. Res. Rep..

[194] Karakas B., Bachman K.E., Park B.H. (2006). Mutation of the PIK3CA oncogene in human cancers. Br. J. Cancer.

[195] Reddy D., Ghosh P., Kumavath R. (2020). Strophanthidin Attenuates MAPK, PI3K/AKT/mTOR, and Wnt/β-Catenin Signaling Pathways in Human Cancers. Front. Oncol..

[196] Lu H., Qin J., Han N., Lei L., Xie F., Li C. (2018). EGFR, KRAS, BRAF, PTEN, and PIK3CA mutation in plasma of small cell lung cancer patients. Onco Targets Ther..

[197] Scheffler M., Bos M., Gardizi M., König K., Michels S., Fassunke J., Heydt C., Künstlinger H., Ihle M., Ueckeroth F. (2015). PIK3CA mutations in non-small cell lung cancer (NSCLC): Genetic heterogeneity, prognostic impact and incidence of prior malignancies. Oncotarget.

[198] Li S., Li L., Zhu Y., Huang C., Qin Y., Liu H., Ren-Heidenreich L., Shi B., Ren H., Chu X. (2014). Coexistence of EGFR with KRAS, or BRAF, or PIK3CA somatic mutations in lung cancer: A comprehensive mutation profiling from 5125 Chinese cohorts. Br. J. Cancer.

[199] Vansteenkiste J.F., Canon J.L., De Braud F., Grossi F., De Pas T., Gray J.E., Su W.C., Felip E., Yoshioka H., Gridelli C. (2015). Safety and Efficacy of Buparlisib (BKM120) in Patients with PI3K Pathway-Activated Non-Small Cell Lung Cancer: Results from the Phase II BASALT-1 Study. J. Thorac. Oncol..

[200] Lopez-Chavez A., Thomas A., Rajan A., Raffeld M., Morrow B., Kelly R., Carter C.A., Guha U., Killian K., Lau C.C. (2015). Molecular profiling and targeted therapy for advanced thoracic malignancies: A biomarker-derived, multiarm, multihistology phase II basket trial. J. Clin. Oncol..

[201] Langer C.J., Redman M.W., Wade J.L., Aggarwal C., Bradley J.D., Crawford. J., Stella P.J., Knapp M.H., Miao J., Minichiello K. (2019). SWOG S1400B (NCT02785913), a Phase II Study of GDC-0032 (Taselisib) for Previously Treated PI3K-Positive Patients with Stage IV Squamous Cell Lung Cancer (Lung-MAP Sub-Study). J. Thorac. Oncol..

[202] Galluzzi L., Chan T.A., Kroemer G., Wolchok J.D., López-Soto A. (2018). The hallmarks of successful anticancer immunotherapy. Sci. Transl. Med..

[203] Qin S.S., Melucci A.D., Chacon A.C., Prieto P.A. (2021). Adoptive T Cell Therapy for Solid Tumors: Pathway to Personalized Standard of Care. Cells.

[204] Waldman A.D., Fritz J.M., Lenardo M.J. (2020). A guide to cancer immunotherapy: From T cell basic science to clinical practice. Nat. Rev. Immunol..

[205] Jung N.C., Lee J.H., Chung K.H., Kwak Y.S., Lim D.S. (2018). Dendritic Cell-Based Immunotherapy for Solid Tumors. Transl. Oncol..

[206] Liu Z., Han C., Fu Y.X. (2020). Targeting innate sensing in the tumor microenvironment to improve immunotherapy. Cell. Mol. Immunol..

[207] Shin E.C. (2021). Cancer immunotherapy: Special issue of BMB Reports in 2021. BMB Rep..

[208] Pardoll D.M. (2012). The blockade of immune checkpoints in cancer immunotherapy. Nat. Rev. Cancer.

[209] Dong Y., Sun Q., Zhang X. (2017). PD-1 and its ligands are important immune checkpoints in cancer. Oncotarget.

[210] Shen X., Zhang L., Li J., Li Y., Wang Y., Xu Z.X. (2019). Recent Findings in the Regulation of Programmed Death Ligand 1 Expression. Front. Immunol..

[211] Wu Y., Chen W., Xu Z.P., Gu W. (2019). PD-L1 Distribution and Perspective for Cancer Immunotherapy-Blockade, Knockdown, or Inhibition. Front. Immunol..

[212] Vaddepally R.K., Kharel P., Pandey R., Garje R., Chandra A.B. (2020). Review of Indications of FDA-Approved Immune Checkpoint Inhibitors per NCCN Guidelines with the Level of Evidence. Cancers.

[213] Zhou X., Yao Z., Yang H., Liang N., Zhang X., Zhang F. (2020). Are immune-related adverse events associated with the efficacy of immune checkpoint inhibitors in patients with cancer? A systematic review and meta-analysis. BMC Med..

[214] Twomey J.D., Zhang B. (2021). Cancer Immunotherapy Update: FDA-Approved Checkpoint Inhibitors and Companion Diagnostics. AAPS J..

[215] Zhou F., Qiao M., Zhou C. (2021). The cutting-edge progress of immune-checkpoint blockade in lung cancer. Cell. Mol. Immunol..

[216] Teixidor E., Bosch-Barrera J. (2019). The dark side of immunotherapy: Challenges facing the new hope in cancer treatment. Ann. Transl. Med..

[217] Kim J.M., Chen D.S. (2016). Immune escape to PD-L1/PD-1 blockade: Seven steps to success (or failure). Ann. Oncol..

[218] Wang D., Chen C., Gu Y., Lu W., Zhan P., Liu H., Lv T., Song Y., Zhang F. (2021). Immune-Related Adverse Events Predict the Efficacy of Immune Checkpoint Inhibitors in Lung Cancer Patients: A Meta-Analysis. Front. Oncol..

[219] Hodi F.S., O’Day S.J., McDermott D.F., Weber R.W., Sosman J.A., Haanen J.B., Gonzalez R., Robert C., Schadendorf D., Hassel J.C. (2010). Improved survival with ipilimumab in patients with metastatic melanoma. N. Engl. J. Med..

[220] Ibarrondo F.J. (2016). Comin-Anduix B and Escuin-Ordinas H: Tremelimumab: Research and clinical development. Onco Targets Ther..

[221] Buchbinder E.I., Desai A. (2016). CTLA-4 and PD-1 Pathways: Similarities, Differences, and Implications of Their Inhibition. Am. J. Clin. Oncol..

[222] Lynch T.J., Bondarenko I., Luft A., Serwatowski P., Barlesi F., Chacko R., Sebastian M., Neal J., Lu H., Cuillerot J.M. (2012). Ipilimumab in combination with paclitaxel and carboplatin as first-line treatment in stage IIIB/IV non-small-cell lung cancer: Results from a randomized, double-blind, multicenter phase II study. J. Clin. Oncol..

[223] Reck M., Bondarenko I., Luft A., Serwatowski P., Barlesi F., Chacko R., Sebastian M., Lu H., Cuillerot J.M., Lynch T.J. (2013). Ipilimumab in combination with paclitaxel and carboplatin as first-line therapy in extensive-disease-small-cell lung cancer: Results from a randomized, double-blind, multicenter phase 2 trial. Ann. Oncol..

[224] Arriola E., Wheater M., Galea I., Cross N., Maishman T., Hamid D., Stanton L., Cave J., Geldart T., Mulatero C. (2016). Outcome and Biomarker Analysis from a Multicenter Phase 2 Study of Ipilimumab in Combination with Carboplatin and Etoposide as First-Line Therapy for Extensive-Stage SCLC. J. Thorac. Oncol..

[225] Reck M., Luft A., Szczesna A., Havel L., Kim S.W., Akerley W., Pietanza M.C., Wu Y.L., Zielinski C., Thomas M. (2016). Phase III Randomized Trial of Ipilimumab Plus Etoposide and Platinum Versus Placebo Plus Etoposide and Platinum in Extensive-Stage Small-Cell Lung Cancer. J. Clin. Oncol..

[226] Fehrenbacher L., Spira A., Ballinger M., Kowanetz M., Vansteenkiste J., Mazieres J., Park K., Smith D., Artal-Cortes A., Lewanski C. (2016). Atezolizumab versus docetaxel for patients with previously treated non-small-cell lung cancer (POPLAR): A multicentre, open-label, phase 2 randomised controlled trial. Lancet.

[227] Borghaei H., Paz-Ares L., Horn L., Spigel D.R., Steins M., Ready N.E., Chow L.Q., Vokes E.E., Felip E., Holgado E. (2015). Nivolumab versus Docetaxel in Advanced Nonsquamous Non-Small-Cell Lung Cancer. N. Engl. J. Med..

[228] Garon E.B., Rizvi N.A., Hui R., Leighl N., Balmanoukian A.S., Eder J.P., Patnaik A., Aggarwal C., Gubens M., Horn L. (2015). Pembrolizumab for the treatment of non-small-cell lung cancer. N. Engl. J. Med..

[229] Hotta K., Nishio M., Saito H., Okamoto I., Nakahara Y., Hayashi H., Hayama M., Laud P., Jiang H., Paz-Ares L. (2021). First-line durvalumab plus platinum-etoposide in extensive-stage small-cell lung cancer: CASPIAN Japan subgroup analysis. Int. J. Clin. Oncol..

[230] Brahmer J., Reckamp K.L., Baas P., Crinò L., Eberhardt W.E., Poddubskaya E., Antonia S., Pluzanski A., Vokes E.E., Holgado E. (2015). Nivolumab versus Docetaxel in Advanced Squamous-Cell Non-Small-Cell Lung Cancer. N. Engl. J. Med..

[231] Vokes E.E., Ready N., Felip E., Horn L., Burgio M.A., Antonia S.J., Arén Frontera O., Gettinger S., Holgado E., Spigel D. (2018). Nivolumab versus docetaxel in previously treated advanced non-small-cell lung cancer (CheckMate 017 and CheckMate 057): 3-year update and outcomes in patients with liver metastases. Ann. Oncol..

[232] Wu Y.L., Lu S., Cheng Y., Zhou C., Wang J., Mok T., Zhang L., Tu H.Y., Wu L., Feng J. (2019). Nivolumab Versus Docetaxel in a Predominantly Chinese Patient Population With Previously Treated Advanced NSCLC: CheckMate 078 Randomized Phase III Clinical Trial. J. Thorac. Oncol..

[233] Pai-Scherf L., Blumenthal G.M., Li H., Subramaniam S., Mishra-Kalyani P.S., He K., Zhao H., Yu J., Paciga M., Goldberg K.B. (2017). FDA Approval Summary: Pembrolizumab for Treatment of Metastatic Non-Small Cell Lung Cancer: First-Line Therapy and Beyond. Oncologist.

[234] Mok T.S.K., Wu Y.L., Kudaba I., Kowalski D.M., Cho B.C., Turna H.Z., Castro G., Srimuninnimit V., Laktionov K.K., Bondarenko I. (2019). Pembrolizumab versus chemotherapy for previously untreated, PD-L1-expressing, locally advanced or metastatic non-small-cell lung cancer (KEYNOTE-042): A randomised, open-label, controlled, phase 3 trial. Lancet.

[235] Borghaei H., Langer C.J., Paz-Ares L., Rodríguez-Abreu D., Halmos B., Garassino M.C., Houghton B., Kurata T., Cheng Y., Lin J. (2020). Pembrolizumab plus chemotherapy versus chemotherapy alone in patients with advanced non-small cell lung cancer without tumor PD-L1 expression: A pooled analysis of 3 randomized controlled trials. Cancer.

[236] Theelen W.S.M.E., Peulen H.M.U., Lalezari F., van der Noort V., de Vries J.F., Aerts J.G.J.V., Dumoulin D.W., Bahce I., Niemeijer A.N., de Langen A.J. (2019). Effect of Pembrolizumab After Stereotactic Body Radiotherapy vs Pembrolizumab Alone on Tumor Response in Patients With Advanced Non-Small Cell Lung Cancer: Results of the PEMBRO-RT Phase 2 Randomized Clinical Trial. JAMA Oncol..

[237] Welsh J., Menon H., Chen D., Verma V., Tang C., Altan M., Hess K., de Groot P., Nguyen Q.N., Varghese R. (2020). Pembrolizumab with or without radiation therapy for metastatic non-small cell lung cancer: A randomized phase I/II trial. J. Immunother Cancer.

[238] Theelen W.S.M.E., Chen D., Verma V., Hobbs B.P., Peulen H.M.U., Aerts J.G.J.V., Bahce I., Niemeijer A.L.N., Chang J.Y., de Groot P.M. (2021). Pembrolizumab with or without radiotherapy for metastatic non-small-cell lung cancer: A pooled analysis of two randomised trials. Lancet Respir. Med..

[239] Krishnamurthy A., Jimeno A. (2017). Atezolizumab: A novel PD-L1 inhibitor in cancer therapy with a focus in bladder and non-small cell lung cancers. Drugs Today.

[240] Rittmeyer A., Barlesi F., Waterkamp D., Park K., Ciardiello F., von Pawel J., Gadgeel S.M., Hida T., Kowalski D.M., Dols M.C. (2017). Atezolizumab versus docetaxel in patients with previously treated non-small-cell lung cancer (OAK): A phase 3, open-label, multicentre randomised controlled trial. Lancet.

[241] Horn L., Mansfield A.S., Szczęsna A., Havel L., Krzakowski M., Hochmair M.J., Huemer F., Losonczy G., Johnson M.L., Nishio M. (2018). First-Line Atezolizumab plus Chemotherapy in Extensive-Stage Small-Cell Lung Cancer. N. Engl. J. Med..

[242] Antonia S.J., Villegas A., Daniel D., Vicente D., Murakami S., Hui R., Kurata T., Chiappori A., Lee K.H., de Wit M. (2018). Overall Survival with Durvalumab after Chemoradiotherapy in Stage III NSCLC. N. Engl. J. Med..

[243] Faivre-Finn C., Vicente D., Kurata T., Planchard D., Paz-Ares L., Vansteenkiste J.F., Spigel D.R., Garassino M.C., Reck M., Senan S. (2021). Four-Year Survival with Durvalumab After Chemoradiotherapy in Stage III NSCLC—An Update From the PACIFIC Trial. J. Thorac. Oncol..

[244] Paz-Ares L., Dvorkin M., Chen Y., Reinmuth N., Hotta K., Trukhin D., Statsenko G., Hochmair M.J., Özgüroğlu M., Ji J.H. (2019). Durvalumab plus platinum-etoposide versus platinum-etoposide in first-line treatment of extensive-stage small-cell lung cancer (CASPIAN): A randomised, controlled, open-label, phase 3 trial. Lancet.

[245] Gray J.E., Villegas A., Daniel D., Vicente D., Murakami S., Hui R., Kurata T., Chiappori A., Lee K.H., Cho B.C. (2020). Three-Year Overall Survival with Durvalumab after Chemoradiotherapy in Stage III NSCLC—Update from PACIFIC. J. Thorac. Oncol..

[246] Barlesi F., Vansteenkiste J., Spigel D., Ishii H., Garassino M., de Marinis F., Özgüroğlu M., Szczesna A., Polychronis A., Uslu R. (2018). Avelumab versus docetaxel in patients with platinum-treated advanced non-small-cell lung cancer (JAVELIN Lung 200): An open-label, randomised, phase 3 study. Lancet. Oncol..

[247] Pabla S., Conroy J.M., Nesline M.K., Glenn S.T., Papanicolau-Sengos A., Burgher B., Hagen J., Giamo V., Andreas J., Lenzo F.L. (2019). Proliferative potential and resistance to immune checkpoint blockade in lung cancer patients. J. Immunother. Cancer.

[248] Fares C.M., Van Allen E.M., Drake C.G., Allison J.P., Hu-Lieskovan S. (2019). Mechanisms of Resistance to Immune Checkpoint Blockade: Why Does Checkpoint Inhibitor Immunotherapy Not Work for All Patients?. Am. Soc. Clin. Oncol. Educ. Book.

[249] Ready N.E., Ott P.A., Hellmann M.D., Zugazagoitia J., Hann C.L., de Braud F., Antonia S.J., Ascierto P.A., Moreno V., Atmaca A. (2020). Nivolumab Monotherapy and Nivolumab Plus Ipilimumab in Recurrent Small Cell Lung Cancer: Results From the CheckMate 032 Randomized Cohort. J. Thorac. Oncol..

[250] Paz-Ares L., Ciuleanu T.E., Cobo M., Schenker M., Zurawski B., Menezes J., Richardet E., Bennouna J., Felip E., Juan-Vidal O. (2021). First-line nivolumab plus ipilimumab combined with two cycles of chemotherapy in patients with non-small-cell lung cancer (CheckMate 9LA): An international, randomised, open-label, phase 3 trial. Lancet Oncol..

[251] Boyer M., Şendur M.A.N., Rodríguez-Abreu D., Park K., Lee D.H., Çiçin I., Yumuk P.F., Orlandi F.J., Leal T.A., Molinier O. (2021). Pembrolizumab Plus Ipilimumab or Placebo for Metastatic Non-Small-Cell Lung Cancer With PD-L1 Tumor Proportion Score ≥ 50%: Randomized, Double-Blind Phase III KEYNOTE-598 Study. J. Clin. Oncol..

[252] Planchard D., Reinmuth N., Orlov S., Fischer J.R., Sugawara S., Mandziuk S., Marquez-Medina D., Novello S., Takeda Y., Soo R. (2020). ARCTIC: Durvalumab with or without tremelimumab as third-line or later treatment of metastatic non-small-cell lung cancer. Ann. Oncol..

[253] Rizvi N.A., Cho B.C., Reinmuth N., Lee K.H., Luft A., Ahn M.J., van den Heuvel M.M., Cobo M., Vicente D., Smolin A. (2020). Durvalumab With or Without Tremelimumab vs Standard Chemotherapy in First-line Treatment of Metastatic Non-Small Cell Lung Cancer: The MYSTIC Phase 3 Randomized Clinical Trial. JAMA Oncol..

[254] Onoi K., Chihara Y., Uchino J., Shimamoto T., Morimoto Y., Iwasaku M., Kaneko Y., Yamada T., Takayama K. (2020). Immune Checkpoint Inhibitors for Lung Cancer Treatment: A Review. J. Clin. Med..

[255] Lei Y., Li X., Huang Q., Zheng X., Liu M. (2021). Progress and Challenges of Predictive Biomarkers for Immune Checkpoint Blockade. Front. Oncol..

[256] Bai R., Lv Z., Xu D., Cui J. (2020). Predictive biomarkers for cancer immunotherapy with immune checkpoint inhibitors. Biomark. Res..

[257] Havel J.J., Chowell D., Chan T.A. (2019). The evolving landscape of biomarkers for checkpoint inhibitor immunotherapy. Nat. Rev. Cancer.

[258] Herbst R.S., Baas P., Kim D.W., Felip E., Pérez-Gracia J.L., Han J.Y., Molina J., Kim J.H., Arvis C.D., Ahn M.J. (2016). Pembrolizumab versus docetaxel for previously treated, PD-L1-positive, advanced non-small-cell lung cancer (KEYNOTE-010): A randomised controlled trial. Lancet.

[259] Reck M., Rodríguez-Abreu D., Robinson A.G., Hui R., Csőszi T., Fülöp A., Gottfried M., Peled N., Tafreshi A., Cuffe S. (2016). Pembrolizumab versus Chemotherapy for PD-L1-Positive Non-Small-Cell Lung Cancer. N. Engl. J. Med..

[260] Hong L., Negrao M.V., Dibaj S.S., Chen R., Reuben A., Bohac J.M., Liu X., Skoulidis F., Gay C.M., Cascone T. (2020). Programmed Death-Ligand 1 Heterogeneity and Its Impact on Benefit From Immune Checkpoint Inhibitors in NSCLC. J. Thorac. Oncol..

[261] Gaule P., Smithy J.W., Toki M., Rehman J., Patell-Socha F., Cougot D., Collin P., Morrill P., Neumeister V., Rimm D.L. (2017). A Quantitative Comparison of Antibodies to Programmed Cell Death 1 Ligand 1. JAMA Oncol..

[262] Greillier L., Tomasini P., Barlesi F. (2018). The clinical utility of tumor mutational burden in non-small cell lung cancer. Transl. Lung Cancer Res..

[263] Wang Z., Zhao J., Wang G., Zhang F., Zhang Z., Zhang F., Zhang Y., Dong H., Zhao X., Duan J. (2018). Comutations in DNA Damage Response Pathways Serve as Potential Biomarkers for Immune Checkpoint Blockade. Cancer Res..

[264] Gandara D.R., Paul S.M., Kowanetz M., Schleifman E., Zou W., Li Y., Rittmeyer A., Fehrenbacher L., Otto G., Malboeuf C. (2018). Blood-based tumor mutational burden as a predictor of clinical benefit in non-small-cell lung cancer patients treated with atezolizumab. Nat. Med..

[265] Garassino M., Rodriguez-Abreu D., Gadgeel S., Esteban E., Felip E., Speranza G., Reck M., Hui R., Boyer M., Cristescu R. (2019). OA04.06 Evaluation of TMB in KEYNOTE-189: Pembrolizumab Plus Chemotherapy vs Placebo Plus Chemotherapy for Nonsquamous NSCLC. J. Thorac. Oncol..

[266] Rackaityte E., Lynch S.V. (2020). The human microbiome in the 21st century. Nat. Commun..

[267] Carbone C., Piro G., Di Noia V., D’Argento E., Vita E., Ferrara M.G., Pilotto S., Milella M., Cammarota G., Gasbarrini A. (2019). Lung and Gut Microbiota as Potential Hidden Driver of Immunotherapy Efficacy in Lung Cancer. Mediat. Inflamm..

[268] Vivarelli S., Salemi R., Candido S., Falzone L., Santagati M., Stefani S., Torino F., Banna G.L., Tonini G., Libra M. (2019). Gut Microbiota and Cancer: From Pathogenesis to Therapy. Cancers.

[269] Martins D., Mendes F., Schmitt F. (2021). Microbiome: A Supportive or a Leading Actor in Lung Cancer?. Pathobiology.

[270] Baruch E.N., Youngster I., Ben-Betzalel G., Ortenberg R., Lahat A., Katz L., Adler K., Dick-Necula D., Raskin S., Bloch N. (2021). Fecal microbiota transplant promotes response in immunotherapy-refractory melanoma patients. Science.

[271] Davar D., Dzutsev A.K., McCulloch J.A., Rodrigues R.R., Chauvin J.M., Morrison R.M., Deblasio R.N., Menna C., Ding Q., Pagliano O. (2021). Fecal microbiota transplant overcomes resistance to anti-PD-1 therapy in melanoma patients. Science.

[272] Vivarelli S., Falzone L., Basile M., Nicolosi D., Genovese C., Libra M., Salmeri M. (2019). Benefits of using probiotics as adjuvants in anticancer therapy (Review). World Acad. Sci. J..

[273] Falzone L., Scandurra G., Lombardo V., Gattuso G., Lavoro A., Distefano A.B., Scibilia G., Scollo P. (2021). A multidisciplinary approach remains the best strategy to improve and strengthen the management of ovarian cancer (Review). Int. J. Oncol..

[274] Ramirez R.A., Cass A.S., Das S., Low S.W., Mehrad M., Rickman O.B., Scherer P.M., Thomas K.E., Gillaspie E.A. (2022). A multidisciplinary approach to the work up and management of pulmonary carcinoid tumors and DIPNECH: A narrative review. Transl. Lung Cancer Res..

[275] Kapoor A., Prabhash K. (2022). Analysis of Outcomes With Addition of Immunotherapy to Chemoradiation Therapy for Non-Small Cell Lung Cancer. JAMA Oncol..

[276] Ramirez R.A., Lu J., Thomas K.E.H. (2018). Quality of life for non-small cell lung cancer patients in the age of immunotherapy. Transl. Lung Cancer Res..

[277] Smith K.E.R., Mansfield A.S. (2022). Validating chemoimmunotherapy in small-cell lung cancer. Lancet Oncol.

[278] Pennell N.A. (2022). Strategies and End Points in the Development of Novel Immunotherapy Trials for Patients With Unresectable, Locally Advanced Non-Small-Cell Lung Cancer. J. Clin. Oncol..

