# Supplementary material for: Clinical Relevance of Targeted Therapy and Immune-Checkpoint Inhibition in Lung Cancer
Source: Pharmaceutics. 2023 Apr 16;15(4):1252. doi: 10.3390/pharmaceutics15041252 (PMC10142433; doi:10.3390/pharmaceutics15041252)
Supplement: Supplementary file 1 [file pharmaceutics-15-01252-s001.zip › Supplementary Table S1.pdf]

Supplementary Table S1. Ongoing clinical studies on targeted therapies registered at clinicaltrials.gov

Target	Study ID	Status	Conditions	Interventions	Phase	Enrollment	Start Date
ALK	NCT01562015	C	NSCLC	Ganetespib	II	12	2012
ALK	NCT01712217	C	NSCLC	Onalespib; Crizotinib	I; II	220	2012
ALK	NCT01752400	C	NSCLC	Luminespib	II	6	2013
ALK	NCT01772797	C	NSCLC (ALK Mut)	Ceritinib; Luminespib	I	22	2013
ALK	NCT02040870	C	NSCLC	Ceritinib	I; II	103	2014
ALK	NCT02292550	C	NSCLC	Ribociclib; Ceritinib	I	27	2015
ALK	NCT02299505	C	NSCLC	Ceritinib	I	306	2015
ALK	NCT02336451	C	NSCLC (ALK Mut)	Ceritinib	II	156	2015
ALK	NCT02521051	O	NSCLC	Alectinib; Bevacizumab	I; II	43	2015
ALK	NCT02767804	O	NSCLC	Ensartinib; Crizotinib	III	290	2016
ALK	NCT03130881	O	NSCLC	PLB1003	I	60	2016
ALK	NCT03155009	C	Solid cancer; NSCLC	Alectinib	II	44	2017
ALK	NCT03202940	O	NSCLC	Alectinib; Cobimetinib	I; II	31	2017
ALK	NCT03410108	O	A NSCLC (ALK Mut)	Brigatinib	II	104	2018
ALK	NCT03456076	O	Solid cancer; NSCLC	Alectnib; Cisplatin; Vinorelbine; Gemcitabine; Pemetrexed; Carboplatin	III	255	2018
ALK	NCT03707938	O	A NSCLC; R NSCLC (ALK Mut)	Brigatinib	Early-I	35	2018
ALK	NCT03909971	O	Solid cancer; NSCLC	Lorlatinib	II	100	2019

ALK	NCT04009317	O	NSCLC (ALK Mut)	TQ-B3139; Crizotinib	III	260	2019
ALK	NCT04056572	O	NSCLC (ALK Mut)	TQ-B3139	II	135	2019
ALK	NCT04415320	O	NSCLC; M SSCLC (BM)	Ensartinib	II	37	2019
ALK	NCT04632758	O	NSCLC	WX-0593; Crizotinib	III	330	2019
ALK	NCT04111705	O	M NSCLC	Lorlatinib	II	112	2020
ALK	NCT04211922	O	NSCLC (ALK Mut)	Alkotinib	II	104	2020
ALK	NCT04227028	O	A NSCLC; M NSCLC; R NSCLC	Bevacizumab; Brigatinib	I	31	2020
ALK	NCT04362072	O	Cancer; NSCLC	Lorlatinib	IV	85	2020
ALK	NCT04634110	O	LC; M LC (BM)	Brigatinib	II	19	2020
ALK	NCT04837716	O	M NSCLC; R NSCLC; A NSCLC	Bevacizumab; Carboplatin; Ensartinib; Pemetrexed	I	12	2021
ALK; BRAF	NCT02314481	O	NSCLC	Atezolizumab; Vemurafenib; Alectinib; Trastuzumab	II	119	2017
ALK; BRAF; EGFR; RET; ROS1	NCT01922583	C	NSCLC	Luminespib	II	31	2014
ALK; BRAF; EGFR; ROS1	NCT03790228	O	Non-SqCC	Anlotinib; Pemetrexed; Carboplatin	I	43	2019

ALK; BRAF; KRAS; MET; PI3K; ROS1	NCT02276027	C	ADC; SqCC	Alpelisib; Capmatinib; Ceritinib; Binimetinib	II	66	2015
ALK; BRAF; NTRK; ROS1; RET	NCT04302025	O	NSCLC	Alectinib; Entrectinib; Vemurafenib; Cobimetinib; Chemotherapy; Pralsetinib	II	60	2020
ALK; EGFR	NCT02013219	C	NSCLC	Alectinib; Atezolizumab; Erlotinib	I	52	2014
ALK; MET	NCT04139317	O; NR	NSCLC	Capmatinib; Pembrolizumab	II	76	2020
ALK; RET	NCT03194893	O; NR	Cancer; LC	Alectinib; Crizotinib	III	200	2017
ALK; ROS1	NCT02927340	O	NSCLC	Lorlatinib	II	30	2016
ALK; ROS1	NCT03087448	O	NSCLC	Ceritinib; Trametinib	I; II	69	2017
ALK; ROS1	NCT03088930	C	LC; NSCLC	Crizotinib	II	3	2017
ALK; ROS1	NCT03607188	O	NSCLC	Alkotinib	I	18	2018
ALK; ROS1	NCT03672643	O	NSCLC (ALK Mut; ROS1 Mut)	Crizotinib	IV	75	2019
ALK; ROS1	NCT04641754	O	NSCLC	WX-0593	II	176	2019
ALK; ROS1	NCT04005144	O	A NSCLC (ALK Mut; ROS1 Mut)	Binimetinib; Brigatinib	I	18	2020
ALK; ROS1	NCT04292119	O	LC (ALK Mut;ROS1 Mut; MET Mut)	Lorlatinib; Crizotinib; Binimetinib; TNO155	I; II	96	2020
ALK; ROS1	NCT04541706	O; NR	A NSCLC	Lorlatinib	IV	111	2020

BRAF	NCT03543306	O	Cancer; M LC (BRAF V600E)	Dabrafenib; Trametinib	II	27	2018
BRAF	NCT04452877	O	Solid cancer; NSCLC	Dabrafenib; Trametinib	II	20	2020
BRAF	NCT04526782	O	NSCLC (BRAF V600E)	Encorafenib; Binimetinib; Docetaxel	II	144	2021
BRAF; RET; ROS1	NCT03178552	O	NSCLC	Alectinib; Atezolizumab; Pemetrexed; Cisplatin; Carboplatin; Gemcitabine; Entrectinib; Cobimetinib; Vemurafenib; Bevacizumab	II; III	700	2017
BRAF; ROS1	NCT04581824	O	LC; NSCLC	Dostarlimab; Pembrolizumab; Chemotherapy	II	240	2020
EGFR	NCT00034346	C	NSCLC; M NSCLC	Panitumumab; Paclitaxel; Carboplatin	II	194	2002
EGFR	NCT00101920	C	NSCLC; M NSCLC	Panitumumab	II	50	2003
EGFR	NCT00585533	C	LC	Erlotinib	II	40	2004
EGFR	NCT00266877	C	Solid cancer; NSCLC	Neratinib	II	172	2005
EGFR	NCT00411047	C	LC	Gefitinib	II	34	2005
EGFR	NCT02774278	C	Non-SqCC	Erlotinib	II	264	2005
EGFR	NCT00364780	C	NSCLC	Tesevatinib	II	55	2006
EGFR	NCT00370383	C	LC	Erlotinib; Satraplatin	II	100	2006
EGFR	NCT00385996	C	Solid cancer; NSCLC	Erlotinib	II	22	2006

EGFR	NCT00615758	C	NSCLC	Erlotinib	II	50	2006
EGFR	NCT00459342	C	NSCLC; R NSCLC; A NSCLC	Dasatinib	II	35	2007
EGFR	NCT00522145	C	Solid cancer; NSCLC	Tesevatinib	II	41	2007
EGFR	NCT00567359	C	NSCLC	Erlotinib	II	100	2007
EGFR	NCT00577707	C	NSCLC; LC	Erlotinib; Pemetrexed; Cisplatin	II	9	2007
EGFR	NCT00596648	C	Solid cancer; NSCLC	Cabozantinib; Erlotinib	I; II	92	2007
EGFR	NCT00608868	C	NSCLC	Gefitinib	IV	156	2007
EGFR	NCT00750698	C	NSCLC	Entinostat; Erlotinib	II	70	2008
EGFR	NCT00796549	C	Solid cancer; NSCLC	Afatinib	II	70	2008
EGFR	NCT01167244	C	NSCLC	BMS-690514	II	11	2010
EGFR	NCT01211483	C	A NSCLC	Patritumab; Erlotinib	I; II	222	2010
EGFR	NCT01221077	C	NSCLC	Linsitinib; Erlotinib	II	88	2011
EGFR	NCT01260181	C	Non-SqCC	Erlotinib	II	30	2011
EGFR	NCT01287754	C	NSCLC	Erlotinib	IV	24	2011
EGFR	NCT01404260	C	NSCLC	Gefitinib; Gemcitabine; Carboplatin	III	219	2011
EGFR	NCT01465802	C	NSCLC	Dacomitinib; Doxycycline; Alclometasone	II	236	2011
EGFR	NCT01513174	C	NSCLC	Gefitinib; Alaparib	I; II	186	2011

EGFR	NCT01516983	C	NSCLC; M NSCLC (BM)	Icotinib; Radiation	I; II	15	2011
EGFR	NCT01372384	C	Non-SqCC	Erlotinib	II	6	2012
EGFR	NCT01415011	C	Solid cancer; NSCLC	Afatinib	II	39	2012
EGFR	NCT01470716	O	NSCLC; A NSCLC	Erlotinib	II	26	2012
EGFR	NCT01530334	C	LC	Gefitinib	II	61	2012
EGFR	NCT01553942	O	LC	Afatinib; Radiation; Cisplatin; Pemetrexed	II	30	2012
EGFR	NCT01667562	C	NSCLC	Erlotinib	III	375	2012
EGFR	NCT01746251	O	NSCLC	Afatinib	II	92	2013
EGFR	NCT01953913	C	Solid cancer; NSCLC	Afatinib	III	542	2013
EGFR	NCT01967095	C	EGFR-Mutant; LC	Erlotinib	I	53	2013
EGFR	NCT02000531	C	Solid cancer; NSCLC	Erlotinib; Chemotherapy	IV	45	2014
EGFR	NCT02025114	C	NSCLC	Selumetinib	I; II	34	2014
EGFR	NCT02113813	C	NSCLC (EGFR Mut)	Naquotinib;Midazolam	I	133	2014
EGFR	NCT02155465	C	LC	Ruxolitinib; Erlotinib	I; II	22	2014
EGFR	NCT02191891	C	Solid cancer; NSCLC	Xentuzumab; Afatinib	I	32	2014
EGFR	NCT02264210	O	LC; SqCC; ADC; ADSC; LCC; NSCLC	Icotinib	II	128	2015
EGFR	NCT02442349	O	NSCLC	Osimertinib	II	171	2015
EGFR	NCT02444819	C	NSCLC	Olmutinib	II	33	2015
EGFR	NCT02448797	O	NSCLC	Icotinib; Chemotherapy	III	320	2015

EGFR	NCT02514174	C	Solid cancer; NSCLC	Afatinib	IV	25	2015
EGFR	NCT02714010	O	NSCLC	EGFR-TKI; Radiation	III	601	2015
EGFR	NCT02804776	C	NSCLC	Gefitinib	II	15	2015
EGFR	NCT02183883	O	NSCLC	Afatinib	II	48	2016
EGFR	NCT02496663	O	M NSCLC; R NSCLC; A NSCLC	Necitumumab; Osimertinib	I	100	2016
EGFR	NCT02520778	O	A Non-SqCC; M Non-SqCC; A NSCLC; M NSCLC	Navitoclax; Osimertinib	I	50	2016
EGFR	NCT02716311	O	NSCLC	Afatinib; Cetuximab	II	118	2016
EGFR	NCT02726568	O	NSCLC; M NSCLC (BM)	Icotinib; Radiation	II	30	2016
EGFR	NCT02736513	O	LC	Osimertinib	II	40	2016
EGFR	NCT02789345	O	NSCLC	Ramucirumab; Necitumumab; Osimertinib	I	74	2016
EGFR	NCT02820116	O	NSCLC	Icotinib	II	67	2016
EGFR	NCT02841579	C	NSCLC	Osimertinib	II	22	2016
EGFR	NCT02914990	C	NSCLC	BPI-15086	I	36	2016
EGFR	NCT02954523	O	NSCLC (EGFR Mut)	Dasatinib; Osimertinib	I; II	10	2016
EGFR	NCT02976116	C	NSCLC	Fruquintinib; Gefitinib	II	50	2016
EGFR	NCT03053219	C	Solid cancer; NSCLC	AC0010	I	7	2016
EGFR	NCT02856893	O	NSCLC	Osimertinib; Gefitinib	II	156	2017

EGFR	NCT03054038	O	NSCLC	Afatinib; Necitumumab	I	60	2017
EGFR	NCT03066206	O	R NSCLC; A NSCLC (EGFR Exon 20 Mut; ERBB2 Mut)	Poziotinib	II	80	2017
EGFR	NCT03122717	O	NSCLC	Gefitinib; Osimertinib	I; II	48	2017
EGFR	NCT03292133	O	LC	Nazartinib; Gefitinib	II	11	2017
EGFR	NCT03318939	O	NSCLC	Poziotinib	II	603	2017
EGFR	NCT03386955	O	NSCLC	BPI-7711	I; II	215	2017
EGFR	NCT03502850	O	A NSCLC; M NSCLC	ASK120067	I; II	507	2017
EGFR	NCT03239340	O	A NSCLC; M NSCLC (EGFR Mut)	Osimertinib	II	154	2018
EGFR	NCT03333343	O	NSCLC (EGFR Mut)	Nazartinib; Trametinib; Ribociclib; Naporafenib; Capmatinib; Gefitinib	I	105	2018
EGFR	NCT03349203	O	NSCLC (EGFR Mut)	Icotinib	II	60	2018
EGFR	NCT03381066	O	Resected NSCLC (EGFR Mut)	Gefitinib; Pemetrexed; Cisplatin; Vinorelbine	III	225	2018
EGFR	NCT03381274	O	Solid cancer; NSCLC	Oleclumab; Osimertinib; AZD4635	I; II	43	2018
EGFR	NCT03381430	O; NR	LC	Gefitinib	NA	50	2018
EGFR	NCT03389256	O; NR	Thoracic cancer; NSCLC	Apatinib; EGFR-TKI	II	144	2018

EGFR	NCT03392246	O	NSCLC	Osimertinib; Selumetinib	II	25	2018
EGFR	NCT03396185	O	A NSCLC (EGFR Mut)	Icotinib	II	30	2018
EGFR	NCT03410043	O	NSCLC; R NSCLC; A NSCLC	Osimertinib; Radiation	II	143	2018
EGFR	NCT03414814	O	A NSCLC; M NSCLC	Osimertinib	II	28	2018
EGFR	NCT03433469	O	E NSCLC	Osimertinib	II	27	2018
EGFR	NCT03434418	O	NSCLC	Osimertinib	II	37	2018
EGFR	NCT03446417	O	Solid cancer; NSCLC	ZN-e4	I; II	140	2018
EGFR	NCT03455829	O	Solid cancer; NSCLC; LC	Lerociclib; Osimertinib	I; II	30	2018
EGFR	NCT03457337	O	A NSCLC (EGFR Mut)	Tegafur; Gefitinib; Gefitinib	II	200	2018
EGFR	NCT03516214	O	Bronchial cancer	Nazartinib; Trametinib	I	24	2018
EGFR	NCT03521154	O	NSCLC	Osimertinib	III	200	2018
EGFR	NCT03535363	O	NSCLC	Osimertinib	I	6	2018
EGFR	NCT03567642	O	LC	Osimertinib; Platinum; Etoposide	I	20	2018
EGFR	NCT03586453	O	Solid cancer; NSCLC	Osimertinib	II	30	2018
EGFR	NCT03599518	C	NSCLC	DS-1205c; Gefitinib	I	21	2018
EGFR	NCT03623750	O	Solid cancer; NSCLC	EGFR-TKI; EGF-PTI; Cyclophosphamide	I; II	30	2018

EGFR	NCT03667820	O	NSCLC	Osimertinib; Radiation	II	42	2018
EGFR	NCT03727724	O	Solid cancer; NSCLC	Afatinib; Cetuximab	II	37	2018
EGFR	NCT03749213	O	NSCLC (EGFR Mut)	Icotinib	II	36	2018
EGFR	NCT03755102	O	LC; M LC (EGFR Mut)	Dacomitinib; Osimertinib	Early-I	24	2018
EGFR	NCT03804580	O	LC	Osimertinib	II	100	2018
EGFR	NCT04058704	O	NSCLC; BM Metastases	Icotinib; Radiation	III	296	2018
EGFR	NCT03497767	O	M NSCLC	Osimertinib; Radiation	II	80	2019
EGFR	NCT03727867	O; NR	A NSCLC	EGFR-TKI; Radiation	III	300	2019
EGFR	NCT03769103	O	A NSCLC; M NSCLC (BM)	Osimertinib; Radiation	II	76	2019
EGFR	NCT03787992	O	A NSCLC; M NSCLC (EGFR Mut)	Alflutinib; Gefitinib	III	358	2019
EGFR	NCT03807778	O	NSCLC	Mobocertinib	I	33	2019
EGFR	NCT03812809	O	NSCLC	BPI-7711	II	226	2019
EGFR	NCT03831932	O	A NSCLC; M NSCLC	Osimertinib; Telaglenastat hydrochloride	I	18	2019
EGFR	NCT03849768	O	NSCLC	Aumolertinib; Gefitinib	III	350	2019
EGFR	NCT03853551	C	NSCLC	Osimertinib	IV	60	2019
EGFR	NCT03856697	O; NR	A NSCLC	Abivertinib; Gefitinib	III	406	2019
EGFR	NCT03904823	O; NR	NSCLC (EGFR Mut)	Famitinib;Aumolertinib	II	58	2019

EGFR	NCT03909334	O	A NSCLC; M NSCLC (EGFR Mut)	Osimertinib; Ramucirumab	II	150	2019
EGFR	NCT03974022	O	NSCLC	DZD9008	I; II	220	2019
EGFR	NCT03983811	O; NR	Solid cancer; NSCLC	Icotinib; Chemotherapy	NA	174	2019
EGFR	NCT04001777	O	NSCLC (EGFR Mut)	Palcitoclax; Osimertinib	I	60	2019
EGFR	NCT04028778	O	LC; NSCLC	Gefitinib; Anlotinib	III	310	2019
EGFR	NCT04035486	O	NSCLC	Osimertinib; Pemetrexed; Carboplatin; Cisplatin	III	586	2019
EGFR	NCT04036682	O	NSCLC (EGFR Exon 20 Mut)	CLN-081	I; II	80	2019
EGFR	NCT04063462	O; NR	NSCLC	Pyrotinib	II	60	2019
EGFR	NCT04075396	O	Solid cancer; NSCLC	Lazertinib	I; II	28	2019
EGFR	NCT04143607	O	A NSCLC; M NSCLC	ASK120067; Gefitinib	III	334	2019
EGFR	NCT04206072	O	NSCLC (EGFR Mut)	D-0316; Icotinib	II; III	362	2019
EGFR	NCT03758677	O; NR	NSCLC	Apatinib	II	30	2020
EGFR	NCT04129502	O	A NSCLC; M NSCLC	Mobocertinib; Pemetrexed; Cisplatin; Carboplatin	III	318	2020
EGFR	NCT04181060	O	A Non-SqCC; M Non-SqCC; R Non-SqCC; A LC	Bevacizumab; Osimertinib	III	300	2020
EGFR	NCT04204473	O	NSCLC	TY-9591	I	126	2020

EGFR	NCT04233021	O	M NSCLC; M NSCLC (BM); NSCLC (EGFR Mut)	Osimertinib	II	113	2020
EGFR	NCT04239833	O; NR	NSCLC	SH-1028; Gefitinib	III	240	2020
EGFR	NCT04248829	O	NSCLC	Lazertinib; Gefitinib	III	380	2020
EGFR	NCT04401059	O	Solid cancer; NSCLC; ADC	Elemene; EGFR-TKI	IV	468	2020
EGFR	NCT04402008	O	NSCLC	Poziotinib	I; II	76	2020
EGFR	NCT04410796	O	M NSCLC	Osimertinib; Carboplatin; Pemetrexed	II	571	2020
EGFR	NCT04413201	O	Non-SqCC	Afatinib; Osimertinib	IV	126	2020
EGFR	NCT04438902	O; NR	NSCLC	Osimertinib; Anlotinib	II	30	2020
EGFR	NCT04448379	O	A NSCLC; M NSCLC	JMT101	I	48	2020
EGFR	NCT04455594	O; NR	A NSCLC	Almonertinib; Erlotinib; Cisplatin; Carboplatin; Pemetrexed	II	168	2020
EGFR	NCT04470076	O; NR	NSCLC (EGFR Mut)	Afatinib; Pemetrexed; Gemcitabine; Cisplatin; Carboplatin	II	30	2020
EGFR	NCT04479306	O	R NSCLC; A NSCLC	Alisertib; Osimertinib; Sapanisertib	I	40	2020

EGFR	NCT04487080	O	Solid cancer; NSCLC	Amivantamab; Osimertinib; Lazertinib	III	1000	2020
EGFR	NCT04500704	O; NR	NSCLC	Almonertinib; Almonertinib; Carboplatin; Pemetrexed	III	166	2020
EGFR	NCT04500717	O; NR	NSCLC	Almonertinib; Almonertinib; Carboplatin; Pemetrexed	III	460	2020
EGFR	NCT04504071	O	M NSCLC (EGFR Mut)	Dacomitinib	II	30	2020
EGFR	NCT04511533	O	M NSCLC	Dacomitinib	IV	100	2020
EGFR	NCT04512430	O	NSCLC (EGFR Mut)	Atezolizumab; Bevacizumab; Carboplatin; Pemetrexed	II	26	2020
EGFR	NCT04545710	O	LC	Abemaciclib; Osimertinib	II	18	2020
EGFR	NCT04552613	O	NSCLC (EGFR Mut)	EGFR-TKI; Pemetrexed; Carboplatin	NA	110	2020
EGFR	NCT04553887	O; NR	NSCLC	Almonertinib	II	53	2020
EGFR	NCT04563871	O	NSCLC	Osimertinib	II	80	2020
EGFR	NCT04592666	O; NR	LC; NSCLC (EGFR Mut)	Almonertinib;Pemetrexed;Carboplatin	II	226	2020
EGFR	NCT04622072	O	NSCLC	XZP-5809-TT1	I	90	2020
EGFR	NCT04643847	O; NR	M NSCLC (BM; EGFR Mut)	Almonertinib	II	47	2020

EGFR	NCT04646824	O	M NSCLC	Almonertinib Pemetrexed; Cisplatin	II	10	2020
EGFR	NCT04811001	O	NSCLC	Osimertinib; Dacomitinib	II	170	2020
EGFR	NCT04838548	O	Solid cancer; NSCLC	MRG003	II	90	2020
EGFR	NCT04858958	O	NSCLC	Furmonertinib	I	30	2020
EGFR	NCT03866499	O	NSCLC	BPI-7711; Gefitinib	III	294	2021
EGFR	NCT04285671	O	M NSCLC; R NSCLC; A NSCLC	Necitumumab; Osimertinib; Trastuzumab	I; II	26	2021
EGFR	NCT04486833	O; NR	Solid cancer; NSCLC	Quaratusugene ozeplasmid; Osimertinib	I; II	100	2021
EGFR	NCT04671303	O; NR	LC	Anlotinib; Allitinib	II	36	2021
EGFR	NCT04676477	O	NSCLC	Patritumab; Deruxtecan; Osimertinib	I	252	2021
EGFR	NCT04685070	O; NR	A ADC	Almonertinib	II	56	2021
EGFR	NCT04687241	O; NR	NSCLC	Almonertinib	III	192	2021
EGFR	NCT04695925	O; NR	NSCLC (EGFR Mut)	Osimertinib; Pemetrexed; Carboplatin	III	291	2021
EGFR	NCT04755738	O; NR	A NSCLC	Almonertinib	II	234	2021
EGFR	NCT04762199	O	A NSCLC; M NSCLC	MRX-2843; Osimertinib	I	69	2021
EGFR	NCT04762459	O; NR	NSCLC	Almonertinib; Pemetrexed; Cisplatin	III	606	2021

EGFR	NCT04778800	O; NR	NSCLC; M NSCLC (BM)	Almonertinib; Chemotherapy	NA	60	2021
EGFR	NCT04780568	O; NR	M NSCLC; A NSCLC	Osimertinib; Tegavivint	I	18	2021
EGFR	NCT04797806	O	NSCLC	Anlotinib; Icotinib	III	206	2021
EGFR	NCT04808752	O; NR	LC	Almonertinib	II	60	2021
EGFR	NCT04833205	O	M LC (BM)	Nimotuzumab	II	30	2021
EGFR	NCT04870190	O; NR	NSCLC	Almonertinib; Osimertinib	III	232	2021
EGFR	NCT04882345	O; NR	LC (EGFR Mut)	Almonertinib	II	40	2021
EGFR	NCT04922138	O; NR	LC	Aumolertinib	II	52	2021
EGFR	NCT04923906	O; NR	NSCLC	Aumolertinib	III	624	2021
EGFR; KRAS	NCT01859026	O; NR	LC; NSCLC	Binimetinib; Erlotinib	I	43	2013
EGFR; MET	NCT00854308	C	NSCLC	Erlotinib; Onartuzumab	II	137	2009
EGFR; MET	NCT01887886	C	Non-SqCC	Erlotinib; Onartuzumab;	III	10	2013
EGFR; MET	NCT01900652	C	Cancer; NSCLC	Emibetuzumab; Erlotinib	II	111	2013
EGFR; MET	NCT01911507	O; NR	R NSCLC	Capmatinib; Erlotinib	I	35	2013
EGFR; MET	NCT01982955	O; NR	NSCLC	Tepotinib; Gefitinib; Pemetrexed; Cisplatin; Carboplatin	I; II	88	2013
EGFR; MET	NCT01897480	O	Solid cancer; NSCLC	Emibetuzumab; Erlotinib	II	150	2013
EGFR; MET	NCT02031744	C	NSCLC	Erlotinib; Onartuzumab	III	530	2014
EGFR; MET	NCT02335944	C	NSCLC	Capmatinib; Nazartinib	I; II	177	2015

EGFR; MET	NCT02609776	O	NSCLC	Amivantamab; Lazertinib; Carboplatin; Pemetrexed	I	460	2016
EGFR; MET	NCT03758287	O	NSCLC	CT053PTSA; Gefitinib	I; II	158	2016
EGFR; MET	NCT03778229	O	Cancer; NSCLC	Osimertinib; Savolitinib	II	259	2019
EGFR; MET	NCT03940703	O	NSCLC	Tepotinib; Osimertinib	II	120	2019
EGFR; MET	NCT04338243	O	NSCLC (EGFR T790M-negative; MET Mut)	Glumetinib	I; II	70	2019
EGFR; MET	NCT04606771	O	NSCLC	Osimertinib; Savolitinib	II	56	2020
EGFR; MET	NCT04743505	O; NR	M NSCLC	APL-101; Osimertinib	I; II	22	2021
EGFR; MET	NCT04816214	O; NR	Cancer; NSCLC	Capmatinib; Pemetrexed; Cisplatin; Carboplatin; Osimertinib	III	245	2021
EGFR; MET; PI3K	NCT01121575	C	NSCLC	Crizotinib; Dacomitinib	I	70	2010
EGFR; PI3K	NCT01487265	C	NSCLC	Buparlisib; Erlotinib	II	37	2014
KRAS	NCT01395758	C	M NSCLC	Tivantinib; Erlotinib; Pemetrexed; Docetaxel; Gemcitabine	II	96	2011
KRAS	NCT01427946	C	NSCLC	Retaspimycin hydrochloride; Everolimus	I; II	47	2011
KRAS	NCT02047344	C	A NSCLC	Antroquinonol	II	31	2013

KRAS	NCT02283320	C	NSCLC; SqCC (KRAS Mut)	BIND-014 (Docetaxel nanoparticles)	II	69	2014
KRAS	NCT02642042	O	R NSCLC; A NSCLC (KRAS Mut)	Docetaxel; Trametinib	II	60	2016
KRAS	NCT02964689	O	A NSCLC (KRAS Mut)	Binimetinib; Pemetrexed; Cisplatin	I	18	2017
KRAS	NCT03170206	O	LC	Binimetinib; Palbociclib	I; II	72	2017
KRAS	NCT03095612	O	NSCLC	Selinexor	I; II	59	2018
KRAS	NCT03520842	O	R NSCLC; A NSCLC; M NSCLC (BM; KRAS Mut)	Methotrexate; Regorafenib	II	18	2018
KRAS	NCT03681483	O	A NSCLC	RO5126766	I	15	2018
KRAS	NCT03704688	O	NSCLC (KRAS Mut)	Trametinib; Ponatinib	I; II	12	2018
KRAS	NCT03808558	O	NSCLC (KRAS Mut)	TVB-2640	II	12	2019
KRAS	NCT04303780	O	A NSCLC; M NSCLC (KRAS G12C)	AMG 510; Docetaxel	III	345	2020
KRAS	NCT04620330	O	NSCLC (KRAS Mut)	VS-6766; Defactinib	II	100	2020
KRAS	NCT03990077	O NR	NSCLC	HL-085; Docetaxel	I	27	2021
KRAS	NCT04625647	O NR	ADC; NSCLC; R Non-SqCC; A Non-SqCC	Sotorasib	II	116	2021
KRAS	NCT04685135	O	M NSCLC; A NSCLC	Adagrasib; Docetaxel	III	452	2021

KRAS	NCT04735068	O; NR	NSCLC (KRAS Mut)	Binimetinib; Hydroxychloroquine	II	29	2021
KRAS	NCT04933695	O; NR	NSCLC	Sotorasib	II	170	2021
KRAS; RET	NCT01829217	C	LC	Sunitinib	II	13	2013
MET	NCT01519804	C	Non-SqCC	Cisplatin; Carboplatin; Onartuzumab; Paclitaxel	II	108	2012
MET	NCT02435121	C	Cancer	SAR125844	II	1	2015
MET	NCT02544633	C	NSCLC	MGCD265	II	68	2015
MET	NCT02750215	C	A NSCLC	Capmatinib	II	20	2016
MET	NCT02897479	O	SCC	Savolitinib	II	76	2016
MET	NCT02929290	C	NSCLC	BPI-9016M	I	77	2017
MET	NCT03539536	O	NSCLC	Telisotuzumab vedotin	II	233	2018
MET	NCT03574753	O	R SqCC; A SqCC	Telisotuzumab vedotin	II	28	2018
MET	NCT03693339	O	Cancer; M LC (MET Mut)	Capmatinib	II	27	2018
MET	NCT03737994	O	A Non-SqCC	Alectinib; Brigatinib; Carboplatin; Ceritinib; Cisplatin; Crizotinib; Ensartinib; Lorlatinib; Pemetrexed	II	660	2019
MET	NCT04077099	O	NSCLC	REGN5093	I; II	111	2020
MET	NCT04258033	O	NSCLC	Bozitinib	II	185	2020

MET	NCT04398940	O	NSCLC	TQ-B3139	II	71	2020
MET	NCT04427072	O	Solid cancer; NSCLC	Capmatinib;Docetaxel	III	90	2020
MET	NCT04677595	O	NSCLC	Capmatinib	II	35	2021
MET	NCT04739358	O; NR	A NSCLC (MET Mut)	Tepotinib	I; II	65	2021
MET	NCT04768296	O	SCLC	Berzosertib;Topotecan	II	86	2021
MET	NCT04923945	O; NR	M NSCLC	Savolitinib	III	163	2021
MET	NCT04926831	O; NR	NSCLC	Capmatinib	II	38	2021
MET; NTRK; ROS1	NCT01639508	O	NSCLC	Cabozantinib	II	68	2012
MET; ROS1	NCT04084717	O	Non-SqCC; A NSCLC (ROS1 Mut; MET Mut)	Crizotinib	II	50	2019
PI3K	NCT01493843	C	NSCLC	Pictilisib; Bevacizumab; Carboplatin; Paclitaxel	II	501	2012
RET	NCT01823068	C	NSCLC	Vandetanib	II	17	2014
RET	NCT04268550	O	R NSCLC; A NSCLC	Selpercatinib	II	124	2020
RET	NCT04819100	O; NR	Solid cancer; NSCLC	Selpercatinib	III	170	2021
ROS1	NCT01945021	C	NSCLC (ROS1 Mut)	Crizotinib	II	129	2013
ROS1	NCT03608007	O	LC (ROS1 Mut)	Ensartinib	II	69	2018
ROS1	NCT03612154	O	NSCLC	Lorlatinib	II	35	2019
ROS1	NCT03972189	O	NSCLC (ROS1 Mut)	TQ-B3101	II	111	2019

ROS1	NCT04395677	O	NSCLC	Taletrectinib	II	106	2020
ROS1	NCT04603807	O; NR	Solid cancer; NSCLC	Entrectinib; Crizotinib	III	220	2021
ROS1	NCT04621188	O	M NSCLC	Lorlatinib	II	84	2021
ROS1	NCT04919811	O; NR	NSCLC	Taletrectinib	II	119	2021

Abbreviations: **C**, Closed; **O**, Open; **NR**, Not Recruiting; **NA**, Not Associated; **A**, Advanced; **M**, Metastatic; **R**, Recurrent; **SCLC**, Small Cell Lung Cancer; **LC**, Lung Cancer; **ADC**, Adenocarcinoma; **NSCLC**, Non Small Cell Lung Cancer; **SqCC**, Squamous Carcinoma; **SCC**, Sarcomatous Carcinoma.
