# Supplementary material for: Clinical Relevance of Targeted Therapy and Immune-Checkpoint Inhibition in Lung Cancer
Source: Pharmaceutics. 2023 Apr 16;15(4):1252. doi: 10.3390/pharmaceutics15041252 (PMC10142433; doi:10.3390/pharmaceutics15041252)
Supplement: Supplementary file 1 [file pharmaceutics-15-01252-s001.zip › Supplementary Table S2.pdf]

Supplementary Table S2. Ongoing clinical studies on immune-checkpoint inhibitors registered at clinicaltrials.gov

**Target**	**Study ID**	**Status**	**Conditions**	**Interventions**	**Phase**	**Enrollment**	**Start Date**
CTLA-4	NCT02221739	C	NSCLC	Ipilimumab; Radiation	I; II	39	2014
CTLA-4	NCT04544644	O; NR	NSCLC	AK104; Anlotinib	II	30	2020
CTLA-4; PD-1	NCT02785952	O; NR	R SqCC; A SqCC	Ipilimumab; Nivolumab	III	350	2015
CTLA-4; PD-1	NCT02696993	O	M NSCLC (BM); A NSCLC	Ipilimumab; Nivolumab; Radiation	I; II	88	2016
CTLA-4; PD-1	NCT03262779	O	Cancer; NSCLC	Ipilimumab; Nivolumab	II	50	2017
CTLA-4; PD-1	NCT03391869	O	ADC; NSCLC; A LC	Ipilimumab; Nivolumab; Radiation	III	360	2017
CTLA-4; PD-1	NCT03377023	O	NSCLC; LC; M NSCLC	Nivolumab; Ipilimumab; Nintedanib	I; II	98	2018
CTLA-4; PD-1	NCT03430063	O; NR	A NSCLC	Cemiplimab; Ipilimumab	II	28	2018
CTLA-4; PD-1	NCT03468985	O; NR	M Non-SqCC; R Non-SqCC; A NSCLC	Cabozantinib; Ipilimumab; Nivolumab	II	169	2018
CTLA-4; PD-1	NCT03515629	O; NR	NSCLC	Cemiplimab; Chemotherapy; Pembrolizumab	III	5	2018
CTLA-4; PD-1	NCT03516981	O	A NSCLC	Pembrolizumab; Favezelimab; Lenvatinib; Quavonlimab	II	318	2018
CTLA-4; PD-1	NCT03575793	O	LC; SCLC	Nivolumab; Plinabulin; Ipilimumab	I; II	35	2018

CTLA-4; PD-1	NCT03581487	O	R NSCLC; A NSCLC	Durvalumab; Selumetinib; Tremelimumab	I; II	40	2019
CTLA-4; PD-1	NCT04013542	O	A NSCLC (ALK Mut; EGFR Mut)	Ipilimumab; Nivolumab; Radiation	I	20	2019
CTLA-4; PD-1	NCT04043195	O	A NSCLC	Nivolumab; Oxaliplatin; Ipilimumab	I; II	30	2019
CTLA-4; PD-1	NCT04929041	O; NR	A NSCLC	Carboplatin; Ipilimumab; Paclitaxel; Nivolumab; Pembrolizumab; Pemetrexed; Radiation	II; III	100	2022
CTLA4; PD-L1	NCT03838848	O	A NSCLC	KN046	II	149	2019
CTLA-4; PD-L1	NCT02542293	O; NR	NSCLC	Durvalumab; Tremelimumab; Paclitaxel; Carboplatin; Gemcitabine; Cisplatin; Pemetrexed	III	953	2015
CTLA-4; PD-L1	NCT03164616	O	NSCLC	Durvalumab; Tremelimumab; Abraxane; Carboplatin; Gemcitabine; Cisplatin	III	1193	2017
CTLA-4; PD-L1	NCT03373760	O; NR	R SqCC; A SqCC	Durvalumab; Tremelimumab	II	132	2017
CTLA-4; PD-L1	NCT03275597	O	NSCLC; A NSCLC	Durvalumab; Tremelimumab; Radiation	I	31	2018
CTLA-4; PD-L1	NCT03963414	O	SCLC	Durvalumab; Tremelimumab; Carboplatin; Etoposide	I	18	2020

PD-1	NCT01928576	O	NSCLC	Azacitidine; Entinostat; Nivolumab	II	120	2013
PD-1	NCT02422381	O; NR	Cancer; NSCLC	Pembrolizumab; Gemcitabine	I; II	16	2015
PD-1	NCT02439450	O; NR	NSCLC	Viagenpumatucel-L; Nivolumab; Pembrolizumab; Pemetrexed	I; II	121	2015
PD-1	NCT02504372	O; NR	NSCLC	Pembrolizumab	III	1177	2015
PD-1	NCT02546986	O; NR	Cancer; NSCLC	Azaciditine; Pembrolizumab	II	100	2015
PD-1	NCT02621398	O; NR	NSCLC	Radiation; Carboplatin; Paclitaxel; Pembrolizumab	I	30	2016
PD-1	NCT02733159	O; NR	Cancer; NSCLC	Pembrolizumab	II	62	2016
PD-1	NCT02840994	C	NSCLC	BN-CV301; Pembrolizumab; Nivolumab	I	24	2016
PD-1	NCT02864394	O; NR	Cancer; NSCLC	Pembrolizumab; Docetaxel	III	425	2016
PD-1	NCT03003468	O; NR	NSCLC	Imprime PGG; Pembrolizumab	I; II	35	2016
PD-1	NCT03360630	O	LC; Cancer	Anti-PD-1; DC-CIK	I; II	60	2016
PD-1	NCT02818920	O; NR	NSCLC	Pembrolizumab	II	35	2017
PD-1	NCT02824965	O; NR	NSCLC	Pembrolizumab; Coxsackievirus A21	I	11	2017
PD-1	NCT02934503	C	SCLC	Pembrolizumab; Cisplatin; Carboplatin; Etoposide; Radiation	II	5	2017

PD-1	NCT02938624	O	E NSCLC	Pembrolizumab	I	28	2017
PD-1	NCT03048500	O; NR	R NSCLC; A NSCLC	Metformin; Nivolumab	II	17	2017
PD-1	NCT03083808	O; NR	NSCLC	Pembrolizumab	II	35	2017
PD-1	NCT03150875	O; NR	NSCLC	Sintilimab; Docetaxel	III	290	2017
PD-1	NCT03181308	C	Cancer; NSCLC	Carotuximab; Nivolumab	I	11	2017
PD-1	NCT03288870	O; NR	A NSCLC	BCD-100; Docetaxel	II; III	218	2017
PD-1	NCT03301688	C	NSCLC	Toripalimab	I	38	2017
PD-1	NCT03307759	O; NR	NSCLC	Pembrolizumab; Radiation	I	13	2017
PD-1	NCT03308942	O; NR	Cancer	Niraparib; Pembrolizumab; Dostarlimab	II	53	2017
PD-1	NCT03322540	C	LC	Pembrolizumab; Epacadostat	II	154	2017
PD-1	NCT03326752	C	A NSCLC	DV281; Anti-PD-1	I	26	2017
PD-1	NCT03358875	O; NR	NSCLC	Tislelizumab; Docetaxel	III	805	2017
PD-1	NCT03875092	O; NR	NSCLC	Pembrolizumab; Paclitaxel; Carboplatin	III	125	2017
PD-1	NCT03322566	C	LC	Pembrolizumab; Epacadostat; Chemotherapy	II	233	2018
PD-1	NCT03383302	O	E NSCLC	Radiation; Nivolumab	I; II	31	2018
PD-1	NCT03409614	O; NR	NSCLC	REGN2810; Chemotherapy	III	790	2018
PD-1	NCT03436056	O; NR	M NSCLC	Radiation; Pembrolizumab	I	24	2018
PD-1	NCT03513666	O; NR	NSCLC	Toripalimab; Pemetrexed; Carboplatin	II	40	2018

PD-1	NCT03526887	O	LC	Pembrolizumab	II	110	2018
PD-1	NCT03557411	O	NSCLC	Camrelizumab	II	42	2018
PD-1	NCT03562871	O; NR	NSCLC	IO102; Pembrolizumab; Carboplatin; Pemetrexed	I; II	108	2018
PD-1	NCT03631784	O; NR	NSCLC	Pembrolizumab; Paclitaxel; Carboplatin; Cisplatin; Pemetrexed; Radiation	II	216	2018
PD-1	NCT03656094	O	M NSCLC	Pembrolizumab; Chemotherapy	II	98	2018
PD-1	NCT03666728	T	LC	Camrelizumab; Bevacizumab	II	5	2018
PD-1	NCT03663205	O; NR	NSCLC	Tislelizumab; Cisplatin; Carboplatin;Pemetrexed	III	334	2018
PD-1	NCT03668496	O; NR	SqCC; A LC	Camrelizumab	III	390	2018
PD-1	NCT03693326	O	Cancer; M LC	PDR001	II	70	2018
PD-1	NCT03709706	O	Cancer	Letetresgene autoleucel; Pembrolizumab	I; II	54	2018
PD-1	NCT03866980	O	A NSCLC	AK105; Carboplatin; Pemetrexed	III	360	2018
PD-1	NCT03866993	O	A NSCLC; SqCC	AK105; Paclitaxel; Carboplatin	III	338	2018
PD-1	NCT04731909	O	ES-SCLC	Toripalimab; Anlotinib; Cisplatin; Carboplatin	NA	80	2018
PD-1	NCT03623776	O; NR	NSCLC	JS001; Pemetrexed; Carboplatin	II	80	2019

PD-1	NCT03774732	O	NSCLC; M NSCLC	Radiation; Pembrolizumab; Chemotherapy	III	460	2019
PD-1	NCT03802240	O	Non-SqCC	Sintilimab; Bevacizumab; Pemetrexed; Cisplatin	III	600	2019
PD-1	NCT03808480	O	NSCLC	Chemotherapy; Nivolumab	II	22	2019
PD-1	NCT03829319	O; NR	Non-SqCC	Pembrolizumab; Carboplatin; Cisplatin; Pemetrexed; Lenvatinib	III	726	2019
PD-1	NCT03829332	O; NR	NSCLC	Pembrolizumab; Lenvatinib	III	620	2019
PD-1	NCT03846310	O	NSCLC; Non-SqCC (EGFR Mut)	Etrumadenant; Zimberelimab; Carboplatin; Pemetrexed; Pembrolizumab	I	116	2019
PD-1	NCT03903887	O	NSCLC	Anti-PD1-activated TILs	I; II	20	2019
PD-1	NCT03912389	O	Non-SqCC	BCD-100; Pemetrexed; Cisplatin; Carboplatin	III	292	2019
PD-1	NCT03952403	O	Cancer; NSCLC	HLX10; Bevacizumab; Carboplatin; Pemetrexed	III	636	2019
PD-1	NCT03976323	O; NR	Cancer; Non-sqCC	Pembrolizumab; Pemetrexed; Carboplatin; Cisplatin; Olaparib	III	792	2019
PD-1	NCT03976362	O	Cancer; SqCC; NSCLC	Pembrolizumab; Carboplatin; Paclitaxel; Olaparib	III	735	2019
PD-1	NCT03991819	O	NSCLC	Binimetinib; Pembrolizumab	I	40	2019

PD-1	NCT03994744	O	SCLC; R SCLC; ES-SCLC	anti-PD-1; Metformin	II	68	2019
PD-1	NCT04033354	O	SqCC	HLX10; Carboplatin; Paclitaxel	III	516	2019
PD-1	NCT04047186	O; NR	E NSCLC	Nivolumab	II	50	2019
PD-1	NCT04055792	O	SCLC	Sintilimab; Anlotinib	II	52	2019
PD-1	NCT04059874	O	NSCLC	Anti-PD-1; Donafenib	I	20	2019
PD-1	NCT04063163	O	ES-SCLC	HLX10; Carboplatin; Etoposide	III	567	2019
PD-1	NCT04063449	O	Non-sqCC	Endostar; anti-PD-1	NA	170	2019
PD-1	NCT04108013	O; NR	LC; NSCLC	Camrelizumab; Carboplatin; Paclitaxel	II	38	2019
PD-1	NCT04124731	O; NR	Cancer; NSCLC	Sintilimab; Anlotinib; Chemotherapy	II	98	2019
PD-1	NCT04133337	O; NR	Thoracic cancer; NSCLC	Apatinib; Camrelizumab	I; II	20	2019
PD-1	NCT04144608	O	A NSCLC	Treprilumab; Chemotherapy	II	30	2019
PD-1	NCT04165070	O	Cancer; NSCLC	Pembrolizumab; Carboplatin; Paclitaxel; Pemetrexed; Vibostolimab; MK-5890	II	180	2019
PD-1	NCT04171284	O; NR	SqCC	SCT-I10; Docetaxel;	III	360	2019
PD-1	NCT04303130	O	NSCLC	Camrelizumab	II	52	2019
PD-1	NCT04676412	O	NSCLC	Pembrolizumab; Lenvatinib	III	620	2019

PD-1	NCT04716933	O	Non-SqCC	Pembrolizumab; Carboplatin; Cisplatin; Pemetrexed; Lenvatinib	III	200	2019
PD-1	NCT04165083	O	Cancer; NSCLC	Pembrolizumab; MK-4830	II	60	2020
PD-1	NCT04165096	O; NR	Cancer; NSCLC	Pembrolizumab; MK-5890; MK-4830; Diphenhydramine; Acetaminophen; MK-0482	II	135	2020
PD-1	NCT04173325	O	SCLC	Nivolumab; Irinotecan	I	10	2020
PD-1	NCT04189094	O; NR	SCLC	Sintilimab; Etoposide; Cisplatin; Radiation	II	140	2020
PD-1	NCT04205812	O	M SqCC; M Non-SqCC	Retifanlimab; Pemetrexed; Cisplatin; Carboplatin; Paclitaxel	III	530	2020
PD-1	NCT04252365	O; NR	NSCLC	Sintilimab; Pembrolizumab	II	20	2020
PD-1	NCT04331626	O; NR	M NSCLC	Nivolumab; Gemcitabine	IV	50	2020
PD-1	NCT04340882	O	M NSCLC; R NSCLC; A NSCLC	Docetaxel; Pembrolizumab; Ramucirumab	II	41	2020
PD-1	NCT04379635	O	NSCLC	Tislelizumab; Cisplatin; Paclitaxel; Pemetrexed; Carboplatin	III	380	2020
PD-1	NCT04396457	O	Non-SqCC; LC	Pembrolizumab; Pemetrexed	II	50	2020

PD-1	NCT04405674	O	NSCLC	Tislelizumab; Carboplatin; Pemetrexed	II	66	2020
PD-1	NCT04422392	O	NSCLC	Carboplatin; Pemetrexed; anti-PD-1	II	107	2020
PD-1	NCT04453930	O	SCLC	Camrelizumab; Platinum; Irinotecan; Apatinib	II	60	2020
PD-1	NCT04459078	O; NR	ADC	Camrelizumab; Paclitacxel; Apatinib	II	63	2020
PD-1	NCT04459611	O	NSCLC	Sintilimab; Pemetrexed; Carboplatin; Paclitaxel	II	60	2020
PD-1	NCT04490421	O	LC; SCLC	Camrelizumab; Apatinib; Etoposide; Cisplatin	III	45	2020
PD-1	NCT04506242	O; NR	NSCLC	Camrelizumab; Apatinib	II	74	2020
PD-1	NCT04507217	O; NR	A NSCLC; M NSCLC (BM)	Tislelizumab; Carboplatin; Pemetrexed	II	78	2020
PD-1	NCT04530227	O	E NSCLC	Camrelizumab; Pemetrexed; Paclitaxel	II	30	2020
PD-1	NCT04549025	O	NSCLC	JTX-4014; Vopratelimab	II	75	2020
PD-1	NCT04585815	O	Cancer; NSCLC	Sasanlimab; Encorafenib; Binimetinib; Sasanlimab; Axitinib; SEA-TGT	I; II	375	2020
PD-1	NCT04612673	O; NR	NSCLC	Sintilimab	II	33	2020

PD-1	NCT04613804	O; NR	A NSCLC	Toripalimab	II	30	2020
PD-1	NCT04620837	O	SCLC	Tislelizumab; Anlotinib	II	25	2020
PD-1	NCT04624204	O	SCLC	Pembrolizumab; Olaparib; Etoposide; Platinum; Radiation	III	672	2020
PD-1	NCT04691388	O	NSCLC	Amlotinib; Sindilumab	II	27	2020
PD-1	NCT04750083	O	Non-SqCC	HX008; Pembrolizumab; Pemetrexed; Cisplatin; Carboplatin	II; III	700	2020
PD-1	NCT04865705	O; NR	NSCLC	Tislelizumab	II	33	2020
PD-1	NCT04941417	O	NSCLC	Anti-PD-1	II	60	2020
PD-1	NCT04542369	O; NR	SCLC	Tislelizumab; Cisplatin; Etoposide	II	15	2021
PD-1	NCT04670913	O	A Non-SqCC	Camrelizumab; Apatinib	II	30	2021
PD-1	NCT04681131	O	NSCLC	CAB-AXL-ADC; anti-PD-1	II	240	2021
PD-1	NCT04683198	O; NR	SCLC	Camrelizumab; Apatinib; Carboplatin; Etoposide	II	69	2021
PD-1	NCT04725188	O	M NSCLC	Pembrolizumab; Vibostolimab; Docetaxel	II	240	2021
PD-1	NCT04728724	O; NR	NSCLC	Sintilimab; Chemotherapy	II	100	2021
PD-1	NCT04736823	O	NSCLC	AK112; Pemetrexed; Paclitaxel; Carboplatin; Docetaxel; AK105	II	206	2021

PD-1	NCT04749394	O; NR	A NSCLC	Camrelizumab; anti-PD-1; Apatinib; anti-VEGFR2	II	42	2021
PD-1	NCT04768075	O; NR	NSCLC	Camrelizumab; Cisplatin; Carboplatin; Pemetrexed; Paclitaxel	III	200	2021
PD-1	NCT04777084	O	NSCLC	IBI318	II	30	2021
PD-1	NCT04782089	O; NR	SCLC	Camrelizumab; anti-PD-1; Fluzoparib	NA	20	2021
PD-1	NCT04790539	O; NR	ES-SCLC	Camrelizumab	II	71	2021
PD-1	NCT04791839	O; NR	NSCLC	Zimberelimab; Domvanalimab; Etrumadenant	II	30	2021
PD-1	NCT04836728	O; NR	M NSCLC	CIK; Sintilimab; Pemetrexed; Paclitaxel; Carboplatin	II	156	2021
PD-1	NCT04875585	O; NR	NSCLC	Pembrolizumab; Lenvatinib	II	33	2021
PD-1	NCT04900363	O	NSCLC	AK112	I; II	360	2021
PD-1	NCT04901754	O; NR	SCLC	Camrelizumab; Apatinib	II	38	2021
PD-1	NCT04921358	O; NR	NSCLC	Tislelizumab; Sitravatinib; Docetaxel	III	420	2021
PD-1	NCT04924101	O; NR	SCLC	Pembrolizumab; MK-4830; MK-5890; Lenvatinib; Etoposide; Cisplatin; Carboplatin	II	120	2021

PD-1	NCT04938817	O; NR	NSCLC	Pembrolizumab; Quavonlimab; Lenvatinib; MK-4830; Favezelimab; Pembrolizumab	I; II	80	2021
PD-1	NCT04943029	O; NR	NSCLC	Carrelizumab; Pemetrexed; Paclitaxel; Carboplatin	II	30	2021
PD-1	NCT04736173	O	NSCLC; Non-SqCC; SqCC; LC	Zimberelimab; AB154; Carboplatin; Pemetrexed; Paclitaxel	III	625	2021
PD-1; PD-L1	NCT04672928	O	SCLC	Paclitaxel; IBI318	I	20	2020
PD-1; PD-L1	NCT04702009	O; NR	A LC; A NSCLC	Anti-PD-1; PD-L1; Chemotherapy	II; III	80	2021
PD-L1	NCT02273375	O; NR	NSCLC	Durvalumab	III	1360	2014
PD-L1	NCT02367794	C	SqCC	Atezolizumab; Carboplatin; Paclitaxel	III	1021	2015
PD-L1	NCT02400814	O; NR	R NSCLC; A NSCLC	Atezolizumab; Radiation	I	27	2015
PD-L1	NCT02463994	C	NSCLC	Atezolizumab; Radiation	Early-I	12	2015
PD-L1	NCT02486718	O; NR	NSCLC	Atezolizumab; Cisplatin; Vinorelbine; Docetaxel; Gemcitabine; Pemetrexed	III	1280	2015
PD-L1	NCT02576574	O; NR	NSCLC	Avelumab; Pemetrexed; Paclitaxel; Gemcitabine; Carboplatin; Cisplatin	III	1224	2015

PD-L1	NCT02572843	O; NR	NSCLC	Durvalumab	II	68	2016
PD-L1	NCT02657434	O; NR	NSCLC	Atezolizumab; Carboplatin; Cisplatin; Pemetrexed	III	578	2016
PD-L1	NCT02813785	O; NR	Cancer; NSCLC	Atezolizumab; Docetaxel	III	565	2016
PD-L1	NCT02994576	O	NSCLC	Atezolizumab	II	60	2016
PD-L1	NCT02879617	O	NSCLC	Durvalumab	II	50	2017
PD-L1	NCT02927301	O; NR	NSCLC	Atezolizumab	II	181	2017
PD-L1	NCT03003962	O; NR	NSCLC	Durvalumab; Paclitaxel; Carboplatin; Gemcitabine; Cisplatin	III	669	2017
PD-L1	NCT03014648	O	A NSCLC	Atezolizumab	II	111	2017
PD-L1	NCT03191786	O; NR	NSCLC	Atezolizumab; Vinorelbine; Gemcitabine	III	453	2017
PD-L1	NCT03334617	O	NSCLC	Durvalumab; AZD9150; AZD6738; Vistusertib; Olaparib; Oleclumab; Trastuzumab; Cediranib; Ceralasertib	II	410	2017
PD-L1	NCT03446547	O	E NSCLC	Durvalumab	II	106	2017
PD-L1	NCT03262454	O	R SCLC	Atezolizumab	II	35	2018

PD-L1	NCT03456063	O	NSCLC	Atezolizumab; Paclitaxel; Pemetrexed; Carboplatin; Cisplatin; Gemcitabine	III	450	2018
PD-L1	NCT03554473	O	Cancer; LC; SCLC	M7824; Topotecan; Temozolomide	I; II	67	2018
PD-L1	NCT03563716	O; NR	NSCLC	Atezolizumab; Tiragolumab;	II	135	2018
PD-L1	NCT03600701	O	M NSCLC; R NSCLC; A NSCLC	Atezolizumab; Cobimetinib	II	48	2018
PD-L1	NCT03620669	O	A NSCLC	Durvalumab	II	48	2018
PD-L1	NCT03631706	O; NR	NSCL	M7824; Pembrolizumab	III	584	2018
PD-L1	NCT03706690	O	Cancer; NSCLC	Durvalumab;	III	360	2018
PD-L1	NCT03789604	O; NR	NSCLC	CS1001	III	479	2018
PD-L1	NCT03800134	O	NSCLC	Durvalumab; Carboplatin; Paclitaxel; Cisplatin; Gemcitabine; Pemetrexed	III	800	2018
PD-L1	NCT03694236	O	E NSCLC	Durvalumab	I; II	39	2019
PD-L1	NCT03786692	O	NSCLC	Carboplatin; Pemetrexed; Bevacizumab; Atezolizumab	II	117	2019
PD-L1	NCT03833154	O	Cancer; NSCLC	Durvalumab; Radiation	III	706	2019
PD-L1	NCT03833440	O	NSCLC	Durvalumab; Monalizumab; Oleclumab; Ceralasertib; Docetaxel	II	120	2019

PD-L1	NCT03840902	O	NSCL	M7824; Durvalumab; Etoposide; Pemetrexed; Carboplatin; Paclitaxel; Cisplatin; Radiation	II	350	2019
PD-L1	NCT03896074	O; NR	NSCLC	Atezolizumab; Bevacizumab	II	206	2019
PD-L1	NCT03991403	O	NSCLC	Atezolizumab; Pemetrexed; Bevacizumab; Carboplatin; Paclitaxe; Cisplatin	III	228	2019
PD-L1	NCT04081688	O	R NSCLC; A NSCLC	Atezolizumab; Radiation; Varlilumab	I	15	2019
PD-L1	NCT04108026	O	A NSCLC	Durvalumab	II	67	2020
PD-L1	NCT04194203	O	Cancer; NSCLC	Atezolizumab; Bevacizumab; Paclitaxel; Pemetrexed; Carboplatin	III	306	2020
PD-L1	NCT04202809	O	NSCLC	Durvalumab	II	90	2020
PD-L1	NCT04224337	O	Cancer; NSCLC	Durvalumab; Doxorubicin; Ifosfamide	II	34	2020
PD-L1	NCT04234607	O; NR	A SCLC	TQB2450; Anlotinib; Etoposide; Carboplatin	III	738	2020
PD-L1	NCT04245514	O	NSCLC	Durvalumab; Radiation	II	90	2020
PD-L1	NCT04255836	O; NR	Cancer; NSCLC	Durvalumab; Chemotherapy; Radiation	II	35	2020

PD-L1	NCT04294810	O	NSCLC	Atezolizumab; Tiragolumab	III	560	2020
PD-L1	NCT04306926	O; NR	A NSCLC; M NSCLC	TQB2450; Radiation	II	59	2020
PD-L1	NCT04316364	O	NSCLC	SHR-1316; Paclitaxel; Carboplatin	III	456	2020
PD-L1	NCT04346914	O	ES-SCLC	Anti-PD-L1; Carboplatin; Etoposide	I	20	2020
PD-L1	NCT04385368	O	Cancer; NSCLC	Durvalumab; Chemotherapy	III	332	2020
PD-L1	NCT04392505	O	Cancer; NSCLC	Durvalumab	II	100	2020
PD-L1	NCT04396535	O	A NSCLC	Bintrafusp alfa; Docetaxel	II	80	2020
PD-L1	NCT04470674	O	LC; NSCLC (KRAS Mut)	Durvalumab; Carboplatin; Pemetrexed	II	50	2020
PD-L1	NCT04539977	O	SCLC	TQB2450	II	40	2020
PD-L1	NCT04549428	O	A NSCLC	Atezolizumab	II	20	2020
PD-L1	NCT04560244	O; NR	NSCLC	SHR1701	II	15	2020
PD-L1	NCT04560686	O	E NSCLC	Bintrafusp alfa	II	23	2020
PD-L1	NCT04562337	O; NR	ES-SCLC	SHR-1316; Chemotherapy; Radiation	II	67	2020
PD-L1	NCT04642469	O	Cancer; NSCLC	Durvalumab	III	284	2020
PD-L1	NCT04647357	O; NR	LS-SCLC	SHR-1316	II	60	2020
PD-L1	NCT04740021	O	SCLC	LP002; Carboplatin; Etoposide	II	46	2020
PD-L1	NCT04348292	O	NSCLC	Durvalumab; Sirolimus	I	31	2021
PD-L1	NCT04716946	O	NSCLC; LC	Durvalumab; Radiation	II	40	2021

PD-L1	NCT04776447	O; NR	LC; Thoracic cancer	Carboplatin; Placlitaxel; Atezolizumab	II	51	2021
PD-L1	NCT04786093	O; NR	NSCLC	Radiation; Durvalumab	II	52	2021
PD-L1	NCT04786964	O; NR	M Non-SqCC	Cosibelimab; Cisplatin; Carboplatin	III	560	2021
PD-L1	NCT04832854	O	NSCLC	Atezolizumab; Tiragolumab; Carboplatin; Cisplatin; Pemetrexed; Gemcitabine; Paclitaxel	II	82	2021
PD-L1	NCT04841538	O; NR	Thoracic cancer; NSCLC; SCLC	ES101	I; II	276	2021
PD-L1	NCT04866017	O	NSCLC	Tislelizumab; Durvalumab; Chemotherapy; Ociperlimab	III	900	2021
PD-L1	NCT04870112	O; NR	NSCLC; SCLC	Durvalumab; Cisplatin; Carboplatin; Etoposide	I; II	124	2021
PD-L1	NCT04878016	O; NR	ES-SCLC	ZKAB001; Carboplatin; Etoposide	III	498	2021
PD-L1	NCT04889066	O; NR	M NSCLC (BM)	Radiation; Durvalumab	II	40	2021
PD-L1	NCT04892953	O; NR	NSCLC	Carboplatin; Durvalumab; Gemcitabine; Paclitaxel; Pemetrexed	II	51	2021

Abbreviations: **C**, Closed; **O**, Open; **NR**, Not Recruiting; **NA**, Not Associated; **A**, Advanced; **M**, Metastatic; **R**, Recurrent; **T**, Terminated; **ES-SCLC**, Extensive-Stage Small Cell Lung Cancer; **LS-SCLC**, Limited-Stage Small Cell Lung Cancer; **LC**, Lung Cancer; **ADC**, Adenocarcinoma; **NSCLC**, Non Small Cell Lung Cancer; **SqCC**, Squamous Carcinoma.
