# Supplementary material for: Clinical Relevance of Targeted Therapy and Immune-Checkpoint Inhibition in Lung Cancer
Source: Pharmaceutics. 2023 Apr 16;15(4):1252. doi: 10.3390/pharmaceutics15041252 (PMC10142433; doi:10.3390/pharmaceutics15041252)
Supplement: Supplementary file 1 [file pharmaceutics-15-01252-s001.zip › Supplementary Table S3.pdf]

Supplementary Table S3. Ongoing clinical studies on targeted therapies and immune-checkpoint inhibitors in combination registered at clinicaltrials.gov

Targets	Study ID	Status	Conditions	Interventions	Phase	Enrollment	Start Date
ALK; PD-1	NCT04139317	O; NR	NSCLC	Capmatinib; Pembrolizumab	II	76	2020
ALK; PD-L1	NCT02898116	C	NSCLC; Cancer	Ensartinib; Durvalumab	I; II	2	2017
EGFR; CTLA4	NCT04141644	O	NSCLC (EGFR Mut)	Ipilimumab; Osimertinib	I	26	2020
EGFR; PD-1	NCT02323126	C	NSCLC	Nazartinib; Capmatinib; Nivolumab	II	64	2015
EGFR; PD-1	NCT02364609	O; NR	R NSCLC; A NSCLC	Afatinib; Pembrolizumab	I	38	2015
EGFR; PD-1	NCT04976647	O; NR	SqCC; NSCLC	HLX10; HLX07; Chemotherapy	II	156	2021
EGFR; PD-L1	NCT02013219	C	NSCLC	Alectinib; Atezolizumab; Erlotinib	I	52	2014
KRAS; PD-1	NCT04263090	O	NSCLC; ADC	Rigosertib; Nivolumab	I; II	30	2020
KRAS; PD-1	NCT04613596	O	A NSCLC; M Cancer	Adagrasib; Pembrolizumab	II	120	2020
MEK; PD-1	NCT03225664	O; NR	M NSCLC; R NSCLC; A NSCLC	Pembrolizumab; Trametinib	I; II	37	2018

MEK; PD-1	NCT03299088	O; NR	M Non-SqCC; R Non-SqCC; A NSCLC (KRAS Mut)	Pembrolizumab; Trametinib	I	15	2018
MEK; PD-1	NCT04323436	O	Cancer; NSCLC	Spartalizumab; Capmatinib	II	270	2020
MET; PD-1	NCT03647488	C	Cancer; NSCLC	Capmatinib; Spartalizumab; Docetaxel	II	18	2018
MET; PD-1	NCT03906071	O	M Non-SqCC	Nivolumab; Sitravatinib; Docetaxel	III	532	2019
MET; PD-L1	NCT04139317	O; NR	NSCLC	Capmatinib; Pembrolizumab	II	76	2020
PI3K; PD-1	NCT03257722	O	NSCLC; M NSCLC; R NSCLC	Pembrolizumab; Idelalisib	I; II	40	2017
ROS-1; CTLA-4; PD-1	NCT03468985	O; NR	M Non-SqCC; R Non-SqCC; A Non-SqCC	Cabozantinib; Ipilimumab; Nivolumab	II	169	2018

Abbreviations: **C**, Closed; **O**, Open; **NR**, Not Recruiting; **NA**, Not Associated; **A**, Advanced; **M**, Metastatic; **R**, Recurrent; **ADC**, Adenocarcinoma; **NSCLC**, Non-Small Cell Lung Cancer; **SqCC**, Squamous Carcinoma.
