# Supplementary material for: Clinical Relevance of Targeted Therapy and Immune-Checkpoint Inhibition in Lung Cancer
Source: Pharmaceutics. 2023 Apr 16;15(4):1252. doi: 10.3390/pharmaceutics15041252 (PMC10142433; doi:10.3390/pharmaceutics15041252)
Supplement: Supplementary file 1 [file pharmaceutics-15-01252-s001.zip › Supplementary Table S4.pdf]

Supplementary Table S4. Ongoing clinical studies on microbiota modulating drugs in combination with LC therapy registered at clinicaltrials.gov

Study ID	Status	Conditions	Interventions	Phase	Enrollment	Start Date
NCT02771470	C	LC	Probiotic (Clostridium butyricum); Chemotherapy	I	41	2014
NCT03775850	O	Cancer; NSCLC	EDP1503 (Bifidobacterium animalis lactis); Pembrolizumab	I; II	120	2018
NCT04009122	O	M NSCLC	IGEN0206 (Nutrient); standard of care	NA	280	2019
NCT03637803	O	Solid cancer; Melanoma; NSCLC	MRx0518 (live biotherapeutic product); Pembrolizumab	I; II	132	2019
NCT04601402	O	Solid cancer; NSCLC; SqCC	GEN-001 (live biotherapeutic product); Avelumab	I	93	2020
NCT04521075	O NR	A Melanoma; A NSCLC	FMT; Nivolumab	I; II	50	2020
NCT04924374	O NR	LC	FMT; anti-PD-1	NA	20	2021
NCT04105270	O NR	ADC; LC	Oral restorative microbiota therapy; Durvalumab; Cisplatin; Pemetrexed; Carboplatin	II	30	2021
NCT03819296	O	Solid cancer; NSCLC	FMT; Infliximab; Prednisone; Vedolizumab	I; II	800	2021

Abbreviations: **C**, Closed; **O**, Open; **NR**, Not Recruiting; **NA**, Not Associated; **A**, Advanced; **M**, Metastatic; **R**, Recurrent; **LC**, Lung Cancer; **ADC**, Adenocarcinoma; **NSCLC**, Non-Small Cell Lung Cancer.
